# Evaluation of *Urtica dioica* Phytochemicals against Therapeutic Targets of Allergic Rhinitis Using Computational Studies

**DOI:** 10.3390/molecules29081765

**Published:** 2024-04-12

**Authors:** Erick Bahena Culhuac, Martiniano Bello

**Affiliations:** 1 Laboratorio de Diseño y Desarrollo de Nuevos Fármacos e Innovación Biotecnológica, Escuela Superior de Medicina, Instituto Politécnico Nacional, Ciudad de México 11340, Mexico; erickb_2000@hotmail.com; 2 Facultad de Ciencias, Universidad Autónoma del Estado de México, Toluca 50000, Mexico

**Keywords:** molecular dynamics simulation, allergic rhinitis, *Urtica dioica*, docking, MMGBSA

## Abstract

Allergic rhinitis (AR) is a prevalent inflammatory condition affecting millions globally, with current treatments often associated with significant side effects. To seek safer and more effective alternatives, natural sources like *Urtica dioica* (UD) are being explored. However, UD’s mechanism of action remains unknown. Therefore, to elucidate it, we conducted an in silico evaluation of UD phytochemicals’ effects on known therapeutic targets of allergic rhinitis: histamine receptor 1 (HR1), neurokinin 1 receptor (NK1R), cysteinyl leukotriene receptor 1 (CLR1), chemoattractant receptor-homologous molecule expressed on type 2 helper T cells (CRTH2), and bradykinin receptor type 2 (BK2R). The docking analysis identified amentoflavone, alpha-tocotrienol, neoxanthin, and isorhamnetin 3-O-rutinoside as possessing a high affinity for all the receptors. Subsequently, molecular dynamics (MD) simulations were used to analyze the key interactions; the free energy of binding was calculated through Generalized Born and Surface Area Solvation (MMGBSA), and the conformational changes were evaluated. Alpha-tocotrienol exhibited a high affinity while also inducing positive conformational changes across all targets. Amentoflavone primarily affected CRTH2, neoxanthin targeted NK1R, CRTH2, and BK2R, and isorhamnetin-3-O-rutinoside acted on NK1R. These findings suggest UD’s potential to treat AR symptoms by inhibiting these targets. Notably, alpha-tocotrienol emerges as a promising multi-target inhibitor. Further in vivo and in vitro studies are needed for validation.

## 1. Introduction

Allergic rhinitis (AR) is a common inflammatory condition, affecting up to 30% of the global population [1]. It manifests symptoms such as sneezing, nasal congestion, and runny nose [2]. AR happens when allergens activate certain parts of the immune system, specifically immunoglobulin E (IgE) and type 2 helper T (Th2) cells [3]. Although often perceived as mild, AR significantly impacts patients’ lives, as numerous studies have shown [4,5,6]. Poor management due to misdiagnosis or inadequate treatment exacerbates patients’ difficulties [2,7]. There are two main treatments for AR, intranasal glucocorticoids (GCs) and antihistamines. GCs are highly effective anti-inflammatory and immunosuppressive agents [8]. However, they come with potential long-term side effects, including osteonecrosis, osteoporosis, myopathy, hyperglycemia, and weight gain [9]. Meanwhile, antihistamines, the most commonly used AR treatment, provide moderate relief from symptoms. Therefore, they often require supplementation with intranasal GCs or other therapies for better results [8]. Given the prevalent side effects and economic burdens of AR treatments, there is growing interest in exploring alternative therapies, particularly plant-derived ones. Plant-based medicine, the primary healthcare method for over 4 billion people around the world [10], can alleviate AR symptoms without significant side effects [3,11]. However, a deeper understanding of the mechanisms of action and the specific phytochemicals involved is crucial for developing newer, more affordable, and efficient treatments.

One of the most promising plants deserving further exploration is Urtica dioica (UD), commonly known as stinging nettle. UD can be found in Europe, North America, North Africa, and Asia [12,13]. Historically, it has had a wide range of traditional uses, such as alleviating joint pain, anemia, arthritis, gout, cardiovascular symptoms, diabetes, arthritis, and AR [12,13]. UD’s efficacy in alleviating AR symptoms has been evident since the early 1990s [14]. Recent clinical trials involving UD have corroborated its effectiveness in reducing the severity of clinical symptoms and nasal eosinophil counts [15]. However, the fact that UD does not induce significant changes in IgE levels has prompted questions regarding its mechanism of action. To this point, UD extracts can inhibit HR1 and key enzymes in prostaglandin formation [16]. Furthermore, UD could attenuate inflammatory cell recruitment in an asthmatic rat model [17].

Nevertheless, questions and inconsistencies regarding its mechanism of action and overall efficacy persist. One of the most common problems with treatments based on plant extracts is the significant variability in their phytochemical content. This variability can result from different harvesting seasons, external stimuli, types of tissues collected, postharvest changes, and extraction techniques [18,19,20,21,22]. This variability is particularly notable within the context of UD due to its problematic taxonomic classification [23,24]. As UD is found in various parts of the globe, there is an effort to reclassify UD into subspecies [23,24], a consideration often overlooked in many studies. Thus, numerous factors could alter the phytochemical contents, potentially contributing to the inconsistencies observed in UD treatment outcomes.

With this aim in mind, our study sought to evaluate the effectiveness of UD in treating AR by examining the affinity of UD phytochemicals for specific treatment targets. Through this investigation, we sought to enhance our comprehension of UD’s mechanism of action, efficacy, and the underlying molecules responsible for alleviating AR symptoms. HR1, NK1R, CLR1, CRTH2, and BK2R were tested against UD phytochemicals through docking analyses to achieve these objectives. The top five molecules with the highest affinity for all receptors were chosen for a triplicated MD simulation of 100 ns. Consequently, protein–ligand complex interactions were analyzed, and their affinity was evaluated using MMGBSA. In addition, a detailed analysis of structural changes was conducted to detect the phytochemicals’ ability to inhibit said targets. Notably, alpha-tocotrienol exhibited high-affinity interactions with all the receptors and structural changes similar to antagonists of these receptors. This highlights alpha-tocotrienol as a potential multi-target inhibitor for AR symptoms. These findings hold promise for advancing our understanding of UD’s therapeutic potential and may contribute to developing novel and more effective AR treatments.

## 2. Results and Discussion

### 2.1. Molecular Docking of UD Database against AR Targets

Initially, a database was established using reported UD phytochemical structures. This was followed by a literature search for AR pharmacological targets. Due to the limited passive diffusion of phytochemicals through cell membranes [25], this research was focused on G protein-coupled receptors (GPCRs) rather than intracellular receptors. Therefore, we selected five GPCRs with crystallized structures and known inhibitors. Consequently, HR1, NK1R, CLR1, CRTH2, and BK2R were chosen as target receptors. HR1 triggers diverse AR responses, including smooth muscle contraction, respiratory dilation, NF-kB pathway activation, and the synthesis of various factors [26,27,28]. NK1R activation is linked to proinflammatory responses and airway inflammation [29,30]. CLR1 contributes to nasal obstruction and airway inflammation [31], while CRTH2 induces vasodilation, nasal obstruction, and inflammation [32]. Meanwhile, K2R mediates increased vascular permeability, nasal obstruction, and nerve stimulation [33,34].

The molecular docking targeting the HR1 receptor was configured using the binding pocket identified from reported inhibitor binding sites [35,36,37,38]. Known inhibitors, including fexofenadine, loratadine, and doxepin, were also docked to comprehensively compare the affinity values. Fexofenadine exhibited the highest mean affinity value at −7.31 kcal/mol. The affinity values of the phytochemicals and inhibitors can be found in Table S1. Notably, four phytochemicals surpassed fexofenadine in affinity: 7α-hydroxy-sitosterol (−7.46 kcal/mol), β-sitosterol (−7.38 kcal/mol), γ-sitosterol (−7.33 kcal/mol), and phytosterol (7.33 kcal/mol).

In the case of NKR1, the binding pocket was set based on the reported interaction with the known inhibitor aprepitant [39]. The affinity values for all the phytochemicals are presented in Table S2, showcasing the top 15 phytochemicals, with the affinities ranging from −7.95 kcal/mol to −9.6 kcal/mol. Amentoflavone demonstrated the highest affinity at −9.6 kcal/mol, surpassing the known inhibitor aprepitant, which had an affinity of −9.03 kcal/mol (Table 1). Regarding CLR1, the chosen inhibitor was zafirlukast, and the binding pocket was also determined based on its interaction [40]. The affinity values and their mean, obtained from both software, are detailed in Table S3. Zafirlukast exhibited the highest affinity against CLR1 at −11.29 kcal/mol. Moreover, there was a significant discrepancy between the affinity values of zafirlukast and the UD phytochemicals. Amentoflavone (−9.56 kcal/mol) demonstrated the closest binding affinity to zafirlukast (Table 1).

The CRTH2 binding pocket was defined by its interactions with a PGD2 derivative (15mPGD2) and fevipiprant [41]. Fevipiprant served as the known inhibitor for CRTH2, and the corresponding affinity values are detailed in Table S4. Fevipiprant exhibited a mean affinity value of −9.2 kcal/mol. Notably, only two phytochemicals surpassed this affinity value: neoxanthin (−9.52 kcal/mol) and alpha-tocotrienol (−9.25 kcal/mol) (Table 1). The BK2R binding pocket was established based on documented interactions with bradykinin [42,43]. JSM10292 served as the selected known inhibitor, and the associated affinity values are detailed in Table S5. JSM10292 demonstrated a binding affinity of −8.375 kcal/mol. In this case, six phytochemicals exhibited higher affinity values than JSM10292: isorhamnetin rutinoside (−8.718 kcal/mol), heptadecanoic acid (−8.647 kcal/mol), 7β-hydroxy-sitosterol (−8.645 kcal/mol), γ-sitosterol (−8.603 kcal/mol), 7α-hydroxy-sitosterol (−8.569 kcal/mol), and amentoflavone (−8.384 kcal/mol) (Table 1).

The outcomes from the five dockings underscored that UD comprises phytochemicals with comparable or, in certain instances, superior affinity compared to established inhibitors. Additionally, we noted certain phytochemicals exhibiting a high affinity across multiple receptors (Tables S6–S10). To systematically assess this, we computed the average values for each phytochemical from the five dockings and identified their presence in the top 15 highest binding affinities in each docking (Table S11). Notably, our analysis revealed the presence of four phytochemicals consistently ranking within the top 15 in four out of the five dockings: amentoflavone, alpha-tocotrienol, neoxanthin, and isorhamnetin 3-O-rutinoside (Table 1). The existence of phytochemicals with a high affinity for more than one pharmacological target presents a great advantage. Multiple reports highlight the benefits of combining therapies for AR treatment [44], such as the inhibition of HR1 and NKR1 [45] or HR1 and CLR1 [46]. Therefore, we performed MD simulations to further analyze the ability of amentoflavone, alpha-tocotrienol, neoxanthin, and isorhamnetin 3-O-rutinoside to function as antagonists of the five selected targets.

Furthermore, alpha-tocotrienol, cholecalciferol, hecogenin, and solanidine were identified in Roschek Jr. et al.’s screen (Table 1). Notably, despite Ayers and Roschek Jr. using UD from Blessed Herbs and HPLC-grade water for extraction, Ayers’ screen did not find these phytochemicals. Moreover, Roschek Jr. et al. used the extract to test its effects on key receptors and enzymes linked to allergic rhinitis. Thus, it is encouraging that some of those molecules are found within this table [16]. Additionally, kaempferol-3-rutinoside, isorhamnetin rutinoside, epigallocatechin gallate, and epicatechin gallate were identified in the screen conducted by Repajić et al. [47], which involved a diverse array of plant samples from various regions in Croatia. Isorhamnetin 3-O-rutinoside and kaempferol-3-rutinoside were also detected in the study by Pinelli et al. [48], while isorhamnetin rutinoside appeared in Garcia et al.’s research [49,50]. Both studies focused on plants from Tuscany. Finally, the remaining six molecules were featured in the review by Grauso et al. [50]. It is worth noting that the six molecules also originate from samples primarily sourced from Europe [51,52,53,54]. However, due to the lack of comparison with samples from other regions, it remains uncertain if there is any specific regional tendency.

### 2.2. Stability and Equilibrium of Receptor–Ligand Complexes

In all systems, the negative control (flavoxate) consistently dissociated. However, despite its structural similarity to amentoflavone, it failed to establish the same stability with the receptors. This underscores the credibility of the interactions formed by the phytochemicals. Conversely, the NKR1, CLR1, CRTH2, and BK2R positive controls, and all the phytochemicals exhibited stable interactions with their respective complexes. However, for HR1, both neoxanthin and the known inhibitor (fexofenadine) dissociated. The dissociation of fexofenadine from HR1 might suggest a potentially higher affinity for HR1 among these three compounds. While no experimental structure or direct observation of the fexofenadine–HR1 interaction exists, MD simulations [55] and flexible ligand–receptor docking experiments [37] have reported that fexofenadine binds to Asp107, Tyr108, Lys179, Lys191, and Tyr458. This aligns with our docking parameters (Table 1). Thus, the main difference between the methodologies of those studies and ours is the binding pocket size. The methodologies mentioned above defined their binding pocket based on the interactions generated by doxepin with HR1 in the experimental structure PDB:3RZE. Here, we established the binding pocket based on PDB:3RZE and other computational and mutagenesis analyses [35,36,37,38]. Coupled with using PDB:7DFL as the base HR1 structure, this enabled us to have a more detailed and realistic binding pocket.

The RMSD analysis revealed that equilibrium was typically attained within 30 to 100 ns across most receptor–ligand complexes, as illustrated in Figure S1. The observed fluctuations ranged from 2 to 4 Å in the HR1 complexes (Figure S1A), 2 to 12 Å in the NKR1 complexes (Figure S1B), 2 to 7 Å in the CLR1 complexes (Figure S1C), 3 to 8 Å in CRTH2 (Figure S1D), and 2 to 5 Å in BK2R (Figure S1E). The convergence of the Rg values was observed between 40 and 100 ns (Figure S1), with values spanning from 20 to 22 Å in the HR1 complexes (Figure S1F), 27 to 31 Å in the NKR1 complexes (Figure S1G), 29 to 33 Å in the CLR1 complexes (Figure S1H), 28 to 31 Å in CRTH2 (Figure S1I), and 21 to 23 Å in BK2R (Figure S1J). Furthermore, the RMSF analysis demonstrated that all the phytochemicals exhibited a magnitude of change comparable to that of the known inhibitors, with differences of less than 2 Å (Figure S2). This observation suggests that the phytochemicals achieved stability in the same region as the known inhibitor, eliciting similar structural changes.

### 2.3. Analysis of Protein–Ligand Complexes

**Figure 1:**
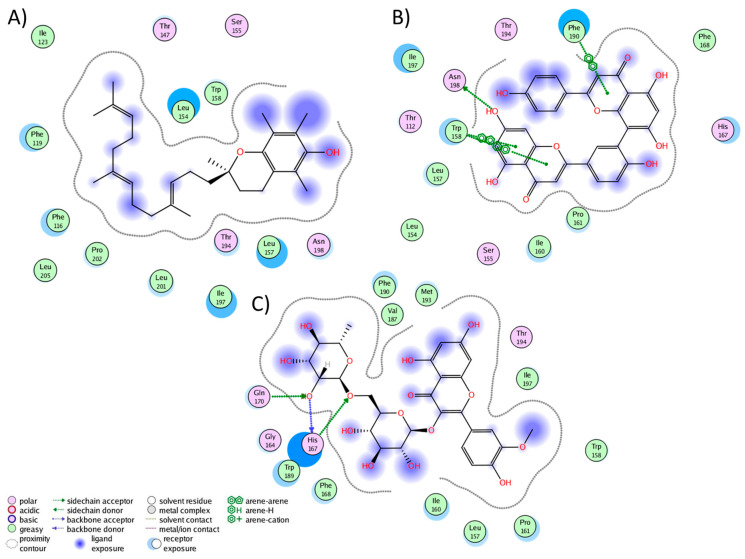
Graphical representation of the interactions between HR1 and (**A**) alpha-tocotrienol, (**B**) amentoflavone, and (**C**) isorhamnetin 3-O-rutinoside.

We conducted a cluster analysis to analyze the key interactions between the phytochemicals and HR1 during the simulation. Within most population structures, alpha-tocotrienol demonstrated 14 interactions with HR1 (Figure 1A). Specifically, residues Trp158, Thr194, and Asn198 were reported to interact with doxepin [37], while Trp158 and Asn198 interact with histamine [56]. Amentoflavone exhibited interactions with 13 residues (Figure 1B), including Thr112, Trp158, and Asn198, which also interacted with doxepin, and Thr112 and Asn198, which interact with histamine [37,56]. Isorhamnetin-3-O-rutinoside demonstrated interactions with 14 residues (Figure 1C), with Trp158 and Thr194 also interacting with doxepin but none of the residues interacted with histamine. Notably, none of the residues interacted with Asp107, Tyr108, or those surrounding residue 400. Thus, while these molecules occupy the desired binding pocket, the docking analysis revealed greater stability around TM3, TM4, and TM5 [56].

**Figure 2:**
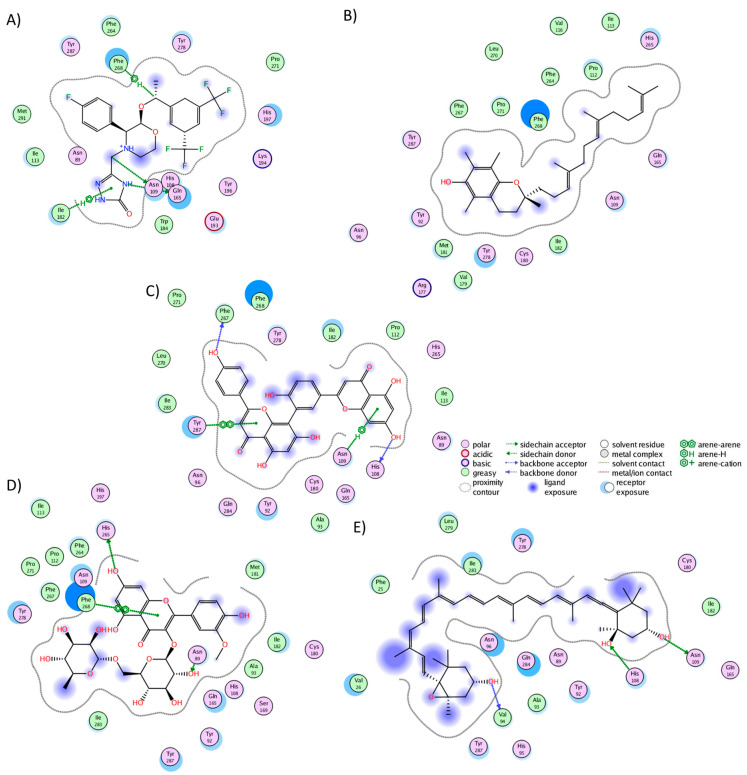
Graphical representation of the interactions of NKR1 in complex with (**A**) aprepitant, (**B**) alpha-tocotrienol, (**C**) amentoflavone, (**D**) isorhamnetin-3-O-rutinoside, and (**E**) neoxanthin.

The cluster analysis of the NKR1 complexes revealed that aprepitant established interactions with 17 residues, forming five hydrogen bonds with His108, Asn109, Ile182, Gln165, and Phe268 (Figure 2A). Notably, nine of these interactions are consistent with literature findings: Asn109, Ile113, Gln165, Ile182, Trp184, Glu193, His197, Phe264, and Phe268 [39]. It is worth noticing that Gln165 and Tyr287 play a pivotal role in both agonist and antagonist binding [57]. Alpha-tocotrienol engaged with 20 residues (Figure 2B), with nine interactions overlapping with aprepitant. Amentoflavone also interacted with 20 residues (Figure 2C), forming hydrogen bonds with His108, Asn109, Phe267, and Tyr287. Seven of these interactions mirrored those observed with aprepitant. Isorhamnetin-3-O-rutinoside formed interactions with 21 residues (Figure 2D), including three hydrogen bonds (Asn89, His265, Phe268), with nine shared interactions with aprepitant. Neoxanthin engaged with 18 residues (Figure 2E), establishing three hydrogen bonds (Val94, His108, Asn109) and sharing three interactions with aprepitant. Notably, neoxanthin shifted slightly toward TM1 and TM2 but remained within the desired binding pocket.

**Figure 3:**
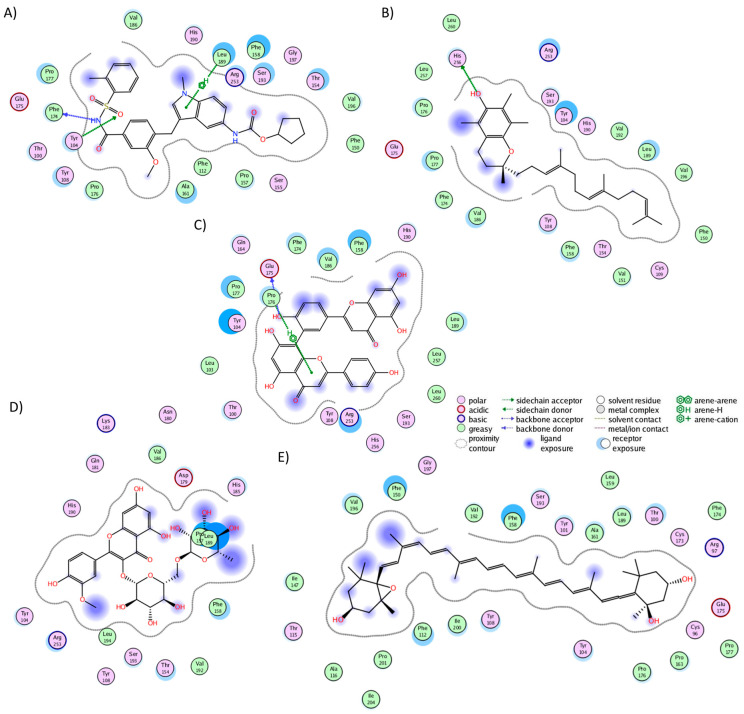
Graphical representation of the interactions of CLR1 in complex with (**A**) zafirlukast, (**B**) alpha-tocotrienol, (**C**) amentoflavone, (**D**) isorhamnetin-3-O-rutinoside, and (**E**) neoxanthin.

The cluster analysis of the CLR1 complexes illustrated that zafirlukast established interactions with 21 residues (Figure 3A), forming hydrogen bonds with Tyr104, Phe174, and Leu189. In the experiment, zafirlukast interacted with 16 residues: Tyr104, Tyr108, Thr154, Ser155, Pro157, Phe158, Pro176, Leu189, His190, Ser193, Tyr249, Arg253, His256, Val277, Thr280, and Leu281 [40]. Notably, 11 out of the 16 interactions from the experimental structure were recapitulated in the MD simulation. Similarly, alpha-tocotrienol generated interactions with 21 residues (Figure 3B), with a hydrogen bond observed with His256. Fifteen of these interactions overlapped with those of zafirlukast. Amentoflavone engaged with 18 residues (Figure 3C), forming hydrogen bonds with Glu175 and Pro176, and 13 of these interactions were shared with zafirlukast. Isorhamnetin-3-O-rutinoside interacted with 17 residues (Figure 3D), with no hydrogen bonds formed and ten interactions were common with zafirlukast. Finally, neoxanthin established interactions with 28 residues (Figure 3E) without hydrogen bonds. The extensive interactions of neoxanthin were attributed to its significant stretching inside CLR1. Notably, 15 out of these 28 interactions were also observed in zafirlukast. Importantly, none of the molecules deviated from the desired binding pocket, maintaining interactions similar to those of the known inhibitor.

**Figure 4:**
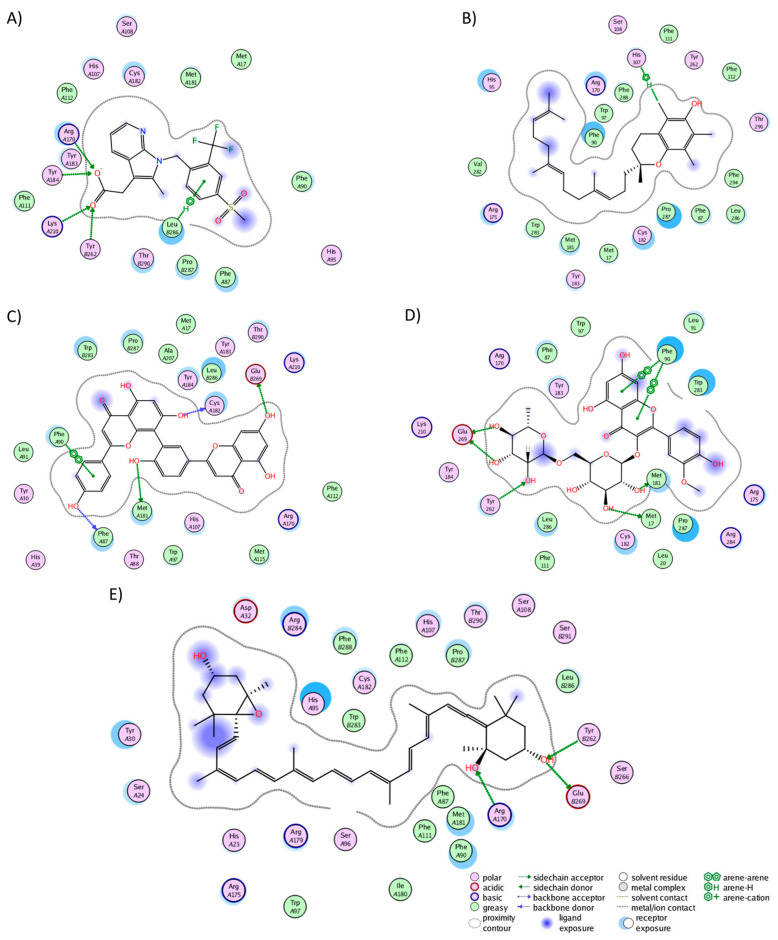
Graphical representation of the interactions of CRTH2 in complex with (**A**) fevipiprant, (**B**) alpha-tocotrienol, (**C**) amentoflavone, (**D**) isorhamnetin-3-O-rutinoside, and (**E**) neoxanthin.

In the CRTH2 cluster analysis, fevipiprant formed interactions with 19 residues (Figure 4A), establishing hydrogen bonds with His107, Tyr184, Lys210, and Tyr262. Fourteen of these interactions aligned with those observed in the experimental structure (Met17, Phe87, His107, Phe111, Phe112, Arg170, Cys182, Tyr183, Tyr184, Lys210, Tyr262, Leu286, Pro287, and Thr290), where, notably, Tyr184, Lys210, and Tyr262 also participated in hydrogen bonding [58]. This consistency between the experimental and MD analyses underscores the preservation of fevipiprant’s binding pocket. Alpha-tocotrienol engaged with 22 residues (Figure 4B), forming a single hydrogen bond with His107, while 15 interactions coincided with those observed in fevipiprant. Amentoflavone established interactions with 20 residues (Figure 4C), featuring five hydrogen bonds (Phe87, Phe90, Met181, Cys182, and Glu269). Additionally, 17 of these interactions were shared with fevipiprant. Isorhamnetin-3-O-rutinoside interacted with 20 residues (Figure 4D), with four hydrogen bonds (Met17, Phe90, Met181, Tyr262, and Glu269) and 13 interactions were in common with fevipiprant. Lastly, neoxanthin displayed interactions with 29 residues, the highest among the molecules (Figure 4E). However, only 3 of these interactions formed hydrogen bonds (Arg170, Tyr262, and Glu269), yet 16 interactions were shared with fevipiprant.

**Figure 5:**
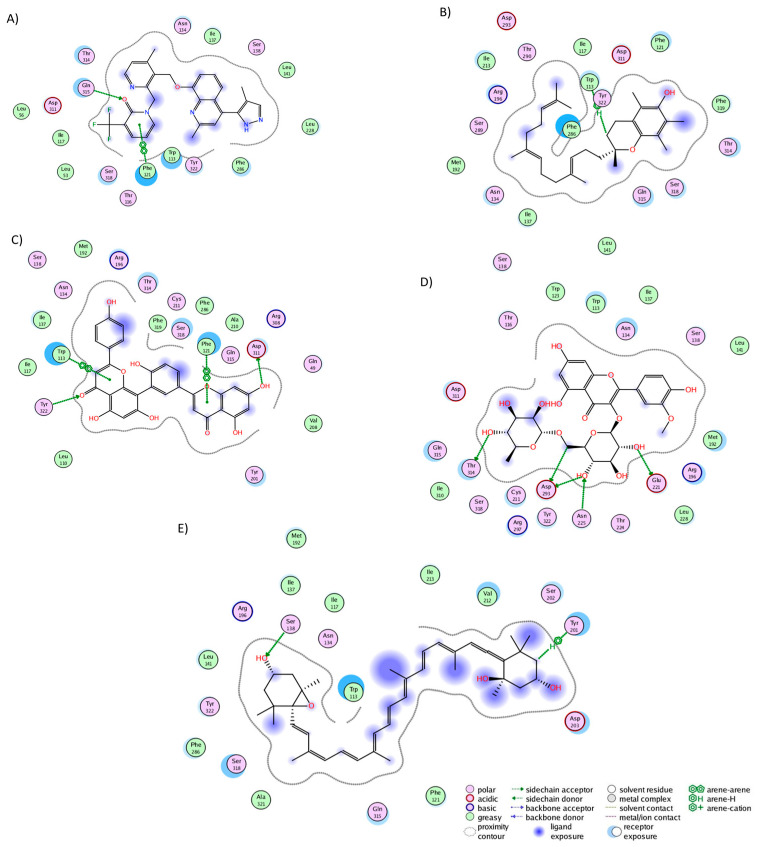
Graphical representation of the interactions of BK2R in complex with (**A**) JSM-10292, (**B**) alpha-tocotrienol, (**C**) amentoflavone, (**D**) isorhamnetin-3-O-rutinoside, and (**E**) neoxanthin.

In the BK2R cluster analysis, JSM-10292 established interactions with 17 residues (Figure 5A), forming a single hydrogen bond with Phe121. Notably, ten of these interactions (Trp113, Phe121, Asn134, Ile137, Ser138, Leu141, Leu228, Phe286, Asp311, and Thr314) coincided with the binding pocket of bradykinin and JSM10292, as reported in previous studies [42,43]. Alpha-tocotrienol engaged with 20 residues (Figure 5B), generating a lone hydrogen bond with Trp113. Nine of these interactions were also observed in JSM-10292. Amentoflavone formed interactions with 22 residues (Figure 5C) and established four hydrogen bonds with Trp113, Phe121, Asp311, and Tyr322. Of these residues, 12 were shared with JSM-10292. Isorhamnetin-3-O-rutinoside interacted with 22 residues (Figure 5D), creating hydrogen bonds with Glu221, Asn225, Asp293, and 314. Twelve of these interactions overlapped with JSM-10292. Neoxanthin interacted with 19 residues (Figure 5E), producing three hydrogen bonds (Ser138 and Tyr201), with 11 of these interactions shared with JSM-10292. In summary, all molecules established stable interactions within the desired binding pocket, primarily interacting with TM3, TM4, the loop between both, and TM7. In conclusion, the comprehensive analysis revealed that all the examined phytochemicals consistently established stable interactions within the binding pockets of their respective receptors. Notably, many of these interactions mirrored those observed with known inhibitors, reinforcing their potential as ligands with interactions comparable to those of known inhibitors.

### 2.4. Binding Free Energy of UD Phytochemicals

To comprehensively assess the affinity of the phytochemicals for the target, we employed the MMGBSA approach to calculate the binding free energies. HR1 and alpha-tocotrienol exhibited the highest affinity among the compounds studied, yielding a binding free energy of −38.493 (±5.112) kcal/mol. Amentoflavone and isorhamnetin 3-O-rutinoside demonstrated comparable binding free energies of −28.679 (±3.301) kcal/mol and −26.446 (±4.216) kcal/mol, respectively (Table 2). An in-depth analysis of the per-residue decomposition of the free energy revealed that alpha-tocotrienol formed 11 interactions above the 0.5 kcal/mol cutoff. At the same time, amentoflavone generated 12, and isorhamnetin 3-O-rutinoside had 10 interactions (Table S12). The discernible difference in the affinity of alpha-tocotrienol compared to the other phytochemicals can be attributed to its ability to establish a higher number of interactions, albeit with lower affinities.

For NKR1, isorhamnetin-3-O-rutinoside had the highest binding affinity of all the molecules with −46.756 (±4.78) kcal/mol, followed by alpha-tocotrienol with −45.400 (±3.28) kcal/mol, neoxanthin with −39.455 (±7.95) kcal/mol, aprepitant with −38.343 (±3.61) kcal/mol, and amentoflavone with −33.954 (±3.70) kcal/mol (Table 2). Notably, only amentoflavone failed to surpass the affinity of the known inhibitor, aprepitant. The per-residue decomposition of the free energy highlighted isorhamnetin 3-O-rutinoside as the molecule generating the highest number of interactions above the 0.5 kcal/mol threshold, with a count of 19. This was followed by neoxanthin with 16, and amentoflavone and alpha-tocotrienol with 14 interactions, while aprepitant exhibited 11 interactions (Table S13). Thus, once again, the highest affinity was more associated with the number of interactions than with the magnitude of the affinity.

For CLR1, neoxanthin displayed the highest binding affinity at −61.436 (±5.20) kcal/mol, with alpha-tocotrienol closely following at −54.785 (±2.89) kcal/mol, which is consistent with the cluster analysis. Notably, neoxanthin and alpha-tocotrienol surpassed the binding affinity of zafirlukast (−49.684 (±4.43) kcal/mol). In the per-residue decomposition of the free energy, neoxanthin emerged as the molecule generating the highest number of interactions above the 0.5 kcal/mol threshold, with an impressive count of 24. Alpha-tocotrienol generated the second-highest number of interactions at 18, followed by isorhamnetin-3-O-rutinoside with 17 interactions, zafirlukast with 16, and amentoflavone with 14 (Table S14). Here, the remarkable binding affinity of neoxanthin towards CLR1 can be attributed to the substantial 24 interactions above the 0.5 kcal/mol threshold. Thus, once again, the high affinity is due to the high number of interactions with lower affinity.

For CRTH2, neoxanthin exhibited the highest binding affinity at −56.836 (±4.22) kcal/mol, followed by alpha-tocotrienol at −53.334 (±4.96) kcal/mol, amentoflavone at −45.005 (±3.37) kcal/mol, fevipiprant at −43.221 (±4.58) kcal/mol, and isorhamnetin-3-O-rutinoside at −41.325 (±6.32) kcal/mol (see Table 2). Notably, only isorhamnetin-3-O-rutinoside failed to surpass the binding affinity of the known inhibitor, fevipiprant. In the per-residue decomposition of the free energy, amentoflavone and alpha-tocotrienol displayed the highest number of interactions above the cutoff, each with 17. Despite amentoflavone and alpha-tocotrienol sharing the same number of interactions above 0.5 kcal/mol, alpha-tocotrienol displayed an energy higher by 8 kcal/mol. Then, isorhamnetin-3-O-rutinoside and fevipiprant exhibited the second-highest number of interactions above the cutoff, with 15, while neoxanthin generated the lowest number of interactions at 14 (Table S15). This suggests that neoxanthin achieves its affinity through many interactions below 0.5 kcal/mol.

For BK2R, consistent with the cluster analysis findings, all the phytochemicals exhibited higher binding affinity than JSM-10292 at −32.310 (±4.73) kcal/mol. Neoxanthin demonstrated the highest affinity towards the binding pocket at −47.394 (±4.81) kcal/mol, followed by alpha-tocotrienol at −42.654 (±3.71) kcal/mol, amentoflavone at −38.984 (±5.37) kcal/mol, and isorhamnetin-3-O-rutinoside at −37.080 (±4.76) kcal/mol (refer to Table 2). In the per-residue decomposition of the free energy, isorhamnetin-3-O-rutinoside produced the highest number of interactions above the cutoff, with 17, followed by alpha-tocotrienol with 15. JSM-10292 and neoxanthin each had 13 interactions, while amentoflavone had 12 (Table S16).

Alpha-tocotrienol’s superior affinity compared to all the known inhibitors prompted further analysis. The binding free energies for alpha-tocotrienol and the known inhibitors were recalculated using the MMPBSA methodology (Table 3). For HR1, in which, the known inhibitor dissociated, amentoflavone was used for comparison, revealing an affinity of −34.65 (±4.7) kcal/mol for alpha-tocotrienol and −28.2 (±3.81) kcal/mol for amentoflavone. Both molecules maintained the observed difference seen with MMGBSA. For NKR1, CLR1, and BK2R, alpha-tocotrienol consistently exhibited better binding free energies than the known inhibitor, affirming its higher affinity. For NKR1, the affinity was −35.7 (±4.65) kcal/mol for alpha-tocotrienol versus −29.52 (±5.01) kcal/mol for aprepitant. For CLR1, the affinity was −45.55 (±3.07) kcal/mol for alpha-tocotrienol versus −38.95 (±4.65) kcal/mol for zafirlukast. For BK2R, the affinity was −31.93 (±5.38) kcal/mol for alpha-tocotrienol versus −23.77 (±4.12) kcal/mol for JSM-10292. Only for CRTH2 did alpha-tocotrienol (−31.73 ± 4.33 kcal/mol) exhibit a lower affinity than the known inhibitor fevipiprant (−52.55 ± 5.93 kcal/mol). Alpha-tocotrienol maintained a superior affinity within all systems, but the results for CRTH2 are encouraging [59]. Even for CRTH2, where its affinity was reduced, alpha-tocotrienol remained a viable antagonist, as its binding free energy aligned with that of other inhibitors and its values in the different systems.

Consequently, alpha-tocotrienol demonstrated a higher affinity and stability than most known inhibitors, consistently ranking as the highest or second highest across most systems analyzed using the MMGBSA and MMPBSA methodologies. However, it is noteworthy that alpha-tocotrienol did not maintain this superiority towards the known inhibitor for CRTH2 according to the MMPBSA calculations. However, its value remained as high as in other systems. Meanwhile, neoxanthin exhibited a higher affinity than the known inhibitors in all systems except for HR1. Finally, isorhamnetin-3-O-rutinoside had a higher affinity than the known inhibitors for NKR1 and BK2R, while amentoflavone’s affinity was higher for BK2R and CRTH2. A further analysis of the per-residue decomposition showed that the difference between alpha-tocotrienol and the known inhibitors or other phytochemicals was attributed to alpha-tocotrienol generating more interactions with an affinity lower than 0.5 kcal/mol.

### 2.5. Evaluation of Structural Inactivation of Target Receptors

One key benefit of studying GPCRs is their propensity to undergo distinct and well-documented structural alterations upon interaction with G proteins. This characteristic enables us to discern the potential effects induced by phytochemicals that may trigger agonistic or antagonistic conformational shifts. In most GPCRs, the agonists bind to the receptor’s extracellular domain, initiating a cascade of conformational changes that draw the TMs closer within the extracellular region. This process forms an intracellular cavity that facilitates G protein binding [60]. Conversely, antagonists exert the opposite effect by pushing TMs away within the extracellular portion and closing the intracellular cavity. Notably, conformational changes within TM2, TM3, TM5, TM6, and TM7 are commonly observed across various systems. However, CRTH2 stands apart as it operates without conventional activation [41,61]. Molecular dynamics studies have revealed consistent transmembrane conformations among antagonists, agonists, and CRTH2 alone [41,58,62], except for the behavior of helix 8 [61,62]. This helix, upon binding of an agonist, will undergo compaction to inhibit arrestin recruitment.

To evaluate these conformational changes, we analyzed the phytochemicals that exhibited a higher affinity than the known inhibitors. This is performed by comparing their structures to those derived from experimental studies alongside the simulations with the known inhibitors. First, a preliminary analysis was conducted based on the most populated cluster structure (Table 4). Complexes exhibiting conformational changes similar to those of the known inhibitors underwent further scrutiny by analyzing the structural changes within the 100 ns of the MD simulation.

**Figure 6:**
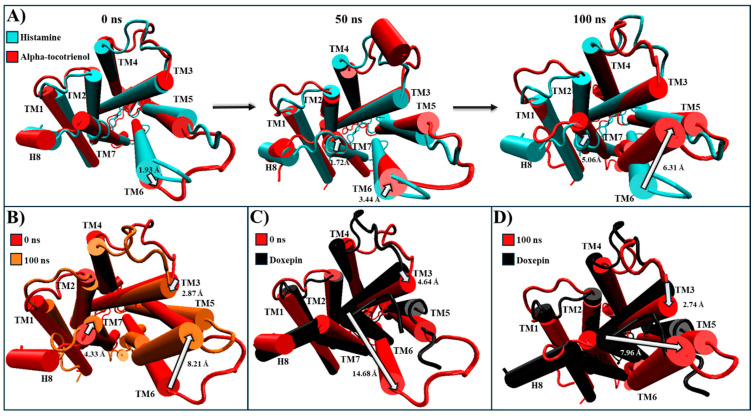
Structural comparison of HR1 (intracellular views): (**A**) alpha-tocotrienol complex (red) dynamics at 0, 50, and 100 ns compared to histamine experimental structure (cyan). (**B**) Alpha-tocotrienol complex at 0 ns (red) and 100 ns (orange). (**C**) Alpha-tocotrienol complex at 0 ns (red) compared to doxepin experimental structure (black). (**D**) Alpha-tocotrienol complex at 100 ns (red) compared to doxepin experimental structure (black). White arrows illustrate the measurements between TMs. Measurements were performed with VMD based on the alpha carbon of TM3’s residue 130, TM6’s residue 408, and TM7’s residue 472.

For HR1, only alpha-tocotrienol was chosen due to its significantly higher affinity. The experimental structures of HR1–histamine (PDB: 7DFL) and HR1–doxepin (PDB: 3RZE) were used for comparison [37,56]. In this way, despite the alpha-tocotrienol/HR1 complex being based on the HR1–histamine complex, the most populated cluster revealed an intracellular closure of the G protein cavity (Table 4 and Figure S3). At the simulation’s start, the alpha-tocotrienol complex’s intracellular segment had TM6 positioned 1.93 Å farther from the structural core compared to the histamine complex (Figure 6A). By 50 ns, TM6 had undergone a displacement of 3.44 Å towards the core, directed towards TM3 and TM5. At 100 ns, TM6 was positioned 6.31 Å closer to TM5/TM3 than the histamine complex (Figure 6A). In this way, as depicted in Figure 6B and Movie S1, TM6 exhibited a consistent trend, progressively shifting towards TM3 and TM5. This movement of TM6 brought it to the same position as the doxepin structure, effectively closing the G protein cavity (Figure 6C,D). This motion of TM6 originated from an extracellular stimulus exerted by alpha-tocotrienol in the same direction as the inhibitor (Figure S4). Consequently, despite its primary interactions with TM3, TM4, and TM5, alpha-tocotrienol effectively sealed the intracellular cavity of the G protein to a degree nearly matching that of doxepin (Movie S1).

**Figure 7:**
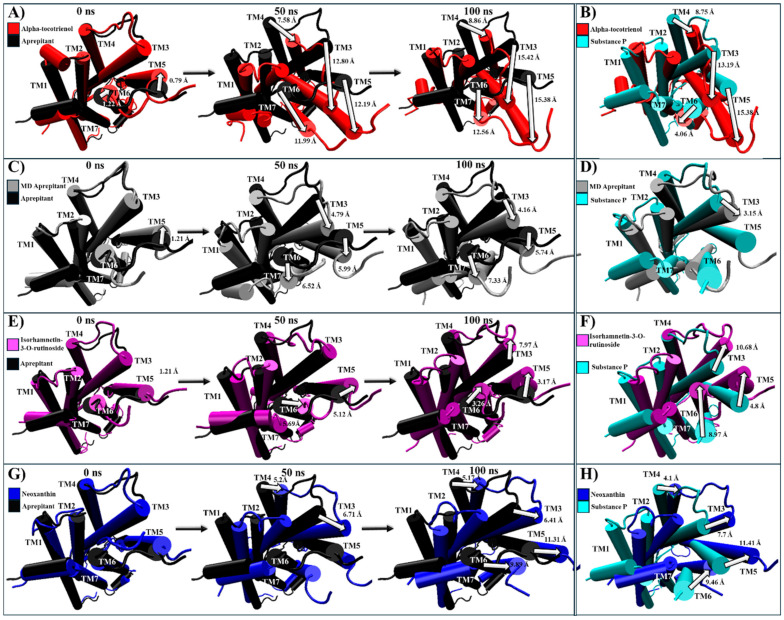
Structural comparison of NKR1 (intracellular view). (**A**) Alpha-tocotrienol complex dynamics (red) at 0, 50, and 100 ns compared to experimental structure of NKR1/aprepitant (black). (**B**) Alpha-tocotrienol complex (red) compared to experimental structure of NKR1/substance P (cyan). (**C**) MD aprepitant complex dynamics (silver) at 0, 50, and 100 ns compared to experimental structure of NKR1/aprepitant (black). (**D**) MD aprepitant complex (silver) compared to experimental structure of NKR1/substance P (cyan). (**E**) Isorhamnetin-3-O-rutinoside complex dynamics (purple) at 0, 50, and 100 ns compared to experimental structure of NKR1/aprepitant (black). (**F**) Isorhamnetin-3-O-rutinoside complex compared to experimental structure of NKR1/substance P (cyan). (**G**) Neoxanthin Complex dynamics (blue) at 0, 50, and 100 ns compared to experimental structure of NKR1/aprepitant (black). (**H**) Neoxanthin complex compared to experimental structure of NKR1/substance P (Cyan). Measurements were performed with VMD based on the alpha carbon of TM3’s residue 135, TM4’s residue 144, TM5’s residue 224, and TM6’s residue 242. White arrows illustrate the measurements between TMs.

For NKR1, isorhamnetin-3-O-rutinoside, alpha-tocotrienol, and neoxanthin exhibited higher binding affinities than aprepitant. Thus, these complexes were compared to the experimental structures of NKR1–substance P (PDB:7RMG), NKR1–aprepitant (PDB:6J20), and the MD structure of NKR1–aprepitant [57,63]. Encouragingly, the most populated cluster of NKR1–aprepitant maintained the same intracellular closing pattern that was observed in the experimental structure (Figure S5). Neoxanthin and isorhamnetin-3-O-rutinoside closed the G protein cavity like aprepitant (Table 4 and Figure S5). At the same time, alpha-tocotrienol induced a complete remodeling of the G protein binding site by notably shifting TM6 close to TM3 and TM5 (Table 4 and Figure S5). Thus, all three phytochemicals were further analyzed. Alpha-tocotrienol’s remodeling increased proportionally with time (Figure 7A and Movie S2A). At 50 ns, alpha-tocotrienol moves TM3, TM5, and TM6 more than 10 Å in the opposite direction of TM2 and TM4. Furthermore, at 100 ns, these four TMs continued moving in the same direction, drawing closer than any other TMs within all the NKR1 complexes (Figure 7A). Thus, alpha-tocotrienol completely reshaped the NKR1 structure in both the intra- and extracellular portions (Figure S6). The MD structure of NKR1–aprepitant exhibited a similar structural shift from alpha-tocotrienol, but with a lower intensity (Figure 7C,D). Finally, isorhamnetin-3-O-rutinoside did not display any consistent or specific shift pattern (Figure 7E,F), while neoxanthin consistently moved TM3, TM5, and TM6 towards the direction of the initial location of TM5 (see Figure 7G,H). Therefore, all three phytochemicals effectively preserved the closure of the intracellular cavity of G proteins (Movie S2).

**Figure 8:**
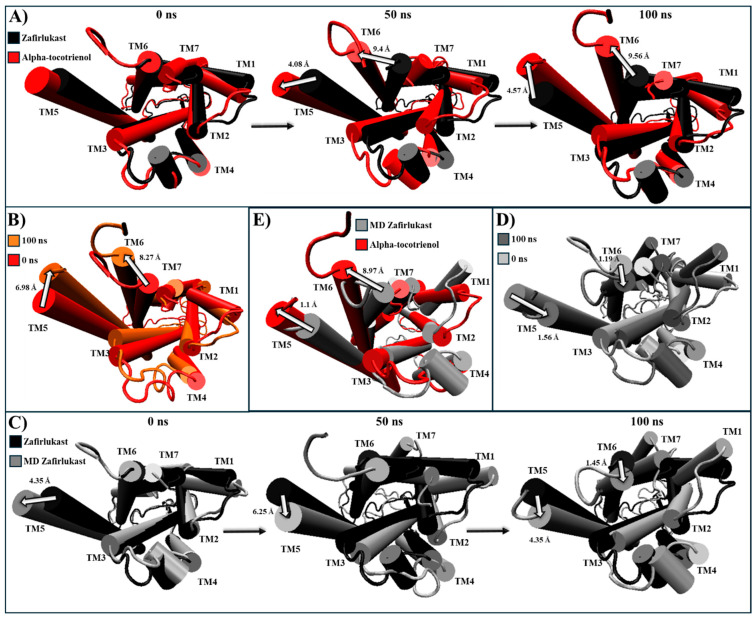
Structural comparison of CLR1 (intracellular view). (**A**) Alpha-tocotrienol complex dynamics (red) at 0, 50, and 100 ns compared to experimental structure of CLR1/zafirlukast (black). (**B**) Alpha-tocotrienol complex at 0 ns (red) and 100 ns (orange). (**C**) MD zafirlukast complex dynamics (silver) at 0, 50, and 100 ns compared to experimental structure of CLR1/zafirlukast (black). (**D**) Zafirlukast complex at 0 ns (silver) and 100 ns (grey). (**E**) Alpha-tocotrienol complex (red) compared to MD zafirlukast at 100 ns (silver). Measurements were performed with VMD based on the alpha carbon of TM5’s residue 221 and TM6’s residue 225. White arrows illustrate the measurements between TMs.

For CLR1, only alpha-tocotrienol and neoxanthin showcased superior affinity compared to the known inhibitor, zafirlukast. Given the absence of an experimental CLR1 structure with an agonist, our comparison relied on structural deviations of the reference structure (PDB:6RZ5) compared to the MD CLR1/zafirlukast conformation identified through a cluster analysis. Our simulations revealed that zafirlukast prompted TM6 to shift towards the center, effectively closing the cavity within TM3, TM5, TM6, and TM7 (Table 4 and Figure S7). Alpha-tocotrienol induced a subtle inward movement of TM2 and TM7 towards the core, whereas neoxanthin caused TM6 to move outward from the core, as an agonist would (Table 4 and Figure S7). Considering that such outward movement may be undesirable, we focused our subsequent analyses solely on alpha-tocotrienol. Throughout the 100 ns simulation, alpha-tocotrienol and zafirlukast maintained a similar conformation, with noticeable movements primarily observed in TM5 and TM6 (Figure 8 and Figure S8). Despite these movements, none of the movement followed a specific direction (Movie S3). However, it is worth noting that alpha-tocotrienol generated a movement of TM6 away from the center. While this movement might not be ideal for obstructing the cavity for G proteins, it is important to mention that TM6 did not follow a particular trajectory either in the extracellular portion or intracellular portion (Figure 8 and Figure S8). Within the intracellular portion, from 0 to 50 ns, it moved away from TM7, while from 50 to 100 ns, it moved closer (Figure 8A). Additionally, the alpha-tocotrienol-induced movements did not generate an opening as pronounced as that of neoxanthin. Therefore, we associated this movement with the unidirectional movements observed in other TMs within HR1 and NKLR1 (Figure 6 and Figure 7). Thus, alpha-tocotrienol maintained the inactivation state of CLR1.

**Figure 9:**
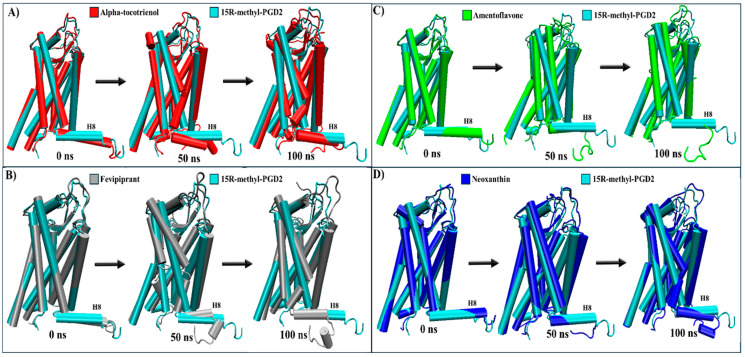
Structural comparison of CRTH2 (intracellular view). (**A**) Alpha-tocotrienol complex dynamics (red) at 0, 50, and 100 ns compared to experimental structure of CRTH2/15R-methyl-PGD2 (cyan). (**B**) Fevipiprant complex dynamics (silver) at 0, 50, and 100 ns compared to experimental structure of CRTH2/15R-methyl-PGD2 (cyan). (**C**) Amentoflavone complex dynamics (green) at 0, 50, and 100 ns compared to experimental structure of CRTH2/15R-methyl-PGD2 (cyan). (**D**) neoxanthin complex dynamics (blue) at 0, 50, and 100 ns compared to experimental structure of CRTH2/15R-methyl-PGD2 (cyan).

For CRTH2, amentoflavone, alpha-tocotrienol, and neoxanthin exhibited a stronger binding affinity to the receptor’s pocket than the established inhibitor fevipiprant. A structural analysis was conducted to explore these interactions by employing the agonist reference structure (PDB:7M8W) and the CRTH2/fevipiprant complex (PDB:6D26). As mentioned above, the only identified structural change was the agonist-induced compaction of helix 8. [61,62]. During the MD simulation, most transmembrane segments remained unaltered. The only distinctive behavior was the decompaction of helix 8, which lost its helical structure to some degree in all complexes (Table 4 and Figure S9). A further analysis showed that helix 8 lost its helical structure in all systems within the first 50 ns (Figure 9 and Movie S4). Therefore, none of these systems should inhibit arrestin recruitment.

**Figure 10:**
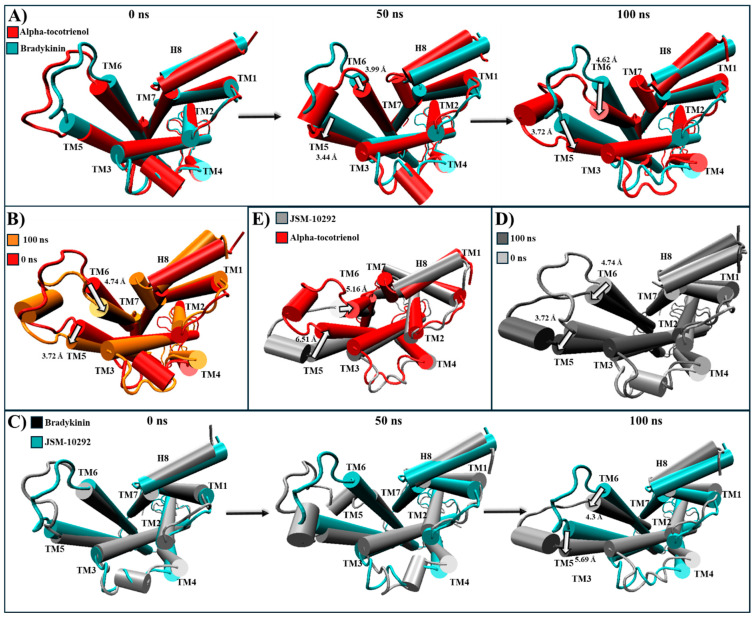
Structural comparison of BK2R (intracellular view). (**A**) Alpha-tocotrienol complex dynamics (red) at 0, 50, and 100 ns compared to experimental structure of BK2R/bradykinin (cyan) (**B**) Alpha-tocotrienol complex at 0 ns (red) and 100 ns (orange). (**C**) JSM-10292 complex dynamics (silver) at 0, 50, and 100 ns compared to experimental structure of BK2R/bradykinin (cyan). (**D**) JSM-10292 complex at 0 ns (silver) and 100 ns (grey). (**E**) Alpha-tocotrienol complex (red) compared to JSM-10292 at 100 ns (silver). Measurements were performed with VMD based on the alpha carbon of TM5’s residue 253 and TM6’s residue 266. White arrows illustrate the measurements between TMs.

All phytochemicals exhibited a superior affinity for BK2R compared to the established inhibitor. A structural comparison was conducted between the most populated clustering structures and PDB:7F6H, the base structure for the simulation, in which, BK2R is bound to a G protein and the agonist, bradykinin [64]. During the 100 ns simulation, amentoflavone and neoxanthin were not able to induce any significant changes in the conformational structure (Figure S10). However, JSM-10292 and alpha-tocotrienol triggered a displacement of TM6 and TM7 towards the core of the transmembrane helices (Figure S10). Conversely, isorhamnetin-3-O-rutinoside caused TM6 to move away from the core, surpassing even the activated position (Figure S10). Given that amentoflavone, neoxanthin, and isorhamnetin-3-O-rutinoside did not induce shifts similar to those caused by the known inhibitor, they were not further analyzed. Alpha-tocotrienol and JSM-10292 prompted movements of TM5 and TM6 (Figure 10 and Movie S5), with a discernible inactivation movement of TM6 towards the core being detected. Throughout the MD simulation, alpha-tocotrienol moved TM6 4.74 Å towards the direction of TM3, effectively closing the G protein cavity (Movie S5A and Figure 10B). Consequently, alpha-tocotrienol demonstrated the ability to act as a BK2R antagonist.

### 2.6. UD Phytochemicals’ Effects on Sensorial Nerves and Immune Cells

The investigation into botanical remedies for AR has spotlighted UD since the early 1990s [14]. In a recent clinical trial, Bakhshaee et al. explored the impact of a one-month UD treatment, which showed a significant reduction in the severity of clinical symptoms as evaluated through the SNOT-22 (Sinonasal Outcome Test-22). This widely recognized questionnaire is specifically designed to comprehensively assess the health-related quality of life in individuals with sinonasal conditions. Despite these positive findings, uncertainties persist, particularly in relation to UD’s limited impact on modifying IgE levels [15].

IgE, released promptly after allergen exposure, initiates the early phase response characterized by histamine release, sneezing, rhinorrhea, and nasal congestion [65]. While UD may not alter IgE release, it holds the potential for improving AR symptoms by potentially inhibiting the subsequent reactions in this cascade. This is especially remarkable in this context, as GPCRs are key to the activation of sensorial nerves [66,67,68,69,70]. The activation of these receptors could lead to pain, itch, cough, and neurogenic inflammation [71].

**Figure 11:**
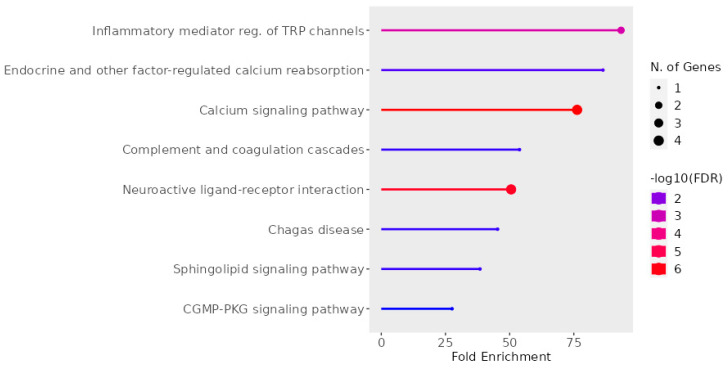
Pathways associated with AR targets. The graph was made using Shinny GO 8.0 Analysis [74].

From Figure 11, it is apparent that the coordinated activity of five receptors significantly impacts the regulation of the inflammatory response through transient receptor potential (TRP) channels and calcium signaling. Within the realms of pain and itch pathways, the activation of GPCRs activates a signaling cascade that culminates with TRP activation. This occurs through the activation of phospholipases or via the stimulation of kinases, which positively modulate TRP activity [72]. Subsequently, TRPs induce a calcium influx, which modulates the activation of T cells and mast cells and promotes mucosal secretion from epithelial cells [73]. Within sensory neurons, TRP activation stimulates depolarization, giving rise to sensations of pain or itch [72]. Therefore, TRPs serve as pivotal regulators of AR [73].

Additionally, there are specific instances where multiple GPCRs can activate the same TRP channel. For example, HR1, NKR1, and BK2R have been shown to activate transient receptor potential vanilloid 1 (TRPV1) [75,76,77]. TRPV1 has been implicated in various physiological responses, including rhinorrhea, itch, sneezing, and T-cell activation [78,79,80]. Furthermore, mice lacking TRPV1 exhibited reduced IgE-mediated anaphylaxis [81]. Thus, even though the same GPCRs may not be expressed in the same cell types, this signaling pathway can be inhibited through multiple avenues, highlighting the significance of targeting this pathway as a therapeutic approach.

Moreover, the simultaneous inhibition of the five GPCRs studied here can positively impact a broader range of symptoms. HR1 antagonists effectively address rhinorrhea, sneezing, and nasal itching [82]. Meanwhile, HR1 and BK2R both increase vascular permeability. Additionally, HR1, alongside BK2R, contributes to increased vascular permeability [83,84]. Furthermore, NKR1 antagonists alleviate similar symptoms and reduce allergy-induced ocular redness and eosinophilic infiltration [29,85,86], benefits that traditional antihistamines fail to achieve. Finally, CLR1 as an NKR1 antagonist also influences eosinophilic proliferation and accumulation and type 2 innate lymphoid cell activity, both of which are strongly linked to rhinosinusitis [87,88]. CRTH2 also controls type 2 innate lymphoid cell activity [89]. In this way, these multiple targets broaden the modulation of more key AR players.

### 2.7. Perspective

Among the UD phytochemicals, alpha-tocotrienol has been the only molecule studied within this context. This is especially due to its observed capability to induce an antagonist shift in various structures. Alpha-tocotrienol holds particular importance as the primary form of vitamin E in blood and tissues [90]. Interestingly, a lower serum concentration of vitamin E has been linked to AR in children and pregnant women [91,92]. Moreover, treatment with vitamin E has demonstrated efficacy in mitigating inflammation and mucus secretion [93]. Despite this, the effectiveness of vitamin E treatment in AR remains uncertain, with studies yielding inconclusive results [93,94].

However, exploring beyond traditional vitamin E, long-chain metabolites have shown promise for inhibiting key aspects of allergic responses, including the Th2 immune response and mast cell activation [95]. This aligns with the desired inhibition of targets for AR. Intriguingly, in a rat model of AR, the intranasal administration of a tocotrienol-rich fraction demonstrated potential in reducing vascular congestion, inflammation, and polymorphonuclear cell infiltration [96]. Additionally, it is worth noting that within the extract utilized by Roschek Jr. et al. against known proteins associated with AR, including HR1, alpha-tocotrienol was identified with a relative abundance of 8.65%, which is encouraging [16]. Despite these promising findings, it is essential to highlight the scarcity of specific studies focusing on alpha-tocotrienol’s effects on AR or its direct impact on inflammation.

Conversely, amentoflavone, neoxanthin, and isorhamnetin 3-O-rutinoside have not been thoroughly studied in the context of AR. Notably, another extract (*S. uncinata*), in which amentoflavone is recognized as the principal flavonoid, demonstrated positive effects against asthma. This effect was attributed to its interaction with bitter taste receptors (T2Rs), a subfamily of GPCRs [97]. Additionally, amentoflavone exhibits anti-neuroinflammatory properties by inhibiting the TLR4/MyD88/NF-κB pathway and acting as a matrix metalloproteinase two inhibitor, which are other known AR targets [98,99].

Therefore, it is encouraging to note that our top candidate, alpha-tocotrienol, is supported by the existing literature. Furthermore, the positive outcomes demonstrated by long-chain metabolites in alleviating AR symptoms serve to reinforce our results [95]. In addition, it might highlight that molecular size plays a significant role in this inhibition. In our study, this was evidenced by the fact that both alpha-tocotrienol and neoxanthin possess long-chain structures. Nevertheless, while this study provides valuable insights into the potential therapeutic effects of UD phytochemicals in AR management, some limitations should be acknowledged.

Firstly, molecular dynamics simulations offer valuable structural insights but further validation through in vitro assays and animal studies is essential to assess the efficacy and safety of these phytochemicals in AR treatment. By addressing these limitations, our understanding of UD’s therapeutic effects on AR could pave the way for the development of novel and effective treatment strategies. In particular, alpha-tocotrienol shows potential as a candidate for managing AR symptoms and may even serve as a scaffold for designing molecules targeting multiple AR pathways.

## 3. Materials and Methods

### 3.1. Urtica dioica Phytochemicals

To establish a comprehensive database of UD phytochemicals, we meticulously curated literature reports from 2008 to 2021. This database, comprising 277 phytochemicals, was compiled from phytochemical screening studies and a review. However, it is important to note that these studies exhibited notable variations in plant growth conditions and extraction techniques, factors that significantly influence screening outcomes. For instance, Ilies et al. utilized plants collected in June from the Dambovita region, employing hydrodistillation for extraction [100], whereas Gül et al. collected plants in September in Tortepe, Turkey, using the same extraction method [101]. Repajić et al. collected plants before, during, and after flowering in various regions of Croatia, utilizing an Accelerated Solvent Extractor [47]. Roschek Jr. et al. and Ayers et al. obtained plant material from Blessed Herbs and extracted phytochemicals using HPLC-grade water [16,102]. Pinelli et al. cultivated UD under controlled conditions in Tuscany and extracted phytochemicals using EtOH [48].

Similarly, García et al. collected plants in July in Tuscany and employed a sonicator for extraction [49]. Al-Tameme et al. utilized methanol for extraction [66], while Grauso et al. collected plants in April and July in Cicerale, Italy, and used n-hexane for extraction [103]. Moreover, Majedi et al. and Grauso et al. compiled data from UD phytochemicals obtained from different sources [50,104]. This enhanced database consolidates a wide array of phytochemical data and offers a more nuanced understanding of the variability in extraction methods and plant growth conditions across different studies. By incorporating the diverse methodologies employed by various researchers, it provides a more comprehensive view of the phytochemical composition of UD. Subsequently, the database was refined to include only phytochemicals with available structures in PubChem [105], which is essential for docking and MD analyses. These phytochemicals are listed in Table S17.

### 3.2. Molecular Docking

To yield a more reliable result, the UD database was analyzed for docking to HR1, NKR1, CLR1, CRTH2, and BK2R with two software products, MOE [106] and AutoDock Vina [107]. The structure and detailed information about the system characteristics of all five receptors is provided in Table S18. Receptor structures were prepared using MOE for the MOE docking and AutoDock Tools [108] for the AutoDock Vina docking. All docking pockets were based on the reported binding pocket of the antagonist/agonist. In HR1, the MOE docking pocket was set as residues Asp107, Tyr108, Ile115, Trp158, Lys179, Phe184, Phe190, Lys191, Thr194, Asn198, Phe199, Phe424, Trp428, Tyr431, Phe432, His450, and Tyr458, whereas the grid box of AutoDock Vina was set as 134 × 135 × 161 Å, with a size of 29.25 × 29.25 × 25.5 Å. For NKR1, the MOE pocket was set as residues Asn109, Pro112, Ile113, Val116, Gln165, Ile182, Trp184, Glu193, His197, Val200, Ile204, Trp261, Phe264, His265, and Phe268, and the grid box of AutoDock Vina was set as −86.928 × 62.428 × 366.378, with a size of 25.5 × 25.5 × 27.0 Å. For CLR1, the MOE pocket was set as the residues Arg79, Thr100, Tyr104, Tyr108, Phe150, Thr154, Ser155, Pro157, Phe158, Phe174, Pro176, Val186, Leu189, His190, Ser193, Val196, Gly197, Tyr249, Arg253, His256, Leu257, Leu260, Val277, Thr280, and Leu281, and the Autodock Vina grid box was set as 12.575 × 12.957 × −13.8 Å, with a size of 27.75 × 30.75 × 32.25 Å. For CRTH2, the MOE pocket was set as residues Phe87, Phe90, Trp97, His107, Phe111, Arg179, Cys182, Tyr183, Tyr184, Lys210 Tyr262, Leu286, Pro287, and Phe294, and the grid box of Autodock Vina was set as −4.802 × −56.984 × 544.436 Å, with a size of 28.5 × 24.75 × 30.0 Å. Finally, for BK2R, the MOE pocket was set as residues Trp113, Phe121, Asn134, Ile137, Ser138, Leu141, Ser190, Met192, Ala210, Cys211, Val212, Ile213, Asn225, leu228, Phe286, Gln287, Thr290, Asp293, Glu307, Ile310, Asp311, and Thr314 Å, and the Autodock Vina grid box set as 120.628 × 129.497 × 104.933, with a size of 27.75 × 24.0 × 30.75 Å. We conducted a thorough validation process to bolster the robustness of our docking methodology. This encompassed the re-docking of co-crystallized ligands tailored to each receptor, utilizing both software platforms. Remarkably, our analysis unveiled a consistent localization of ligands within the anticipated binding pockets, with minimal variance in conformations (Figures S12 and S13). All these structures RMSD’s were calculated using PYMOL v 2.3.4 [109] (Figure S14).

### 3.3. MD Positive and Negative Controls

For the MD positive control, known inhibitors were utilized. As a negative control, we opted for a widely used drug devoid of any capacity to alleviate AR symptoms or exhibit anti-inflammatory effects. The negative control was identified through the Chemical Structure Search on DrugBank with a threshold of 0.5, within the dataset “Approved Drugs” [110], using amentoflavone as the reference with the highest affinity score among the four phytochemicals. The selected molecule, flavoxate, shared a score of 0.552 with amentoflavone and is commonly employed in treating overactive bladder syndrome [111]. Flavoxate has not been reported to possess direct anti-inflammatory effects, nor does it influence prostaglandin synthesis [112].

### 3.4. MD Simulation

The MD simulation employed the optimal conformation achieved by molecules in MOE or AutoDock Vina docking. Receptor–ligand complexes were constructed using the Amber22 package’s antechamber and tleap modules [113]. Ligand force fields were parameterized with the general amber force field [114] and the AM1-BCC method [115], while proteins were built using the amber ff14SB force field [116]. Counter ions were added to neutralize complexes and equilibrate the total charge, and systems were immersed in a truncated octahedral water box with a 12 Å boundary [117]. System equilibration involved 5000 cycles using the steepest descent method followed by 4000 cycles using the conjugate gradient method. The temperature was increased over 200 ps from to 310 K under an NVT ensemble, with heavy atoms in the receptor constrained using an elastic constant of 3 kcal/mol∙Å^2. Density equilibrium was achieved over 200 ps under an NTP ensemble, and pressure equilibration for 600 ps at 310 K with constrained heavy atoms. MD simulations, conducted under an NPT ensemble without restrictions, employed the Particle Mesh Ewald (PME) algorithm [118] for treating long-range electrostatic interactions [119]. Van der Waals and short-range electrostatic interactions were set to 10 Å. A time step of 2 fs was used, and the SHAKE algorithm [120] was used to constrain the bond lengths between hydrogen atoms and linked heavy atoms. The MD simulations were run in triplicate using the pmemd.cuda module in Amber22 [113]. The trajectory analysis utilized a single concatenated trajectory from overlapping triplicate simulations, providing estimates for the root mean squared deviation (RMSD), radius of gyration (RG), clustering analysis, and binding free energy calculations. The representative conformation shown in the figures corresponds to the most populated conformation obtained through a clustering analysis performed over the equilibrated simulation time of each system. The structural comparisons involved superimposing and aligning structures using PYMOL v 2.3.4 [109] and visualized in VMD software [121]. Two-dimensional graphical interactions were computed with MOE, and the legend of the interaction can be seen within each figure.

### 3.5. Binding Free Energy Studies with MMGBSA and MMPBSA

The binding free energy values were estimated over the equilibrated simulation time from the single concatenated trajectories using the MMGBSA method using the single-method MD simulation protocol with the MMPBSA.py module [122]. These methods were used to obtain the binding free energy values and perform the per-residue decomposition analysis as previously described [123].

## 4. Conclusions

Based on the docking analysis, this study showed that there are phytochemicals from UD that possess a higher or similar affinity for HR1, NKR1, CLR1, CRTH2, and BK2R than known inhibitors. The docking analysis revealed that amentoflavone, alpha-tocotrienol, neoxanthin, and isorhamnetin 3-O-rutinoside possess a high affinity for these receptors. The MD simulation confirmed this observation by showing that all the phytochemicals generated a higher or similar binding affinity towards all the receptors, except for neoxanthin with HR1. Out of these four phytochemicals, alpha-tocotrienol stands out as it generated a high affinity in all the systems while also causing structural changes that are associated with the structural inactivation of the five GPCRs. This result is in accordance with the reports of vitamin E and tocotrienols having an effect on AR symptoms. Thus, these results suggest that amentoflavone, alpha-tocotrienol, neoxanthin, and isorhamnetin 3-O-rutinoside might alleviate AR symptoms by inhibiting HR1, NKR1, CLR1, CRTH2, and BK2R. Although further research using in vitro and in vivo analyses is needed, our results suggest that alpha-tocotrienol can act as a multi-target inhibitor and thereby treat multiple AR symptoms.

## Figures and Tables

**Table 1:** The affinity values from the best 15 molecules from software and the number of appearances in the top 15.

Phytochemical Name	HR1	Nkr1	CLR	CRTH2	BK2R	Mean Value	Top 15 Appearances
Amentoflavone	−6.362	**−9.604**	**−9.565**	**−8.880**	**−8.384**	−8.559	4
Alpha-tocotrienol	**−7.310**	**−8.402**	**−9.108**	**−9.258**	−6.959	−8.207	4
Neoxanthin	**−6.856**	**−7.964**	**−8.872**	**−9.529**	−7.184	−8.081	4
7α-Hydroxy-sitosterol	**−7.465**	**−7.963**	−7.939	−8.445	**−8.569**	−8.076	3
Isorhamnetin 3-O-rutinoside	−6.138	**−8.519**	**−8.373**	**−8.698**	**−8.181**	−7.982	4
_γ_-Sitosterol	**−7.337**	−7.738	−7.603	−8.356	**−8.603**	−7.927	2
Cholecalciferol	**−7.294**	−7.647	−8.180	**−8.705**	**−7.596**	−7.884	3
Kaempferol-3-rutinoside	−5.993	**−8.404**	**−8.418**	−8.147	**−8.278**	−7.848	3
Isorhamnetin rutinoside	−6.069	−7.814	−7.916	**−8.620**	**−8.718**	−7.828	2
Epigallocatechin gallate	−6.431	−7.826	**−8.923**	−8.355	−7.516	−7.810	1
Hecogenin	**−7.091**	**−7.953**	−8.059	**−8.852**	−6.849	−7.761	3
Solanidine	**−7.234**	−7.349	−7.705	**−8.703**	**−7.781**	−7.755	3
7β-Hydroxy-sitosterol	−5.974	−7.701	−7.867	−8.482	**−8.645**	−7.734	1
Dicaffeoylquinic acid	−6.519	**−8.128**	−8.253	−8.511	−7.205	−7.723	1
Epicatechin gallate	−6.458	−7.329	**−8.938**	**−8.610**	−7.180	−7.703	2
Known inhibitor	−7.311	−9.039	−11.292	−9.207	−8.375	−9.045	-

The residues in bold describe the phytochemicals’ presence in each system’s top 15 affinity values. Values are in kcal/mol.

**Table 2:** MMGBSA calculations for the binding affinity of phytochemical–receptors complexes.

Receptor	Alpha-Tocotrienol	Amentoflavone	Isorhamnetin 3-O-Rutinoside	Neoxanthin	Known Inhibitor
HR1	−38.493 (±5.11)	−28.679 (±3.30)	−26.446 (±4.21)	-	-
NKR1	−45.400 (±3.28)	−33.954 (±3.70)	−46.756 (±4.78)	−39.455 (±7.95)	−38.343 (±3.61)
CLR1	−54.785 (±2.89)	−36.620 (±3.39)	−41.335 (±3.13)	−61.436 (±5.20)	−49.684 (±4.43)
CRTH2	−53.334 (±4.96)	−45.005 (±3.37)	−41.325 (±6.32)	−56.836 (±4.22)	−43.221 (±4.58)
BK2R	−42.654 (±3.71)	−38.984 (±5.37)	−37.080 (±4.76)	−47.394 (±4.81)	−32.310 (±4.73)

Values are in kcal/mol.

**Table 3:** MMPBSA calculations for the binding affinity of alpha-tocotrienol and the known inhibitors.

Receptor	Alpha-Tocotrienol	Known Inhibitor
HR1	−34.65 (±4.70)	-
NKR1	−35.7 (±4.65)	−29.52 (±5.01)
CLR1	−45.55 (±3.07)	−38.95 (±4.65)
CRTH2	−31.73 (±5.38)	−52.55 (±5.93)
BK2R	−31.93 (±3.71)	−32.310 (±4.73)

Values are in kcal/mol.

**Table 4:** Preliminary comparative analysis of receptor inactivation and ligand-induced conformational changes.

Receptor	Reported Inactivation	Ligand	Conformational Change Observed
HR1	Extracellular: Antagonist pushes TM3, TM6, and TM7 away from its core [56]. Intracellular: Moves TM6 towards TM2, TM3, and TM7, which closes the G protein intracellular cavity [56].	Alpha-tocotrienol	Extracellular: Doxepin pushes TM3 and TM6 away to a degree almost comparable to that of doxepin (Figure S3). Intracellular: Bring TM3 and TM6, as well as TM2 and TM7, together, which closed the G protein intracellular cavity (Figure S3).
NKR1	Extracellular: The antagonist prompts TM5, TM6, and TM7 to move away from the center [57,63]. Intracellular: Closure of the G protein cavity between TM6, TM7, and TM5 [57,63].	Alpha-tocotrienol	Extracellular: TM2 and TM7 become closer to the core’s perimeter, while TM6 moves in the direction of TM7 (Figure S5). Intracellular: Moves TM2 and TM7 inward, while also bringing TM3, TM5, and TM6 remarkably close (Figure S5).
		Isorhamnetin-3-O-rutinoside	Extracellular: Moves TM6 and TM5 away from the core, while TM7 moves close to TM2 (Figure S5). Intracellular: Moves TM2, TM6, and TM7 inward (Figure S5).
		Neoxanthin	
CLR1	Extracellular: Zafirlukast induces TM3, TM4, TM5, and TM7 to move away from the center (Figure S7). Intracellular: G proteins bind within TM3, TM5, TM6, and TM7 [60]. Zafirlukast shifts TM6 towards the center, effectively closing the cavity (Figure S7).	Alpha-tocotrienol	Extracellular: Slightly moves MT2, TM4, and TM7 away from the core (Figure S7) Intracellular: Induces an inward movement of TM2 and TM7 while slightly separating TM6 and TM7 (Figure S7).
		Neoxanthin	Extracellular: Moves TM6 towards the core (Figure S7) Intracellular: Induces an outward movement of TM5, TM6, and TM7, comparable to an agonist-induced movement (Figure S7).
CRTH2	Intracellular: The only known structural change is that the agonist causes helix 8 to undergo compaction, inhibiting arrestin recruitment [61,62].	Alpha-tocotrienol	Intracellular: Leads to the loosening of helix 8 around Leu323 (Figure S9).
		Amentoflavone	Intracellular: Results in the relaxation of helix 8 due to a loosening of the structure in Leu327, like the inhibitor (Figure S9).
		Neoxanthin	Intracellular: Causes the loosening of helix 8 around Leu323 (Figure S9).
BK2R	Extracellular: TM6 moves outside the center of the core [64]. Intracellular: TM6 moves inward to avoid the generation of the BK2R-Gq complex within TM2, TM3, TM5, and TM7 [64].	Alpha-tocotrienol	Extracellular: A slight movement of TM6 away from the core (Figure S10). Intracellular: Generates a movement of TM6 towards TM3, while TM7 moves towards TM6 (Figure S10).
		Amentoflavone	No significant movement was observed.
		Isorhamnetin-3-O-rutinoside	Extracellular: A slight movement of TM6 towards TM5 (Figure S10). Intracellular: Movement of TM6 and TM5 outside the core (Figure S10).
		Neoxanthin	No significant movement was observed.

This table presents the reported conformational changes essential for the inactivation of HR1, NKR1, CLR1, CRTH2, and BK2R when interacting with antagonists. Additionally, it highlights the conformational changes observed within the most populated cluster during the MD simulations with the phytochemicals.

## Data Availability

All data and supporting information are available in the article.

## Supplementary Materials

The following supporting information can be downloaded at: https://www.mdpi.com/article/10.3390/molecules29081765/s1, Table S1: UD phytochemicals’ docking values for HR1; Table S2: UD phytochemicals’ docking values for NKR1; Table S3: UD phytochemicals’ docking values for CLR1; Table S4: UD phytochemicals’ docking values for CRTH2; Table S5: Phytochemicals’ docking values for BK2R; Table S6: The affinity values and residues of HR1 that interact with fexofenadine and the best 15 molecules from both software; Table S7: The affinity values and residues of NKR1 that interacted with Aprepitant and the best 15 molecules, with both software. Table S8: The affinity values and residues of CLR1 that interact with Zafirlukast and the best 15 molecules from both software; Table S9: The affinity values and residues of CRTH2 that interact with Fevipiprant and the best 15 molecules from both software; Table S10: The affinity values and residues of BK2R that interact with JSM10292 and the best 15 molecules from both software; Table S11: UD phytochemicals’ mean values for the five receptors; Table S12: Main values from the HR1 per-residue decomposition of the free energy; Table S13: The main values from the NKR1 per-residue decomposition of the free energy; Table S14: Main values from the CLR1 per-residue decomposition of the free energy; Table S15: Main values from the CRTH2 per-residue decomposition of the free energy; Table S16: Main values from the BK2R per-residue decomposition of the free energy; Figure S1: RMSD of (A) HR1, (B) NKR1, (C) CLR1, (D) CRTH2, (E) BK2R; Rg of (F) HR1, (G) NKR1, (H) CLR1, (I) CRTH2, (J) BK2R; Figure S2: RMSF of (A) HR1, (B) NKR1, (C) CLR1, (D) CRTH2, (E) BK2R; Figure S3: Structural comparison between HR1–alpha-tocotrienol complex (green), HR1–histamine (red), and HR1–doxepin (blue); intracellular view of (A) the three structures, (B) HR1–histamine and HR1–doxepin, (C) HR1–histamine and HR1–alpha-tocotrienol, (D) HR1–alpha-tocotrienol and HR1–doxepin; extracellular view of (E) the three structures, (F) HR1–histamine and HR1–doxepin, (G) HR1–histamine and HR1–alpha-tocotrienol, (H) HR1-alpha-tocotrienol and HR1–doxepin; Figure S4: Structural comparison (extracellular view) of (A) alpha-tocotrienol complex (red) within 0, 50, and 100 ns and histamine experimental structure (cyan), (B) alpha-tocotrienol complex at 0 ns (red) and 100 ns (orange), (C) alpha-tocotrienol complex at 0 ns (red), and doxepin experimental structure (black), (D) alpha-tocotrienol complex at 100 ns (red), and doxepin experimental structure (black); Figure S5: Structural comparison between structures; extracellular view of (A) all structures, (B) NKR1–substance P (turquoise) and MD NKR1–aprepitant (black), (C) MD NKR1–aprepitant and experimental NKR1–aprepitant (silver), and NKR1–substance P with (D) NKR1–alpha-tocotrienol (red), (E) NKR1–isorhamnetin 3-O-rutinoside (purple), and (F) NKR1–neoxanthin (blue); intracellular view of (G) all structures, (H) NKR1–substance P and MD NKR1–aprepitant, (I) MD NKR1–aprepitant and experimental NKR1–aprepitant, and NKR1–substance P with (J) NKR1–alpha-tocotrienol, (K) NKR1–isorhamnetin 3-O-rutinoside, and (M) NKR1–neoxanthin; Figure S6: NKR1 structural comparison (extracellular view) of (A) alpha-tocotrienol complex (red) within 0, 50, and 100 ns and experimental structure of NKR1/aprepitant (black), (B) alpha-tocotrienol complex and the experimental structure of NKR1/substance P (Cyan), (C) MD aprepitant complex (red) within 0, 50, and 100 ns and experimental structure of NKR1/aprepitant (black), (D) MD aprepitant complex and the experimental structure of NKR1/substance P (cyan), (E) isorhamnetin-3-O-rutinoside complex (red) within 0, 50, and 100 ns and experimental structure of NKR1/aprepitant (black), (F) isorhamnetin-3-O-rutinoside complex and the experimental structure of NKR1/substance P (Cyan), (G) neoxanthin complex (red) within 0, 50, and 100 ns and experimental structure of NKR1/aprepitant (black), H) neoxanthin complex and the experimental structure of NKR1/substance P (Cyan); Figure S7: Structural comparison between structures; intracellular view of experimental CLR1–zafirlukast and (A) all structures, (B) MD CLR1–zafirlukast, (C) CLR1–alpha-tocotrienol (red), (D) CLR1–neoxanthin (blue); extracellular view of CLR1–zafirlukast and (E) all structures, (F) MD CLR1–zafirlukast, (G) CLR1–alpha-tocotrienol (red), (H) CLR1–neoxanthin (blue); Figure S8: CLR1 structural comparison (extracellular view) of (A) alpha-tocotrienol complex (red) within 0, 50, and 100 ns and experimental structure of CLR1/zafirlukast (black), (B) alpha-tocotrienol complex at 0 ns (red) and 100 ns (orange), (C) MD zafirlukast complex (silver) within 0, 50, and 100 ns and experimental structure of CLR1/zafirlukast (black), (D) zafirlukast complex at 0 ns (silver) and 100 ns (grey), (E) alpha-tocotrienol complex (red) and MD zafirlukast at 100 ns (silver); Figure S9: Structural comparison between structures; lateral view of experimental CRTH2–15R-methyl-PGD2 and (A) all structures, (B) experimental structure of CRTH2–fevipiprant (silver), (C) MD CRTH2–fevipiprant (black), (D) CRTH2–alpha-tocotrienol (red), E) CRTH2–amentoflavone (green), and (F) CRTH2–neoxanthin (blue); Figure S10: Intracellular view of experimental BK2R–Gq–bradykinin and (A) all structures, (B) BK2R–JSM-10292 (black), (C) BK2R–alpha-tocotrienol (red), (D) BK2R–amentoflavone (green), (E) BK2R–isorhamnetin-3-O-rutinoside (purple), and (F) BK2R–neoxanthin (blue); extracellular view of BK2R–Gq–bradykinin and H) BK2R–JSM-10292, (I) BK2R–alpha-tocotrienol, (J) BK2R–amentoflavone, (K) BK2R–isorhamnetin-3-O-rutinoside, and (M) BK2R–neoxanthin; Figure S11: BK2R structural comparison (extracellular view) of (A) alpha-tocotrienol complex (red) within 0, 50, and 100 ns and experimental structure of BK2R/bradykinin (cyan), (B) alpha-tocotrienol complex at 0 ns (red) and 100 ns (orange), (C) JSM-10292 complex (silver) within 0, 50, and 100 ns and experimental structure of BK2R/bradykinin (cyan), (D) JSM-10292 complex at 0 ns (silver) and 100 ns (grey), (E) alpha-tocotrienol complex (red) and JSM-10292 at 100 ns (silver); Table S17: UD’s phytochemical database; Table S18: Structure used and detailed information about the system characteristics; Figure S12: Illustration of the 3D re-docking process with the co-crystallized ligands for each receptor, utilizing both software platforms; (A) HR1, (B) NK1R, (C) CLR1, (D) CRTH2, and (E) BK2R; Figure S13: Illustration of the 2D re-docking process with the co-crystallized ligands (first image) utilizing Vina (second image, and MOE (last image). (A) HR1, (B) NK1R, (C) CLR1, (D) CRTH2, and (E) BK2R. Figure S14: RMSD values for the redock. Movie S1: Dynamic structural changes in the alpha-tocotrienol/HR1 complex during the 100 ns of MD simulation; Movie S2: Dynamic structural changes in the (A) alpha-tocotrienol/NKR1, (B) aprepitant/NKR1, (C) isorhamnetin-3-O-rutinoside/NKR1, (D) neoxanthin/NKR1 complexes during the 100 ns of MD simulation; Movie S3: Dynamic structural changes in the (A) alpha-tocotrienol/CLR1, and (B) zafirlukast/CLR1 complexes during the 100 ns of MD simulation; Movie S4: Dynamic structural changes in the (A) alpha-tocotrienol/CRTH2, (B) fevipiprant/CRTH2, (C) amentoflavone/CRTH2, D) neoxanthin/CRTH2 complexes during the 100 ns of MD simulation; Movie S5: Dynamic structural changes in the (A) alpha-tocotrienol/BK2R, and (B) JSM-10292/BK2R complexes during the 100 ns of MD simulation.
