# Supplementary material for: Evaluation of Urtica dioica Phytochemicals against Therapeutic Targets of Allergic Rhinitis Using Computational Studies
Source: Molecules. 2024 Apr 12;29(8):1765. doi: 10.3390/molecules29081765 (PMC11052477; doi:10.3390/molecules29081765)
Supplement: Supplementary file 1 [file molecules-29-01765-s001.zip › supplementary material/Urtica Supporting Information.pdf]

Table S1. UD Phytochemicals docking values against HR1. Values in kcal/mol.

Phytochemical	PubChem CID	Moe Result	AutoVina Result	Mean value	Deviation
7α-Hydroxy sitosterol	161816	-6.2302	-8.7	-7.4651	1.74641233
Beta-Sitosterol	222284	-6.0757	-8.7	-7.38785	1.85566033
γ-sitosterol	457801	-6.0751	-8.6	-7.33755	1.78537391
Phytosterols	12303662	-5.9621	-8.7	-7.33105	1.93598766
Fexofenadine	3348	-6.5221	-8.1	-7.31105	1.11574379
Alpha-tocotrienol	5282347	-6.8213	-7.8	-7.31065	0.69204541
Cholecalciferol	5280795	-6.0886	-8.5	-7.2943	1.70511729
Solanidine	65727	-5.3695	-9.1	-7.23475	2.63786185
Hecogenin	91453	-5.1831	-9	-7.09155	2.69895587
Apigenin-7-O-glucoside	44257792	-5.8843	-7.9	-6.89215	1.42531514
Neoxanthin	5282217	-7.1123	-6.6	-6.85615	0.3622508
Piperine	638024	-5.409	-8	-6.7045	1.83211367
8-dehydrogingerdione	131752598	-6.2961	-7.1	-6.69805	0.56844314
6-aminochrysene	17534	-4.4932	-8.8	-6.6466	3.04536749
Chlorogenic acid	1794427	-5.9669	-7.3	-6.63345	0.94264405
3,4-dimethoxychalcone	5354494	-5.3228	-7.9	-6.6114	1.8223556
10-gingerdione	5317591	-6.255	-6.9	-6.5775	0.45608387
Estra-1,3,5(10)-trien-17B-ol	9811784	-4.7996	-8.3	-6.5498	2.47515658
Carnosol	442009	-5.2475	-7.8	-6.52375	1.80489006
Dicaffeoylquinic acid	12358846	-6.838	-6.2	-6.519	0.45113413
4-O-Caffeoylquinic acid	58427569	-5.8592	-7.1	-6.4796	0.87737809
Epicatechin gallate	107905	-5.6161	-7.3	-6.45805	1.19069711
Caffeoylquinic acid	10155076	-5.6958	-7.2	-6.4479	1.06363002
Epigallocatechin gallate	65064	-5.6623	-7.2	-6.43115	1.0873181
Sitosterol-β-D-glucoside	91884650	-6.5006	-6.3	-6.4003	0.14184562
Farnesylacetone	1711945	-5.8731	-6.9	-6.38655	0.72612795
Amentoflavone	5281600	-5.9243	-6.8	-6.36215	0.61921341
Loratadine	3957	-5.2159	-7.5	-6.35795	1.6151026
Apoatropine	64695	-5.4616	-7.2	-6.3308	1.22923443
Arachidonic acid	444899	-6.3373	-6.3	-6.31865	0.02637508
Incensole oxide	90470329	-5.12	-7.5	-6.31	1.68291414
Caffeoyl feruloyl tartaric acid	129724266	-5.9103	-6.7	-6.30515	0.55840223
Flavone	10680	-4.8753	-7.7	-6.28765	1.99736452
Quercetin dihexoside	5320835	-6.3459	-6.2	-6.27295	0.10316688
Linolenic acid	5280934	-5.8042	-6.7	-6.2521	0.63342625
[1,1-Bicyclopropyl-2-octanoic acid 2hexyl-methyl ester	50930793	-6.1551	-6.3	-6.22755	0.10245977

Anthocyanins	145858	-4.4443	-8	-6.22215	2.51425958
Convolvamine	420422	-5.5266	-6.9	-6.2133	0.97114045
Integerrimine	5281733	-4.9231	-7.5	-6.21155	1.82214346
Isopilosine	72312	-5.3142	-7.1	-6.2071	1.26275129
Phytol	5280435	-6.1094	-6.3	-6.2047	0.13477455
Pukateine	442340	-4.5964	-7.8	-6.1982	2.26528728
Luteolin	5280445	-5.1558	-7.2	-6.1779	1.44546768
Neochlorogenic acid	5280633	-5.8372	-6.5	-6.1686	0.46867037
Flavan	94156	-4.6058	-7.7	-6.1529	2.1879298
β - Sesquiphellandrene	519764	-5.3973	-6.9	-6.14865	1.06256936
Neophytadiene	10446	-5.8931	-6.4	-6.14655	0.35843243
Homatropine	5282593	-5.391	-6.9	-6.1455	1.06702413
Isorhamnetin 3-O-rutinoside	5481663	-6.2764	-6	-6.1382	0.19544431
Dihydrokavain	10220256	-5.1578	-7.1	-6.1289	1.37334279
Pterostilbene	5281727	-5.4191	-6.8	-6.10955	0.97644375
Crysoeriol	5280666	-5.0917	-7.1	-6.09585	1.42008255
4-shogaol	9794897	-5.6454	-6.5	-6.0727	0.60429346
Isorhamnetin rutinoside	133562525	-6.4382	-5.7	-6.0691	0.52198623
Apigenin	5280443	-4.9978	-7.1	-6.0489	1.48647988
Isorhamnetin	5281654	-5.3939	-6.7	-6.04695	0.92355217
Podocarpic acid	93017	-4.5656	-7.5	-6.0328	2.07493414
Kaempferol-3-rutinoside	5318767	-6.0868	-5.9	-5.9934	0.13208755
Isolariciresinol	160521	-5.2855	-6.7	-5.99275	1.00020254
ar-Curcumene	92139	-5.0782	-6.9	-5.9891	1.28820713
7β-Hydroxy-sitosterol	12309569	-6.0482	-5.9	-5.9741	0.10479322
Incensole oxide acetate	73755086	-5.3141	-6.6	-5.95705	0.90926861
β-Bisabolene	10104370	-4.9702	-6.9	-5.9351	1.36457467
9-Hydroxy-10,12-octadecadienoic acid	1927	-5.6493	-6.2	-5.92465	0.3894037
Benzyl salicilate	8363	-4.9442	-6.9	-5.9221	1.38295944
Palmitic acid	985	-5.8237	-6	-5.91185	0.12466293
Naringenin	932	-4.9156	-6.9	-5.9078	1.4031827
Ursolic acid	64945	-5.6005	-6.2	-5.90025	0.42391052
Kaempferol	5280863	-4.9907	-6.8	-5.89535	1.2793683
Palmitoleic acid	445638	-5.6874	-6.1	-5.8937	0.29175226
α- Copaene-8-ol	25086830	-4.9863	-6.8	-5.89315	1.28247957
α-Humulene	5281520	-4.7726	-7	-5.8863	1.57500964
β - Vetivenene	14475467	-4.6601	-7.1	-5.88005	1.72526984
α-Copaene	442355	-4.7449	-7	-5.87245	1.5945965
cis-10-Heptadecenoic acid	5312435	-5.9435	-5.8	-5.87175	0.10146982
Farnesol	445070	-5.3238	-6.4	-5.8619	0.76098832
Quercetin rhamnoside	15939939	-5.5134	-6.2	-5.8567	0.48549952

(+)-Neo-olivil	9976812	-5.3091	-6.4	-5.85455	0.77138279
Caffeoylmalic acid	6124299	-5.3076	-6.4	-5.8538	0.77244345
Eleutheroside B	5316860	-5.9924	-5.7	-5.8462	0.20675802
Hexahydrofarnesylacetone	10408	-5.8855	-5.8	-5.84275	0.06045763
Doxepin	3158	-4.9623	-6.7	-5.83115	1.22873945
Kavain	5281565	-4.9584	-6.7	-5.8292	1.23149717
Deoxyharringtonine	285342	-5.5562	-6.1	-5.8281	0.38452467
δ-Cadinene	441005	-4.7456	-6.9	-5.8228	1.52339085
Feruloyl malate	71694479	-5.3202	-6.3	-5.8101	0.69282322
β-Selinene	442393	-4.6981	-6.9	-5.79905	1.55697842
Isorhamnetin-3-O-glucoside	5318645	-5.5906	-6	-5.7953	0.28948952
6-benzylaminopurine	62389	-4.3875	-7.2	-5.79375	1.98873782
Myristoleic acid	5281119	-5.5859	-6	-5.79295	0.29281292
Heptadecanoic acid	10465	-5.9788	-5.6	-5.7894	0.26785205
Secoisolariciresinol	65373	-5.7776	-5.8	-5.7888	0.01583919
β-Caryophyllene	5281515	-4.8524	-6.7	-5.7762	1.30645049
Genistein	5280961	-4.8508	-6.7	-5.7754	1.30758186
1,2-Diguaiacyl-1,3-propanediol	6426042	-5.1505	-6.4	-5.77525	0.88352992
Kaempferol rhamnoside	5835713	-5.436	-6.1	-5.768	0.4695189
α-Selinene	10856614	-4.7329	-6.8	-5.76645	1.46166043
Quercetin	5280343	-5.1021	-6.4	-5.75105	0.91775389
Catechin hydrate	107957	-5.1846	-6.3	-5.7423	0.7887069
Caffeoyl tartaric acid	9857913	-5.3824	-6.1	-5.7412	0.50741983
Myricetin	5281672	-5.1685	-6.3	-5.73425	0.80009132
Isopropyl dodecanoate	25068	-5.5619	-5.9	-5.73095	0.2390728
2H-Indeno[1,2-b]furan-2-one, 3,3a,4,5,6,7,8,8b-octahydro-8,8-dimethyl	605626	-4.4332	-7	-5.7166	1.81500169
α-Longipinene	520957	-4.8302	-6.6	-5.7151	1.25143758
γ-Cadinene	92313	-4.706	-6.7	-5.703	1.40997092
Methyl palmitate	8181	-5.9944	-5.4	-5.6972	0.42030427
Geranyl acetone	1549778	-5.0838	-6.3	-5.6919	0.85998327
Abscisic acid	5375199	-4.7747	-6.6	-5.68735	1.29068201
Epicatechin	72276	-4.814	-6.5	-5.657	1.19218203
Quercetin-3-O-glucoside	25203368	-5.4126	-5.9	-5.6563	0.34464385
Osthole	10228	-4.6725	-6.6	-5.63625	1.36294832
Caryophyllene oxide	1742210	-4.5483	-6.7	-5.62415	1.52148166
Kaempferol-3-O-glucoside	5282102	-5.4427	-5.8	-5.62135	0.25264925
(E)-Geranyl acetone	1713001	-5.1202	-6.1	-5.6101	0.69282322
Bornyl acetate	6448	-4.6168	-6.6	-5.6084	1.40233417

1-Hydroxy-1-(4-hydroxyphenyl)-2-propanone	10261435	-5.5086	-5.7	-5.6043	0.13534024
Catechin	9064	-4.9065	-6.3	-5.60325	0.9853533
Kaempferol pentoside	14749097	-5.1918	-6	-5.5959	0.5714837
Quercitrin	5280459	-5.2485	-5.9	-5.57425	0.46068007
β-Bourbonene	62566	-4.4423	-6.7	-5.57115	1.59643498
Quercetin-3-glucoside	5280804	-5.2398	-5.9	-5.5699	0.4668319
Myristic acid	11005	-5.50003	-5.6	-5.550015	0.07068946
Diphenyl	7095	-4.2435	-6.8	-5.52175	1.80771849
Peonidin 3-O-rutinoside	44256842	-5.932	-5.1	-5.516	0.58831284
Arbutine	440936	-5.0019	-6	-5.50095	0.70576328
Bioallethrin	15558638	-5.413	-5.5	-5.4565	0.06151829
Harmine	5280953	-4.5792	-6.3	-5.4396	1.21678935
4-methyl-7-ethoxycoumarin	66595	-4.5872	-6.2	-5.3936	1.14042182
Rutin	5280805	-5.976	-4.8	-5.388	0.83155757
1-Methyl naphthalene	7002	-4.0664	-6.7	-5.3832	1.86223642
Calamenene	6429077	-4.5427	-6.2	-5.37135	1.17188807
Carvacryl acetate	80792	-4.6375	-6.1	-5.36875	1.03414367
Isotachioside	15098566	-5.0276	-5.7	-5.3638	0.4754586
Harmol	68094	-4.3052	-6.4	-5.3526	1.48124729
4-(3-Hydroxy-1-butyn-1-yl)- 3,5,5-trimethyl-2-cyclohexen-1-ol	5280654	-4.6033	-6.1	-5.35165	1.05832672
Dibutyl phthalate	3026	-5.2871	-5.4	-5.34355	0.07983236
α-Ionone	5282108	-4.6817	-6	-5.34085	0.93217887
Shikimic acid	8742	-4.3748	-6.3	-5.3374	1.36132198
4-methylmethoxycoumarin	223821	-4.4742	-6.2	-5.3371	1.22032488
p-Coumaroylmalic acid	129720114	-4.97	-5.7	-5.335	0.51618795
1-Dodecanamine, N, N-dimethyl	8168	-5.3427	-5.3	-5.32135	0.03019346
Carvylacetate	7335	-4.9298	-5.7	-5.3149	0.54461364
α- Terpinyl acetate	111037	-4.6231	-6	-5.31155	0.97361533
3-Hydroxy-damascone	5366075	-4.8226	-5.8	-5.3113	0.69112617
Adenosine	60961	-4.9112	-5.7	-5.3056	0.55776583
Tachioside	11962143	-4.7943	-5.8	-5.29715	0.71113729
Dodecendioic acid	5283028	-5.0907	-5.5	-5.29535	0.28941881
9-amino-camptothecin	72402	-5.0602	-5.5	-5.2801	0.31098556
Silane, triethyl(2-phenylethoxy)	610043	-5.5421	-5	-5.27105	0.38332259
O-Feruloyl quinic acid	10177048	-5.8282	-4.7	-5.2641	0.79775787
Hydrocotarnine	3646	-4.3891	-6.1	-5.24455	1.20978899

DL-methyl-m-tyrosine	2110	-4.6843	-5.8	-5.24215	0.78891904
Cinnamyl acetate	5282110	-4.6839	-5.8	-5.24195	0.78920188
β-Ionone	638014	-4.3642	-6.1	-5.2321	1.22739595
Lauric acid	3893	-5.0462	-5.4	-5.2231	0.25017438
4-(4-Hydroxy-2,6,6-trimethyl1-cyclohexen-1-yl)-3-buten2-one	538953	-4.5395	-5.9	-5.21975	0.96201878
1,2-Benzenedicarboxylic acid	90531	-5.1265	-5.3	-5.21325	0.12268303
Safranal	61041	-4.397	-6	-5.1985	1.13349217
4-aminoantipyrine	2151	-4.4812	-5.9	-5.1906	1.0032431
Carvone	7439	-4.4656	-5.9	-5.1828	1.01427397
Cinnamide	5273472	-4.4598	-5.9	-5.1799	1.01837519
Linalool	6549	-4.4213	-5.9	-5.16065	1.0455988
4-Acetyl-2-methylphenol	70135	-4.4034	-5.9	-5.1517	1.05825601
3,4-Dimethyl-5-pentylidene-2(5H)-furanone	6433214	-4.5843	-5.7	-5.14215	0.78891904
Quinic acid	6508	-4.0803	-6.2	-5.14015	1.49885424
Phenylalanine	6140	-4.458	-5.8	-5.129	0.9489373
Carvacrol	10364	-4.3578	-5.9	-5.1289	1.09050008
3,4-Dimethyl-5-pentylfuran-2(5H)-one	13192443	-4.6535	-5.6	-5.12675	0.66927657
2(3H)-Naphthalenone, 4, 4a,5,6,7,8- hexahydro-1-methoxy	534313	-4.4365	-5.8	-5.11825	0.9641401
Methylcoumarin	17130	-4.0304	-6.2	-5.1152	1.53413887
3-Oxo-a-ionol	5370052	-4.49444	-5.7	-5.09722	0.85245965
Camphor	2537	-4.2732	-5.9	-5.0866	1.15032131
Carnosine	439224	-4.8235	-5.3	-5.06175	0.33693638
n-acetyl-L-glutamine	182230	-4.4877	-5.6	-5.04385	0.78651487
Umbelliferone	5281426	-4.2833	-5.8	-5.04165	1.07246886
2-Pentylfuran	19602	-4.3619	-5.7	-5.03095	0.94617958
Thymol	6989	-4.3415	-5.7	-5.02075	0.96060456
Cinnamic acid	444539	-4.2413	-5.8	-5.02065	1.10216734
1,2-O-isopropylidene-D-glucoside	87704	-4.729	-5.3	-5.0145	0.40375797
Levulinic acid	11579	-4.6258	-5.4	-5.0129	0.54744207
Naphthalene	931	-3.9013	-6.1	-5.00065	1.55471568
p-Cymene	7463	-4.1664	-5.8	-4.9832	1.15512964
Borneol	64685	-4.4565	-5.5	-4.97825	0.73786593
Caffeic acid	689043	-4.4034	-5.5	-4.9517	0.7754133
Esculetin	5281416	-4.2483	-5.6	-4.92415	0.95579624
Hydroxycinnamaldehyde	71407359	-4.2442	-5.6	-4.9221	0.95869537

Anthranilic acid methyl ester	8635	-4.2379	-5.6	-4.91895	0.96315015
α- Terpineol	17100	-4.3346	-5.5	-4.9173	0.82406224
2-Heptanone	8051	-4.3068	-5.5	-4.9034	0.84371981
3-Octanone	246728	-4.1028	-5.7	-4.9014	1.12939095
Citric acid	311	-4.098	-5.7	-4.899	1.13278506
Methyl eugenol	7127	-4.5654	-5.2	-4.8827	0.44872996
Heptanal	8130	-4.4647	-5.3	-4.88235	0.59064629
β-Homocyclocitral	61124	-4.4497	-5.3	-4.87485	0.6012529
Acetophenone	7410	-4.2438	-5.5	-4.8719	0.88826754
Methyl chavicol	8815	-4.4351	-5.3	-4.86755	0.61157666
Lotaustralin	441467	-4.6284	-5.1	-4.8642	0.33347156
2,4,6-Trimethyl-5H-1,3,5-dithiazine	12518	-4.0211	-5.7	-4.86055	1.18716157
Nonanal	31289	-4.4989	-5.2	-4.84945	0.49575256
Phosphatidylcholine	10425706	-5.2752	-4.4	-4.8376	0.61885985
Succinic acid	1110	-4.3727	-5.3	-4.83635	0.65570012
3,4-Dihydroxybenzoic acid	72	-4.1666	-5.5	-4.8333	0.94285618
Decanal	8175	-4.7648	-4.9	-4.8324	0.09560084
L-2-aminoadipic acid	92136	-4.1413	-5.5	-4.82065	0.96074598
Indole	798	-4.0359	-5.6	-4.81795	1.10598572
Ferulic acid	445858	-4.3353	-5.3	-4.81765	0.68214591
p-hydroxybenzoic acid	135	-3.9341	-5.7	-4.81705	1.24867986
Salicylic alcohol	5146	-4.4245	-5.2	-4.81225	0.54836131
Benzofuran, 2,3,-dihydro	10329	-4.1214	-5.5	-4.8107	0.97481741
Cumin aldehyde	326	-4.2134	-5.4	-4.8067	0.83905291
Gentisic acid	3469	-4.3071	-5.3	-4.80355	0.70208632
Indole-3-carboxaldehyde	10256	-3.8933	-5.7	-4.79665	1.27752982
2-(1-Pentenyl)furan	5369956	-4.5891	-5	-4.79455	0.29055018
β-Cyclocitral	9895	-4.2793	-5.3	-4.78965	0.72174389
n-Octanal	454	-4.1711	-5.4	-4.78555	0.86896352
(E)-Anethole	637563	-4.2669	-5.3	-4.78345	0.73051202
Coniferol	1549095	-4.4331	-5.1	-4.76655	0.47156951
Decan-2-one	12741	-4.5272	-5	-4.7636	0.33432009
p-coumaric acid	637542	-4.3004	-5.2	-4.7502	0.63611326
p-Hydroxy-acetophenone	7469	-3.9972	-5.5	-4.7486	1.06264007
Nonanol	8914	-4.3941	-5.1	-4.74705	0.49914668
Vitamin B5	6613	-4.5861	-4.9	-4.74305	0.22196082
Oxime- methoxy-phenyl	9602988	-3.9799	-5.5	-4.73995	1.07487302
m-Hydroxy-acetophenone	8487	-4.079	-5.4	-4.7395	0.93408806
Isopropyl-β-D-thio-galacto-pyranoside	656894	-4.5687	-4.9	-4.73435	0.23426448
Aminobutyric acid	119	-4.6407	-4.8	-4.72035	0.11264211
2-acetylpyrrole	14079	-4.4069	-5	-4.70345	0.41938503

Homovanillyl alcohol	16928	-4.4777	-4.9	-4.68885	0.29861119
Scopoletin	5280460	-4.1664	-5.2	-4.6832	0.73086557
Histidinol	776	-3.9471	-5.4	-4.67355	1.02735544
Glutaric acid	743	-3.9466	-5.4	-4.6733	1.027709
Synephrine	7172	-4.3459	-5	-4.67295	0.46251855
2-deoxy-D-ribose	5460005	-4.3193	-5	-4.65965	0.48132759
Phthalic acid	1017	-3.807	-5.5	-4.6535	1.19713178
Eugenol	3314	-4.1972	-5.1	-4.6486	0.638376
4-Vinyl guaiacol	332	-4.2574	-5	-4.6287	0.5250975
Sinapic acid	637775	-4.2393	-5	-4.61965	0.53789613
Vanillic acid	8468	-4.0338	-5.1	-4.5669	0.75391725
DL-a aminopimelic acid	101122	-4.519	-4.6	-4.5595	0.05727565
Glucosamine	439213	-4.4125	-4.7	-4.55625	0.2032932
Resorcinol	5054	-4.0108	-5.1	-4.5554	0.77018071
Gallic acid	370	-4.0073	-5.1	-4.55365	0.77265558
5,6-Dihydro-4-pentyl- 2,6-dimethyl-4H-1,3,5-dithiazine	528360	-4.3059	-4.8	-4.55295	0.34938146
Tyrosol	10393	-4.1039	-5	-4.55195	0.63363839
4-deoxypyridoxine	6094	-4.1868	-4.9	-4.5434	0.50430856
Sinapyl alcohol	5280507	-4.3737	-4.7	-4.53685	0.23072894
p-Hydroxy-benzaldehyde	126	-3.941	-5.1	-4.5205	0.81953676
p-Hydroxybenzyl alcohol	125	-4.1211	-4.9	-4.51055	0.55076547
Rhamnose	25310	-4.2847	-4.7	-4.49235	0.29366145
Leucine	6106	-4.0817	-4.9	-4.49085	0.57862548
Benzaldehyde	240	-3.9238	-5	-4.4619	0.76098832
Anisaldehyde	31244	-4.2212	-4.7	-4.4606	0.33856273
2,6-Dimethoxy-hydroquinone	96038	-4.3788	-4.5	-4.4394	0.08570134
Niacinamide	936	-3.971	-4.9	-4.4355	0.6569022
2, 6,-Nonadienal, 3, 7-dimethyl	5364526	-4.627	-4.2	-4.4135	0.3019346
Syringic acid	10742	-4.126	-4.7	-4.413	0.40587929
3-hydroxy-2,3 dihydromaltol	119838	-4.1258	-4.7	-4.4129	0.40602071
Hexanal	6184	-3.7988	-4.9	-4.3494	0.77866599
6-azacytosine	70265	-4.0896	-4.6	-4.3448	0.3609073
adenine	190	-3.8392	-4.8	-4.3196	0.6793882
Pyrogallol	1057	-3.8293	-4.8	-4.31465	0.68638855
1,6-dioxaspiro[4.4]non-3-ene	10374471	-3.8591	-4.7	-4.27955	0.59460609
Galactal	2734735	-4.2054	-4.3	-4.2527	0.0668923
Arabitol	94154	-3.9831	-4.5	-4.24155	0.3655035
Levoglucosan	2724705	-3.9712	-4.5	-4.2356	0.37391807

Dimethyl-L-tartrate	11851	-4.3304	-4	-4.1652	0.23362808
1,4-benzoquinone	4650	-3.7921	-4.5	-4.14605	0.50056089
Mannitol	6251	-4.3322	-3.9	-4.1161	0.30561155
Creatinine	588	-3.7676	-4.3	-4.0338	0.37646365
Proline	145742	-3.8125	-4.2	-4.00625	0.27400388
1, 2, 3-Butanetriol	20497	-3.6646	-4.3	-3.9823	0.44929565
L-threonine	6288	-3.7288	-4.2	-3.9644	0.33318872
3,5-Dimethyl-1,2,4-trithiolane	32033	-4.1077	-3.5	-3.80385	0.42970879
Choline	305	-3.8757	-3.2	-3.53785	0.47779205
Formic acid	284	-2.905	-2.7	-2.8025	0.14495689
Lutein	5281243	5.8577	-6.4	-0.27115	8.66750279
Violaxanthin	448438	14.2916	-6.4	3.9458	14.6311707
β-carotene	5280489	14.7245	-6.3	4.21225	14.8665665

Table S2. UD Phytochemicals docking values against NKR1. Values in kcal/mol.

Phytochemical	PubChem CID	Moe Result	AutoVina Result	Mean value	Deviation
Amentoflavone	5281600	-8.2097	-11	-9.60485	1.97304005
aprepitant	135413536	-7.6781	-10.4	-9.03905	1.92467395
Peonidin 3-O-rutinoside	44256842	-7.8534	-9.3	-8.5767	1.02290067
Isorhamnetin 3-O-rutinoside	5481663	-8.2394	-8.8	-8.5197	0.39640406
Quercitrin	5280459	-7.5104	-9.4	-8.4552	1.33614897
Quercetin dihexoside	5320835	-7.841	-9	-8.4205	0.81953676
Kaempferol-3-rutinoside	5318767	-7.7085	-9.1	-8.40425	0.98393909
Alpha-tocotrienol	5282347	-7.8049	-9	-8.40245	0.84506331
Isorhamnetin-3-O-glucoside	5318645	-7.3334	-9.4	-8.3667	1.46130687
Quercetin-3-O-glucoside	25203368	-7.3537	-9	-8.17685	1.16410989
Quercetin-3-glucoside	5280804	-7.5809	-8.7	-8.14045	0.7913232
Dicaffeoylquinic acid	12358846	-7.5571	-8.7	-8.12855	0.80815234
Neoxanthin	5282217	-7.129	-8.8	-7.9645	1.18157543
7α-Hydroxy sitosterol	161816	-6.6276	-9.3	-7.9638	1.88967216
Hecogenin	91453	-6.3077	-9.6	-7.95385	2.32800766
Sitosterol-β-D-glucoside	91884650	-7.1062	-8.8	-7.9531	1.19769747
Quercetin rhamnoside	15939939	-7.2971	-8.6	-7.94855	0.92128943
Kaempferol-3-O-glucoside	5282102	-7.3247	-8.5	-7.91235	0.8310626
Lutein	5281243	-7.9512	-7.8	-7.8756	0.10691455
Caffeoyl feruloyl tartaric acid	129724266	-7.8099	-7.9	-7.85495	0.06371032

Epigallocatechin gallate	65064	-6.7522	-8.9	-7.8261	1.51872394
Isorhamnetin rutinoside	133562525	-7.3297	-8.3	-7.81485	0.68610571
Apigenin-7-O-glucoside	44257792	-6.7707	-8.8	-7.78535	1.43493179
γ-sitosterol	457801	-6.5771	-8.9	-7.73855	1.64253834
Kaempferol rhamnoside	5835713	-7.0723	-8.4	-7.73615	0.93882567
7β-Hydroxy-sitosterol	12309569	-6.6034	-8.8	-7.7017	1.55323076
β-carotene	5280489	-7.6738	-7.7	-7.6869	0.0185262
Cholecalciferol	5280795	-6.8957	-8.4	-7.64785	1.06370073
Violaxanthin	448438	-8.1867	-7	-7.59335	0.83912362
Beta-Sitosterol	222284	-6.3332	-8.8	-7.5666	1.74429101
Phytosterols	12303662	-6.2539	-8.8	-7.52695	1.80036458
Kaempferol pentoside	14749097	-6.5829	-8.4	-7.49145	1.28488373
9-amino-camptothecin	72402	-5.8327	-9	-7.41635	2.23961931
Solanidine	65727	-5.4999	-9.2	-7.34995	2.6163658
Pukateine	442340	-5.7947	-8.9	-7.34735	2.19577869
Epicatechin gallate	107905	-6.4591	-8.2	-7.32955	1.2310022
6-aminochrysene	17534	-5.2395	-9.2	-7.21975	2.80049641
Deoxyharringtonine	285342	-6.6155	-7.8	-7.20775	0.83756798
10-gingerdione	5317591	-7.5926	-6.8	-7.1963	0.56045283
Estra-1,3,5(10)-trien-17B-ol	9811784	-5.3792	-9	-7.1896	2.56029223
Bioallethrin	15558638	-6.7447	-7.6	-7.17235	0.60478843
Isolariciresinol	160521	-6.5878	-7.7	-7.1439	0.78644416
Ursolic acid	64945	-5.5652	-8.7	-7.1326	2.21663834
Luteolin	5280445	-6.027	-8.1	-7.0635	1.46583236
Chlorogenic acid	1794427	-6.7235	-7.4	-7.06175	0.47835774
(+)-Neo-olivil	9976812	-6.7121	-7.4	-7.05605	0.48641875
4-O-Caffeoylquinic acid	58427569	-6.2251	-7.8	-7.01255	1.11362247
O-Feruloyl quinic acid	10177048	-6.4243	-7.6	-7.01215	0.83134544
Piperine	638024	-5.9949	-8	-6.99745	1.41781981
Safranal	61041	-4.5541	-9.4	-6.97705	3.42656875
Crysoeriol	5280666	-5.9006	-8	-6.9503	1.48449998
[1,1-Bicyclopropyl-2-octanoic acid 2hexyl-methyl ester	50930793	-7.2295	-6.6	-6.91475	0.44512372
Neochlorogenic acid	5280633	-6.0177	-7.8	-6.90885	1.26027642
Secoisolariciresinol	65373	-7.1729	-6.6	-6.88645	0.40510147
Isopilosine	72312	-6.2692	-7.5	-6.8846	0.87030703
1,2-Diguaiacyl-1,3-propanediol	6426042	-6.2579	-7.5	-6.87895	0.87829733
Eleutheroside B	5316860	-7.0342	-6.7	-6.8671	0.23631509
Incensole oxide acetate	73755086	-6.8185	-6.9	-6.85925	0.0576292
Caffeoylquinic acid	10155076	-6.0562	-7.6	-6.8281	1.09163145
Convolvamine	420422	-6.509	-7.1	-6.8045	0.41790011

Epicatechin	72276	-5.8703	-7.7	-6.78515	1.29379328
Farnesylacetone	1711945	-6.7688	-6.8	-6.7844	0.02206173
Catechin	9064	-5.8533	-7.7	-6.77665	1.30581409
Apigenin	5280443	-5.6352	-7.9	-6.7676	1.60145544
Catechin hydrate	107957	-5.806	-7.7	-6.753	1.33926024
2H-Indeno[1,2-b]furan-2-one, 3,3a,4,5,6,7,8,8b-octahydro-8,8-dimethyl	605626	-5.7631	-7.7	-6.73155	1.36959512
3,4-dimethoxychalcone	5354494	-6.0573	-7.4	-6.72865	0.94943228
Incensole oxide	90470329	-5.7429	-7.7	-6.72145	1.38387868
Isorhamnetin	5281654	-5.9095	-7.5	-6.70475	1.12465334
Apoatropine	64695	-5.6661	-7.7	-6.68305	1.43818448
α-Humulene	5281520	-5.6232	-7.7	-6.6616	1.46851936
Genistein	5280961	-5.52	-7.8	-6.66	1.61220346
Myricetin	5281672	-6.0142	-7.3	-6.6571	0.9091979
Naringenin	932	-5.5023	-7.8	-6.65115	1.62471925
Quinic acid	6508	-4.2983	-9	-6.64915	3.32460395
γ-Cadinene	92313	-5.5976	-7.7	-6.6488	1.4866213
Tachioside	11962143	-6.1929	-7.1	-6.64645	0.64141656
Homatropine	5282593	-6.071	-7.2	-6.6355	0.79832356
Caffeoyl tartaric acid	9857913	-6.3708	-6.9	-6.6354	0.37420091
Arachidonic acid	444899	-7.0676	-6.2	-6.6338	0.61348584
δ-Cadinene	441005	-5.4153	-7.8	-6.60765	1.68623754
Osthole	10228	-5.6051	-7.6	-6.60255	1.41060732
Podocarpic acid	93017	-5.1965	-7.9	-6.54825	1.91166318
Feruloyl malate	71694479	-6.3724	-6.7	-6.5362	0.23164818
Abscisic acid	5375199	-5.2976	-7.7	-6.4988	1.69875333
Carnosol	442009	-5.3967	-7.6	-6.49835	1.55796837
β-Bisabolene	10104370	-6.2887	-6.7	-6.49435	0.29083302
Hexahydrofarnesylacetone	10408	-6.5786	-6.4	-6.4893	0.12628927
Isotachioside	15098566	-6.2779	-6.7	-6.48895	0.29846977
Linolenic acid	5280934	-6.7772	-6.2	-6.4886	0.40814203
Quercetin	5280343	-5.4736	-7.5	-6.4868	1.43288118
Kaempferol	5280863	-5.5556	-7.4	-6.4778	1.30418775
Flavone	10680	-5.3199	-7.6	-6.45995	1.61227417
8-dehydrogingerdione	131752598	-6.5904	-6.3	-6.4452	0.20534381
β-Bourbonene	62566	-5.0839	-7.8	-6.44195	1.92057273
4-(4-Hydroxy-2,6,6-trimethyl1-cyclohexen-1-yl)-3-buten2-one	538953	-5.658	-7.2	-6.429	1.09035866
Integerrimine	5281733	-5.356	-7.5	-6.428	1.51603694
Caffeoylmalic acid	6124299	-5.9523	-6.9	-6.42615	0.6701251
9-Hydroxy-10,12-octadecadienoic acid	1927	-6.7009	-6.1	-6.40045	0.42490046

Pterostilbene	5281727	-5.7862	-7	-6.3931	0.85828621
Calamenene	6429077	-5.239	-7.5	-6.3695	1.59876843
Anthocyanins	145858	-5.1373	-7.6	-6.36865	1.74139187
Farnesol	445070	-6.4892	-6.2	-6.3446	0.20449528
Phytol	5280435	-6.3661	-6.3	-6.33305	0.04673976
Dihydrokavain	10220256	-5.4632	-7.2	-6.3316	1.22810306
Dibutyl phthalate	3026	-6.4139	-6.2	-6.30695	0.15125014
6-benzylaminopurine	62389	-5.3962	-7.2	-6.2981	1.27547921
α-Copaene	442355	-4.8381	-7.7	-6.26905	2.0236689
Flavan	94156	-5.0095	-7.5	-6.25475	1.76104944
Neophytadiene	10446	-6.3725	-6.1	-6.23625	0.1926866
4-shogaol	9794897	-5.8641	-6.6	-6.23205	0.52035988
p-Coumaroylmalic acid	129720114	-5.4941	-6.9	-6.19705	0.99412142
cis-10-Heptadecenoic acid	5312435	-6.6736	-5.7	-6.1868	0.68843916
Harmine	5280953	-5.2467	-7.1	-6.17335	1.310481
β-Caryophyllene	5281515	-4.8463	-7.5	-6.17315	1.87644927
α- Copaene-8-ol	25086830	-5.0054	-7.3	-6.1527	1.62252722
Benzyl salicilate	8363	-5.3017	-7	-6.15085	1.20087945
ar-Curcumene	92139	-5.6346	-6.6	-6.1173	0.68264089
Isopropyl dodecanoate	25068	-6.4074	-5.8	-6.1037	0.42949666
β - Sesquiphellandrene	519764	-5.6727	-6.5	-6.08635	0.58498944
β - Vetivenene	14475467	-4.972	-7.2	-6.086	1.57543391
Methyl palmitate	8181	-6.5632	-5.6	-6.0816	0.68108525
Hydrocotarnine	3646	-5.5587	-6.6	-6.07935	0.73631029
Palmitic acid	985	-6.5314	-5.6	-6.0657	0.65859926
β-Ionone	638014	-5.23	-6.9	-6.065	1.18086832
1,2-Benzenedicarboxylic acid	90531	-5.8178	-6.3	-6.0589	0.34096689
Rutin	5280805	-7.6162	-4.5	-6.0581	2.20348615
Adenosine	60961	-5.497	-6.6	-6.0485	0.77993878
4-methyl-7-ethoxycoumarin	66595	-5.1684	-6.9	-6.0342	1.2244261
Kavain	5281565	-5.1301	-6.9	-6.01505	1.25150829
Arbutine	440936	-5.3238	-6.7	-6.0119	0.97312035
Caryophyllene oxide	1742210	-4.8048	-7.2	-6.0024	1.69366216
3-Oxo-a-ionol	5370052	-5.2944	-6.7	-5.9972	0.99390929
β-Selinene	442393	-4.7669	-7.2	-5.98345	1.72046151
α-Ionone	5282108	-5.3431	-6.6	-5.97155	0.88876251
α-Selinene	10856614	-4.7041	-7.2	-5.95205	1.76486782
Carvacryl acetate	80792	-5.2979	-6.5	-5.89895	0.85001306
Heptadecanoic acid	10465	-6.5887	-5.2	-5.89435	0.98195919
Carvylacetate	7335	-5.2853	-6.5	-5.89265	0.85892261
Harmol	68094	-4.7853	-7	-5.89265	1.56602939

Geranyl acetone	1549778	-5.7688	-6	-5.8844	0.16348309
(E)-Geranyl acetone	1713001	-5.7645	-6	-5.88225	0.16652365
3-Hydroxy-damascone	5366075	-5.2355	-6.5	-5.86775	0.89413652
4-methylmethoxycoumarin	223821	-4.9655	-6.7	-5.83275	1.22647671
Palmitoleic acid	445638	-5.9622	-5.7	-5.8311	0.1854034
Myristoleic acid	5281119	-6.0621	-5.6	-5.83105	0.32675404
2(3H)-Naphthalenone, 4, 4a,5,6,7,8- hexahydro-1-methoxy	534313	-5.0231	-6.6	-5.81155	1.11503668
Myristic acid	11005	-6.1039	-5.5	-5.80195	0.42702179
Shikimic acid	8742	-4.297	-7.3	-5.7985	2.12344166
3,4-Dimethyl-5-pentylidene-2(5H)-furanone	6433214	-5.4309	-6.1	-5.76545	0.47312515
Phosphatidylcholine	10425706	-6.165	-5.3	-5.7325	0.61164737
α- Terpinyl acetate	111037	-5.3424	-6.1	-5.7212	0.5357041
Silane, triethyl(2-phenylethoxy)	610043	-6.1883	-5.2	-5.69415	0.69883363
Lotaustralin	441467	-5.6701	-5.7	-5.68505	0.02114249
1-Dodecanamine, N, N-dimethyl	8168	-6.2021	-5.1	-5.65105	0.77930238
α-Longipinene	520957	-4.1861	-7.1	-5.64305	2.06043845
Eugenol	3314	-5.2433	-6	-5.62165	0.5350677
DL-methyl-m-tyrosine	2110	-4.9145	-6.3	-5.60725	0.97969645
Cinnamyl acetate	5282110	-5.0907	-6.1	-5.59535	0.71368287
Methyl eugenol	7127	-5.3646	-5.8	-5.5823	0.30787429
Diphenyl	7095	-4.6319	-6.5	-5.56595	1.32094618
4-(3-Hydroxy-1-butyn-1-yl)- 3,5,5-trimethyl-2-cyclohexen-1-ol	5280654	-4.8283	-6.3	-5.56415	1.04064905
Sinapyl alcohol	5280507	-5.4115	-5.7	-5.55575	0.20400031
4-aminoantipyrine	2151	-4.7989	-6.3	-5.54945	1.06143799
β-Homocyclocitral	61124	-4.7859	-6.3	-5.54295	1.07063038
Ferulic acid	445858	-4.8534	-6.2	-5.5267	0.95218999
Methylcoumarin	17130	-4.4263	-6.6	-5.51315	1.53703801
3,4-Dimethyl-5-pentylfuran-2(5H)-one	13192443	-5.2729	-5.7	-5.48645	0.30200531
Sinapic acid	637775	-5.0139	-5.9	-5.45695	0.62656732
Coniferol	1549095	-5.0135	-5.9	-5.45675	0.62685016
Carvacrol	10364	-4.9454	-5.9	-5.4227	0.67500413
Dodecendioic acid	5283028	-5.6256	-5.2	-5.4128	0.30094465
1-Methyl naphthalene	7002	-4.244	-6.5	-5.372	1.5952329
Esculetin	5281416	-4.417	-6.3	-5.3585	1.33148207
Carnosine	439224	-5.0159	-5.7	-5.35795	0.48373175

Thymol	6989	-4.8135	-5.9	-5.35675	0.76827152
1,2-O-isopropylidene-D-glucoside	87704	-5.1923	-5.5	-5.34615	0.21757676
Phenylalanine	6140	-4.9566	-5.7	-5.3283	0.52566318
Umbelliferone	5281426	-4.3147	-6.3	-5.30735	1.40381909
4-Acetyl-2-methylphenol	70135	-4.7125	-5.9	-5.30625	0.8396893
Carvone	7439	-4.7003	-5.9	-5.30015	0.84831601
Syringic acid	10742	-4.9963	-5.6	-5.29815	0.42688036
p-Cymene	7463	-4.7899	-5.8	-5.29495	0.71424856
β-Cyclocitral	9895	-4.5274	-6	-5.2637	1.04128545
Isopropyl-β-D-thio-galacto-pyranoside	656894	-5.1108	-5.4	-5.2554	0.20449528
Cumin aldehyde	326	-4.6846	-5.8	-5.2423	0.7887069
(E)-Anethole	637563	-4.6844	-5.8	-5.2422	0.78884833
Vitamin B5	6613	-5.1835	-5.3	-5.24175	0.08237794
Lauric acid	3893	-5.3441	-5.1	-5.22205	0.17260477
Caffeic acid	689043	-4.537	-5.9	-5.2185	0.96378654
Methyl chavicol	8815	-4.8043	-5.6	-5.20215	0.56264487
Homovanillyl alcohol	16928	-4.8664	-5.5	-5.1832	0.44802286
4-Vinyl guaiacol	332	-4.7662	-5.6	-5.1831	0.58958563
1-Hydroxy-1-(4-hydroxyphenyl)-2-propanone	10261435	-4.7305	-5.6	-5.16525	0.61482935
Cinnamide	5273472	-4.4147	-5.9	-5.15735	1.0502657
α- Terpineol	17100	-4.7087	-5.6	-5.15435	0.63024427
Cinnamic acid	444539	-4.4	-5.9	-5.15	1.06066017
p-coumaric acid	637542	-4.5185	-5.7	-5.10925	0.83544666
Bornyl acetate	6448	-4.464	-5.7	-5.082	0.87398398
Linalool	6549	-4.8625	-5.3	-5.08125	0.30935922
Oxime- methoxy-phenyl	9602988	-4.609	-5.5	-5.0545	0.63003214
Naphthalene	931	-4.0044	-6.1	-5.0522	1.48181297
Indole-3-carboxaldehyde	10256	-4.1429	-5.9	-5.02145	1.24245733
Salicylic alcohol	5146	-4.1962	-5.8	-4.9981	1.13405786
Vanillic acid	8468	-4.5874	-5.4	-4.9937	0.57459497
Decanal	8175	-4.9718	-4.9	-4.9359	0.05077027
Hydroxycinnamaldehyde	71407359	-4.2696	-5.6	-4.9348	0.94073486
Synephrine	7172	-4.5609	-5.3	-4.93045	0.52262262
2-Pentylfuran	19602	-4.9606	-4.9	-4.9303	0.04285067
Phthalic acid	1017	-4.1366	-5.7	-4.9183	1.10549074
Decan-2-one	12741	-5.1194	-4.7	-4.9097	0.29656058
n-acetyl-L-glutamine	182230	-4.7064	-5.1	-4.9032	0.27831723
2, 6,-Nonadienal, 3, 7-dimethyl	5364526	-5.2967	-4.4	-4.84835	0.63406265

2,4,6-Trimethyl-5H-1,3,5-dithiazine	12518	-3.8607	-5.8	-4.83035	1.37129218
2-(1-Pentenyl)furan	5369956	-4.6584	-5	-4.8292	0.24154768
Scopoletin	5280460	-4.8319	-4.8	-4.81595	0.02255671
p-Hydroxy-acetophenone	7469	-4.3131	-5.3	-4.80655	0.69784368
m-Hydroxy-acetophenone	8487	-4.307	-5.3	-4.8035	0.70215703
2,6-Dimethoxy-hydroquinone	96038	-4.594	-5	-4.797	0.28708535
Anthranilic acid methyl ester	8635	-4.438	-5.1	-4.769	0.46810469
4-deoxypyridoxine	6094	-4.5186	-4.9	-4.7093	0.26969053
Gallic acid	370	-4.1138	-5.3	-4.7069	0.83877006
Acetophenone	7410	-4.207	-5.2	-4.7035	0.70215703
Tyrosol	10393	-4.4069	-5	-4.70345	0.41938503
Gentisic acid	3469	-3.985	-5.4	-4.6925	1.0005561
1,6-dioxaspiro[4.4]non-3-ene	10374471	-4.4645	-4.9	-4.68225	0.307945
Benzofuran, 2,3,-dihydro	10329	-4.2513	-5.1	-4.67565	0.60012153
3,4-Dihydroxybenzoic acid	72	-4.0344	-5.3	-4.6672	0.89491434
Nonanol	8914	-4.826	-4.5	-4.663	0.23051681
Anisaldehyde	31244	-4.2319	-5	-4.61595	0.54312872
Dimethyl-L-tartrate	11851	-4.4985	-4.7	-4.59925	0.14248202
Glucosamine	439213	-4.3604	-4.8	-4.5802	0.31084414
3-Octanone	246728	-4.6443	-4.5	-4.57215	0.10203551
Indole	798	-4.024	-5.1	-4.562	0.7608469
DL-a aminopimelic acid	101122	-4.4709	-4.6	-4.53545	0.09128749
n-Octanal	454	-4.6303	-4.4	-4.51515	0.16284669
Citric acid	311	-4.1841	-4.8	-4.49205	0.43550707
2-Heptanone	8051	-4.7635	-4.2	-4.48175	0.39845467
L-2-aminoadipic acid	92136	-4.463	-4.5	-4.4815	0.02616295
p-hydroxybenzoic acid	135	-3.9569	-5	-4.47845	0.73758308
Camphor	2537	-3.7481	-5.2	-4.47405	1.02664834
Mannitol	6251	-4.5451	-4.4	-4.47255	0.10260119
Borneol	64685	-3.9352	-5	-4.4676	0.7529273
5,6-Dihydro-4-pentyl- 2,6-dimethyl-4H-1,3,5-dithiazine	528360	-4.4324	-4.5	-4.4662	0.04780042
Resorcinol	5054	-3.6963	-5.2	-4.44815	1.06327647
p-Hydroxybenzyl alcohol	125	-4.167	-4.7	-4.4335	0.37688791
Nonanal	31289	-4.5463	-4.3	-4.42315	0.1741604
Levoglucosan	2724705	-4.0292	-4.8	-4.4146	0.54503791
3-hydroxy-2,3 dihydromaltol	119838	-4.0065	-4.8	-4.40325	0.56108923
Heptanal	8130	-4.5712	-4.2	-4.3856	0.26247804

Galactal	2734735	-4.1197	-4.6	-4.35985	0.33962339
p-Hydroxy-benzaldehyde	126	-3.9481	-4.7	-4.32405	0.53167359
Pyrogallol	1057	-3.7576	-4.8	-4.2788	0.73708811
Niacinamide	936	-3.8435	-4.7	-4.27175	0.60563696
Leucine	6106	-4.0361	-4.5	-4.26805	0.32802684
Rhamnose	25310	-3.9858	-4.5	-4.2429	0.36359431
adenine	190	-3.7562	-4.7	-4.2281	0.66736738
Benzaldehyde	240	-3.7272	-4.6	-4.1636	0.6171628
Arabitol	94154	-4.1734	-4.1	-4.1367	0.05190164
Levulinic acid	11579	-4.0863	-4.1	-4.09315	0.00968736
Glutaric acid	743	-4.0702	-4.1	-4.0851	0.02107178
Proline	145742	-3.966	-4.2	-4.083	0.16546299
Hexanal	6184	-4.3069	-3.8	-4.05345	0.35843243
Creatinine	588	-3.8957	-4.2	-4.04785	0.21517259
Histidinol	776	-3.8791	-4.2	-4.03955	0.22691057
1,4-benzoquinone	4650	-3.4607	-4.6	-4.03035	0.80560676
2-acetylpyrrole	14079	-3.9207	-4.1	-4.01035	0.12678425
6-azacytosine	70265	-3.8149	-4.2	-4.00745	0.27230682
2-deoxy-D-ribose	5460005	-3.9422	-4	-3.9711	0.04087077
Succinic acid	1110	-3.6622	-4	-3.8311	0.23886067
L-threonine	6288	-3.7189	-3.8	-3.75945	0.05734636
Aminobutyric acid	119	-3.7508	-3.6	-3.6754	0.1066317
3,5-Dimethyl-1,2,4-trithiolane	32033	-4.0116	-3.3	-3.6558	0.50317719
1, 2, 3-Butanetriol	20497	-3.7666	-3.4	-3.5833	0.25922535
Choline	305	-3.8847	-3.1	-3.49235	0.55486669
Formic acid	284	-2.7725	-2.7	-2.73625	0.05126524

Table S3. UD Phytochemicals docking values against CLR1. Values in kcal/mol.

Phytochemical	PubChem CID	Moe Result	AutoVina Result	Mean value	Deviation
Zafirlukast	5717	-9.2853	-13.3	-11.29265	2.83882159
Amentoflavone	5281600	-7.7306	-11.4	-9.5653	2.59465762
Lutein	5281243	-8.8226	-9.5	-9.1613	0.47899413
Alpha-tocotrienol	5282347	-8.8163	-9.4	-9.10815	0.41273823
Violaxanthin	448438	-8.3911	-9.5	-8.94555	0.78411071
Epicatechin gallate	107905	-7.6778	-10.2	-8.9389	1.78346472
Epigallocatechin gallate	65064	-7.8464	-10	-8.9232	1.52282516
β-carotene	5280489	-8.2492	-9.5	-8.8746	0.88444916
Neoxanthin	5282217	-7.845	-9.9	-8.8725	1.45310444
Sitosterol-β-D-glucoside	91884650	-7.9921	-9.5	-8.74605	1.06624632

Peonidin 3-O-rutinoside	44256842	-7.6581	-9.3	-8.47905	1.16099862
Kaempferol-3-rutinoside	5318767	-7.5365	-9.3	-8.41825	1.24698281
Isorhamnetin 3-O-rutinoside	5481663	-7.847	-8.9	-8.3735	0.74458344
9-amino-camptothecin	72402	-6.5824	-10	-8.2912	2.41660814
Quercetin rhamnoside	15939939	-7.3642	-9.2	-8.2821	1.29810663
Apigenin-7-O-glucoside	44257792	-7.3268	-9.2	-8.2634	1.32455242
Dicaffeoylquinic acid	12358846	-6.8071	-9.7	-8.25355	2.04558921
Cholecalciferol	5280795	-6.8615	-9.5	-8.18075	1.86570124
Hecogenin	91453	-5.8181	-10.3	-8.05905	3.16918188
Isorhamnetin-3-O-glucoside	5318645	-6.9907	-9.1	-8.04535	1.49150033
7α-Hydroxy sitosterol	161816	-6.8783	-9	-7.93915	1.50026846
Quercitrin	5280459	-6.5394	-9.3	-7.9197	1.95203898
Isorhamnetin rutinoside	133562525	-7.8337	-8	-7.91685	0.11759186
7β-Hydroxy-sitosterol	12309569	-6.5349	-9.2	-7.86745	1.88451028
Phytosterols	12303662	-6.7343	-9	-7.86715	1.60209183
(+)-Neo-olivil	9976812	-7.1292	-8.5	-7.8146	0.96930198
Deoxyharringtonine	285342	-7.3819	-8.1	-7.74095	0.50777338
Caffeoyl feruloyl tartaric acid	129724266	-7.7648	-7.7	-7.7324	0.04582052
Quercetin dihexoside	5320835	-7.3235	-8.1	-7.71175	0.54906842
Quercetin-3-O-glucoside	25203368	-6.7144	-8.7	-7.7072	1.40403122
Solanidine	65727	-5.2102	-10.2	-7.7051	3.52832142
Carnosol	442009	-6.0237	-9.3	-7.66185	2.31669395
Kaempferol rhamnoside	5835713	-6.4036	-8.9	-7.6518	1.76522137
Bioallethrin	15558638	-6.8256	-8.4	-7.6128	1.11326892
γ-sitosterol	457801	-5.9077	-9.3	-7.60385	2.39871833
Pukateine	442340	-5.9049	-9.3	-7.60245	2.40069823
Kaempferol-3-O-glucoside	5282102	-6.4301	-8.6	-7.51505	1.534351
Beta-Sitosterol	222284	-5.9227	-9.1	-7.51135	2.24669038
3,4-dimethoxychalcone	5354494	-6.3834	-8.6	-7.4917	1.56737289
6-aminochrysene	17534	-5.7364	-9.2	-7.4682	2.44913505
Chlorogenic acid	1794427	-6.307	-8.6	-7.4535	1.62139585
Isolariciresinol	160521	-7.2816	-7.6	-7.4408	0.2251428
Catechin hydrate	107957	-6.2596	-8.6	-7.4298	1.65491271
Quercetin-3-glucoside	5280804	-6.3331	-8.5	-7.41655	1.53222968
Epicatechin	72276	-6.1085	-8.6	-7.35425	1.76175655
10-gingerdione	5317591	-7.2815	-7.4	-7.34075	0.08379215
Convolvamine	420422	-6.5791	-8.1	-7.33955	1.0754387
Estra-1,3,5(10)-trien-17B-ol	9811784	-6.1295	-8.5	-7.31475	1.67619662
Incensole oxide acetate	73755086	-6.2194	-8.4	-7.3097	1.54191705
Piperine	638024	-6.1118	-8.5	-7.3059	1.68871241

Kaempferol pentoside	14749097	-6.3891	-8.2	-7.29455	1.28049967
Ursolic acid	64945	-5.9873	-8.6	-7.29365	1.84745789
O-Feruloyl quinic acid	10177048	-6.2794	-8.3	-7.2897	1.42877996
Isorhamnetin	5281654	-6.35	-8.2	-7.275	1.30814755
Crysoeriol	5280666	-6.311	-8.2	-7.2555	1.33572471
Catechin	9064	-5.8818	-8.6	-7.2409	1.92205765
1,2-Diguaiacyl-1,3-propanediol	6426042	-6.7305	-7.7	-7.21525	0.68554002
Quercetin	5280343	-6.027	-8.4	-7.2135	1.67796439
Neochlorogenic acid	5280633	-6.4101	-8	-7.20505	1.12422907
4-O-Caffeoylquinic acid	58427569	-6.097	-8.3	-7.1985	1.55775624
Integerrimine	5281733	-5.8432	-8.5	-7.1716	1.8786413
Luteolin	5280445	-5.7869	-8.5	-7.14345	1.91845141
Apoatropine	64695	-5.7415	-8.5	-7.12075	1.95055406
Incensole oxide	90470329	-6.1318	-8.1	-7.1159	1.39172757
8-dehydrogingerdione	131752598	-6.8292	-7.4	-7.1146	0.40361655
Benzyl salicilate	8363	-5.8887	-8.3	-7.09435	1.70504658
Myricetin	5281672	-6.0788	-8.1	-7.0894	1.42920423
Caffeoylquinic acid	10155076	-6.17	-8	-7.085	1.29400541
Kaempferol	5280863	-5.9664	-8.1	-7.0332	1.50868303
Feruloyl malate	71694479	-6.3272	-7.7	-7.0136	0.97071619
Neophytadiene	10446	-7.4045	-6.6	-7.00225	0.56886741
Dihydrokavain	10220256	-5.8987	-8.1	-6.99935	1.55655416
Phytol	5280435	-7.0931	-6.9	-6.99655	0.13654232
Genistein	5280961	-5.7681	-8.2	-6.98405	1.71961298
Caffeoylmalic acid	6124299	-6.1395	-7.8	-6.96975	1.17415081
Safranal	61041	-4.5988	-9.3	-6.9494	3.3242504
Farnesylacetone	1711945	-6.891	-7	-6.9455	0.07707464
Naringenin	932	-5.8755	-8	-6.93775	1.50224836
Osthole	10228	-5.8732	-8	-6.9366	1.5038747
Flavan	94156	-5.6699	-8.2	-6.93495	1.78905087
Apigenin	5280443	-5.6551	-8.2	-6.92755	1.79951605
Flavone	10680	-5.4522	-8.4	-6.9261	2.08440937
Eleutheroside B	5316860	-6.4517	-7.4	-6.92585	0.67054936
[1,1-Bicyclopropyl-2-octanoic acid 2hexyl-methyl ester	50930793	-7.2434	-6.6	-6.9217	0.4549525
6-benzylaminopurine	62389	-5.3864	-8.4	-6.8932	2.130937
Arachidonic acid	444899	-7.2534	-6.5	-6.8767	0.53273425
Podocarpic acid	93017	-5.3012	-8.4	-6.8506	2.19118249
Anthocyanins	145858	-5.3574	-8.3	-6.8287	2.08073241
β-Bisabolene	10104370	-6.1857	-7.4	-6.79285	0.85863976
α-Copaene	442355	-5.5702	-8	-6.7851	1.71812806
Isopilosine	72312	-5.9309	-7.6	-6.76545	1.18023193

Secoisolariciresinol	65373	-6.7207	-6.8	-6.76035	0.05607357
Kavain	5281565	-5.4879	-8	-6.74395	1.77632295
p-Coumaroylmalic acid	129720114	-6.194	-7.2	-6.697	0.71134942
δ-Cadinene	441005	-5.7857	-7.6	-6.69285	1.28290383
Pterostilbene	5281727	-6.159	-7.2	-6.6795	0.73609816
β - Vetivenene	14475467	-5.3038	-8	-6.6519	1.9065013
9-Hydroxy-10,12-octadecadienoic acid	1927	-6.739	-6.5	-6.6195	0.16899852
Homatropine	5282593	-5.6374	-7.6	-6.6187	1.38776777
4-shogaol	9794897	-6.414	-6.8	-6.607	0.27294322
Dibutyl phthalate	3026	-6.3947	-6.8	-6.59735	0.28659038
Quinic acid	6508	-4.1625	-9	-6.58125	3.42062905
Caffeoyl tartaric acid	9857913	-5.8601	-7.3	-6.58005	1.01816305
Farnesol	445070	-6.4383	-6.7	-6.56915	0.18504984
ar-Curcumene	92139	-5.7118	-7.4	-6.5559	1.19373767
Linolenic acid	5280934	-6.8907	-6.2	-6.54535	0.48839865
Heptadecanoic acid	10465	-7.2784	-5.8	-6.5392	1.04538667
β-Selinene	442393	-4.9756	-8.1	-6.5378	2.20928443
β - Sesquiphellandrene	519764	-5.8329	-7.2	-6.51645	0.96668568
Methyl palmitate	8181	-7.312	-5.7	-6.506	1.13985613
Hexahydrofarnesylacetone	10408	-6.2949	-6.7	-6.49745	0.28644896
α-Selinene	10856614	-5.2572	-7.7	-6.4786	1.72732045
Calamenene	6429077	-5.2454	-7.7	-6.4727	1.73566431
γ-Cadinene	92313	-5.4364	-7.5	-6.4682	1.45918555
cis-10-Heptadecenoic acid	5312435	-7.1097	-5.8	-6.45485	0.92609775
Rutin	5280805	-7.6876	-5.2	-6.4438	1.75899883
Adenosine	60961	-6.0808	-6.8	-6.4404	0.5085512
β-Bourbonene	62566	-5.2343	-7.6	-6.41715	1.67280251
Abscisic acid	5375199	-5.4986	-7.3	-6.3993	1.27378216
1,2-Benzenedicarboxylic acid	90531	-5.7572	-7	-6.3786	0.87879231
Shikimic acid	8742	-4.851	-7.9	-6.3755	2.15596858
4-methyl-7-ethoxycoumarin	66595	-5.529	-7.2	-6.3645	1.18157543
Isotachioside	15098566	-5.6689	-7	-6.33445	0.94122984
Tachioside	11962143	-5.66723	-7	-6.333615	0.9424107
3-Oxo-a-ionol	5370052	-5.654	-7	-6.327	0.95176573
Caryophyllene oxide	1742210	-5.2089	-7.4	-6.30445	1.54934167
Isopropyl dodecanoate	25068	-6.5985	-6	-6.29925	0.42320341
Harmine	5280953	-5.4662	-7.1	-6.2831	1.15527106
Palmitic acid	985	-6.8227	-5.7	-6.26135	0.79386878
(E)-Geranyl acetone	1713001	-5.9057	-6.6	-6.25285	0.49094424

4-(4-Hydroxy-2,6,6-trimethyl1-cyclohexen-1-yl)-3-buten2-one	538953	-5.3783	-7.1	-6.23915	1.21742575
4-(3-Hydroxy-1-butyn-1-yl)- 3,5,5-trimethyl-2-cyclohexen-1-ol	5280654	-5.4326	-7	-6.2163	1.10831917
α- Copaene-8-ol	25086830	-5.2963	-7.1	-6.19815	1.2754085
Silane, triethyl(2-phenylethoxy)	610043	-6.2938	-6.1	-6.1969	0.13703729
Palmitoleic acid	445638	-6.2623	-6.1	-6.18115	0.11476343
Phosphatidylcholine	10425706	-6.4498	-5.9	-6.1749	0.38876731
Arbutine	440936	-5.6321	-6.7	-6.16605	0.75511933
2H-Indeno[1,2-b]furan-2-one, 3,3a,4,5,6,7,8,8b-octahydro-8,8-dimethyl	605626	-4.6997	-7.6	-6.14985	2.0508218
α-Humulene	5281520	-5.2839	-7	-6.14195	1.21346595
Geranyl acetone	1549778	-5.6472	-6.6	-6.1236	0.67373134
Carvacryl acetate	80792	-5.3345	-6.9	-6.11725	1.10697567
β-Ionone	638014	-4.9986	-7.2	-6.0993	1.55662487
4-aminoantipyrine	2151	-5.2789	-6.9	-6.08945	1.1462908
β-Caryophyllene	5281515	-5.2715	-6.9	-6.08575	1.15152339
Myristoleic acid	5281119	-6.3248	-5.8	-6.0624	0.37108964
3-Hydroxy-damascone	5366075	-5.1143	-7	-6.05715	1.33339126
α-Ionone	5282108	-5.0821	-7	-6.04105	1.3561601
α- Terpinyl acetate	111037	-5.5464	-6.5	-6.0232	0.67429703
4-methylmethoxycoumarin	223821	-5.0446	-7	-6.0223	1.3826766
Hydrocotarnine	3646	-4.9893	-7	-5.99465	1.4217796
Carvylacetate	7335	-5.2878	-6.7	-5.9939	0.9985762
Myristic acid	11005	-6.0784	-5.9	-5.9892	0.12614785
Harmol	68094	-4.3978	-7.5	-5.9489	2.19358666
Umbelliferone	5281426	-4.69	-7.2	-5.945	1.77483802
1-Methyl naphthalene	7002	-4.8778	-7	-5.9389	1.50062201
Cinnamyl acetate	5282110	-5.1648	-6.7	-5.9324	1.08555033
2, 6,-Nonadienal, 3, 7-dimethyl	5364526	-5.54549	-6.3	-5.922745	0.53351914
α-Longipinene	520957	-4.8301	-7	-5.91505	1.534351
Ferulic acid	445858	-5.3636	-6.4	-5.8818	0.73284547
3,4-Dimethyl-5-pentylfuran-2(5H)-one	13192443	-5.1969	-6.5	-5.84845	0.92143085
Methylcoumarin	17130	-4.6881	-7	-5.84405	1.63476017
Sinapic acid	637775	-5.3841	-6.3	-5.84205	0.6476391
3,4-Dimethyl-5-pentylidene-2(5H)-furanone	6433214	-5.4503	-6.2	-5.82515	0.53011795

Dodecendioic acid	5283028	-5.7963	-5.8	-5.79815	0.0026163
Diphenyl	7095	-4.7317	-6.8	-5.76585	1.46250896
Sinapyl alcohol	5280507	-5.6316	-5.9	-5.7658	0.18978746
Coniferol	1549095	-5.4224	-6.1	-5.7612	0.47913555
Caffeic acid	689043	-4.8748	-6.6	-5.7374	1.21990062
DL-methyl-m-tyrosine	2110	-4.9635	-6.5	-5.73175	1.08646957
1-Dodecanamine, N, N-dimethyl	8168	-6.012	-5.4	-5.706	0.43274935
Esculetin	5281416	-4.5055	-6.8	-5.65275	1.62245651
Eugenol	3314	-4.7999	-6.5	-5.64995	1.20215224
Naphthalene	931	-4.5981	-6.7	-5.64905	1.48626774
Isopropyl-β-D-thio-galacto-pyranoside	656894	-5.3978	-5.9	-5.6489	0.35510903
Linalool	6549	-5.4829	-5.8	-5.64145	0.22422356
2(3H)-Naphthalenone, 4, 4a,5,6,7,8- hexahydro-1-methoxy	534313	-4.6344	-6.6	-5.6172	1.38988909
Carvone	7439	-4.9276	-6.3	-5.6138	0.97043335
Lauric acid	3893	-5.607	-5.6	-5.6035	0.00494975
Syringic acid	10742	-4.848	-6.3	-5.574	1.02671905
Lotaustralin	441467	-5.1148	-6	-5.5574	0.62593092
1,2-O-isopropylidene-D-glucoside	87704	-5.1619	-5.9	-5.53095	0.52191552
1-Hydroxy-1-(4-hydroxyphenyl)-2-propanone	10261435	-4.8377	-6.2	-5.51885	0.96329157
Bornyl acetate	6448	-5.0294	-6	-5.5147	0.68631784
Phenylalanine	6140	-4.7217	-6.3	-5.51085	1.11602663
Methyl eugenol	7127	-5.1103	-5.9	-5.50515	0.55840223
Salicylic alcohol	5146	-4.5095	-6.5	-5.50475	1.40749605
Carvacrol	10364	-4.7086	-6.3	-5.5043	1.12528973
Cinnamide	5273472	-4.6028	-6.4	-5.5014	1.27081231
4-Acetyl-2-methylphenol	70135	-4.5748	-6.4	-5.4874	1.2906113
p-coumaric acid	637542	-4.7428	-6.2	-5.4714	1.030396
α- Terpineol	17100	-4.6997	-6.2	-5.44985	1.0608723
4-Vinyl guaiacol	332	-4.4953	-6.4	-5.44765	1.34682629
Vitamin B5	6613	-5.1096	-5.7	-5.4048	0.41747584
Scopoletin	5280460	-4.7967	-6	-5.39835	0.85086159
β-Homocyclocitral	61124	-4.6758	-6.1	-5.3879	1.00706148
Oxime- methoxy-phenyl	9602988	-4.6698	-6.1	-5.3849	1.01130412
Cinnamic acid	444539	-4.5333	-6.2	-5.36665	1.17853487
Anthranilic acid methyl ester	8635	-4.4225	-6.3	-5.36125	1.32759298
Indole-3-carboxaldehyde	10256	-4.4857	-6.2	-5.34285	1.21219315

β-Cyclocitral	9895	-4.5498	-6.1	-5.3249	1.09615693
Hydroxycinnamaldehyde	71407359	-4.4329	-6.2	-5.31645	1.24952839
Decanal	8175	-5.4307	-5.2	-5.31535	0.16312953
Synephrine	7172	-4.7279	-5.9	-5.31395	0.82879986
Homovanillyl alcohol	16928	-4.6054	-6	-5.3027	0.98613112
p-Cymene	7463	-4.4679	-6.1	-5.28395	1.15406898
Tyrosol	10393	-4.6473	-5.9	-5.27365	0.88579266
Thymol	6989	-4.835	-5.7	-5.2675	0.61164737
m-Hydroxy-acetophenone	8487	-4.4182	-6.1	-5.2591	1.18921218
p-Hydroxy-acetophenone	7469	-4.3179	-6.2	-5.25895	1.33084567
2-(1-Pentenyl)furan	5369956	-4.8157	-5.7	-5.25785	0.62529453
Vanillic acid	8468	-4.3035	-6.2	-5.25175	1.34102801
n-acetyl-L-glutamine	182230	-4.861	-5.6	-5.2305	0.52255191
2-Pentylfuran	19602	-4.9487	-5.5	-5.22435	0.38982797
Cumin aldehyde	326	-4.4233	-6	-5.21165	1.11489526
p-Hydroxybenzyl alcohol	125	-4.7761	-5.6	-5.18805	0.58258528
Carnosine	439224	-4.5383	-5.8	-5.16915	0.89215663
(E)-Anethole	637563	-4.7357	-5.6	-5.16785	0.61115239
Gallic acid	370	-4.3235	-6	-5.16175	1.18546452
3,4-Dihydroxybenzoic acid	72	-4.32	-6	-5.16	1.18793939
Benzofuran, 2,3,-dihydro	10329	-4.4133	-5.9	-5.15665	1.05125565
Citric acid	311	-4.6096	-5.7	-5.1548	0.77102923
Indole	798	-4.3773	-5.9	-5.13865	1.0767115
Camphor	2537	-4.4695	-5.8	-5.13475	0.94080557
Decan-2-one	12741	-5.1492	-5.1	-5.1246	0.03478965
Acetophenone	7410	-4.3399	-5.9	-5.11995	1.10315729
Gentisic acid	3469	-4.0272	-6.2	-5.1136	1.53640161
4-deoxypyridoxine	6094	-4.615	-5.6	-5.1075	0.69650018
p-hydroxybenzoic acid	135	-4.2109	-6	-5.10545	1.26508474
DL-a aminopimelic acid	101122	-4.9943	-5.1	-5.04715	0.07474119
Methyl chavicol	8815	-4.5862	-5.5	-5.0431	0.64615418
p-Hydroxy-benzaldehyde	126	-4.1577	-5.9	-5.02885	1.23199214
Nonanol	8914	-5.1383	-4.9	-5.01915	0.16850355
Rhamnose	25310	-4.6346	-5.4	-5.0173	0.54121953
3-Octanone	246728	-4.8074	-5.1	-4.9537	0.20689944
Glucosamine	439213	-4.4778	-5.4	-4.9389	0.65209387
Phthalic acid	1017	-3.9633	-5.9	-4.93165	1.3694537
2,6-Dimethoxy-hydroquinone	96038	-4.5657	-5.2	-4.88285	0.44851783
Niacinamide	936	-4.4572	-5.3	-4.8786	0.5959496
Borneol	64685	-4.2107	-5.5	-4.85535	0.91167277
Levoglucosan	2724705	-4.3066	-5.4	-4.8533	0.77315055
Nonanal	31289	-4.7976	-4.9	-4.8488	0.07240773

Pyrogallol	1057	-4.1663	-5.5	-4.83315	0.94306831
Galactal	2734735	-4.6378	-5	-4.8189	0.25611408
Leucine	6106	-4.5448	-5	-4.7724	0.32187501
Resorcinol	5054	-4.0235	-5.5	-4.76175	1.04404316
1,4-benzoquinone	4650	-4.0189	-5.5	-4.75945	1.04729585
adenine	190	-3.6127	-5.9	-4.75635	1.61736534
L-2-aminoadipic acid	92136	-4.3873	-5.1	-4.74365	0.503955
n-Octanal	454	-4.7779	-4.7	-4.73895	0.05508362
Dimethyl-L-tartrate	11851	-4.5738	-4.9	-4.7369	0.23065823
Anisaldehyde	31244	-4.2553	-5.2	-4.72765	0.66800378
1,6-dioxaspiro[4.4]non-3-ene	10374471	-4.3445	-5.1	-4.72225	0.53421917
Benzaldehyde	240	-3.918	-5.5	-4.709	1.11864293
5,6-Dihydro-4-pentyl- 2,6-dimethyl-4H-1,3,5-dithiazine	528360	-4.4817	-4.9	-4.69085	0.29578277
Mannitol	6251	-4.4562	-4.8	-4.6281	0.24310331
3-hydroxy-2,3 dihydromaltol	119838	-3.9397	-5.2	-4.56985	0.89116668
2-acetylpyrrole	14079	-4.2283	-4.9	-4.56415	0.47496362
2-Heptanone	8051	-4.5125	-4.6	-4.55625	0.06187184
Arabitol	94154	-4.5899	-4.4	-4.49495	0.13427958
Histidinol	776	-4.152	-4.8	-4.476	0.45820519
6-azacytosine	70265	-3.813	-5.1	-4.4565	0.91004643
Heptanal	8130	-4.4708	-4.4	-4.4354	0.05006316
Proline	145742	-3.7627	-5.1	-4.43135	0.9456139
2,4,6-Trimethyl-5H-1,3,5-dithiazine	12518	-4.0916	-4.7	-4.3958	0.43020377
2-deoxy-D-ribose	5460005	-4.4577	-4.3	-4.37885	0.11151074
Levulinic acid	11579	-4.1453	-4.6	-4.37265	0.32152145
Creatinine	588	-3.842	-4.9	-4.371	0.74811897
L-threonine	6288	-3.9374	-4.8	-4.3687	0.60995031
Glutaric acid	743	-4.1397	-4.5	-4.31985	0.25477057
1, 2, 3-Butanetriol	20497	-4.1191	-4.4	-4.25955	0.19862629
Succinic acid	1110	-3.9619	-4.5	-4.23095	0.38049416
Hexanal	6184	-4.0387	-4.3	-4.16935	0.184767
Aminobutyric acid	119	-3.9181	-4.3	-4.10905	0.27004408
3,5-Dimethyl-1,2,4-trithiolane	32033	-4.3859	-3.7	-4.04295	0.48500454
Choline	305	-4.0584	-3.7	-3.8792	0.25342707
Formic acid	284	-3.143	-2.5	-2.8215	0.45466966

Table S4. UD Phytochemicals docking values against CRTH2. Values in kcal/mol.

Phytochemical	PubChem CID	Moe Result	AutoVina Result	Mean value	Deviation
Neoxanthin	5282217	-9.1584	-9.9	-9.5292	0.52439039
Alpha-tocotrienol	5282347	-8.8178	-9.7	-9.2589	0.6238096
Fevipiprant	23582412	-7.6147	-10.8	-9.20735	2.25234723
Ursolic acid	64945	-5.6173	-12.3	-8.95865	4.72538249
Integerrimine	5281733	-6.8197	-11	-8.90985	2.95591848
Amentoflavone	5281600	-7.9604	-9.8	-8.8802	1.30079363
β-carotene	5280489	-9.0361	-8.7	-8.86805	0.23765859
9-amino-camptothecin	72402	-7.4075	-10.3	-8.85375	2.04530636
Hecogenin	91453	-7.1059	-10.6	-8.85295	2.4707018
Kaempferol rhamnoside	5835713	-7.8557	-9.7	-8.77785	1.30411704
Cholecalciferol	5280795	-7.71	-9.7	-8.705	1.40714249
Solanidine	65727	-5.7075	-11.7	-8.70375	4.23733739
Isorhamnetin 3-O-rutinoside	5481663	-8.4971	-8.9	-8.69855	0.28489332
Isorhamnetin rutinoside	133562525	-7.6415	-9.6	-8.62075	1.38486863
Isorhamnetin-3-O-glucoside	5318645	-8.4237	-8.8	-8.61185	0.26608428
Epicatechin gallate	107905	-7.6214	-9.6	-8.6107	1.39908148
Beta-Sitosterol	222284	-7.2732	-9.9	-8.5866	1.85742809
Phytosterols	12303662	-7.273	-9.9	-8.5865	1.85756951
Deoxyharringtonine	285342	-7.3694	-9.8	-8.5847	1.71869374
Pukateine	442340	-6.7473	-10.4	-8.57365	2.58284894
Peonidin 3-O-rutinoside	44256842	-7.6339	-9.4	-8.51695	1.24882129
Dicaffeoylquinic acid	12358846	-7.6238	-9.4	-8.5119	1.25596306
Kaempferol-3-O-glucoside	5282102	-7.7047	-9.3	-8.50235	1.12804745
7β-Hydroxy-sitosterol	12309569	-7.665	-9.3	-8.4825	1.15611959
Quercetin-3-glucoside	5280804	-7.5061	-9.4	-8.45305	1.33918953
Apigenin-7-O-glucoside	44257792	-7.8923	-9	-8.44615	0.78326218
7α-Hydroxy sitosterol	161816	-7.4905	-9.4	-8.44525	1.3502204
(+)-Neo-olivil	9976812	-8.2725	-8.5	-8.38625	0.16086679
Kaempferol pentoside	14749097	-7.3373	-9.4	-8.36865	1.45854916
$_{\gamma}$-sitosterol	457801	-7.2121	-9.5	-8.35605	1.6177896
Epigallocatechin gallate	65064	-7.3109	-9.4	-8.35545	1.47721678
Quercetin-3-O-glucoside	25203368	-7.062	-9.6	-8.331	1.79463701
Incensole oxide	90470329	-7.3586	-9.3	-8.3293	1.3727771
Bioallethrin	15558638	-7.5291	-9.1	-8.31455	1.11079404
10-gingerdione	5317591	-8.7559	-7.8	-8.27795	0.67592337
Carnosol	442009	-6.5472	-10	-8.2736	2.44149829
Quercetin dihexoside	5320835	-7.4724	-9	-8.2362	1.08017632

Quercetin rhamnoside	15939939	-7.3316	-9.1	-8.2158	1.25044763
Sitosterol-β-D-glucoside	91884650	-7.3295	-9.1	-8.21475	1.25193256
Violaxanthin	448438	-8.2965	-8.1	-8.19825	0.13894648
Quercitrin	5280459	-7.4079	-8.9	-8.15395	1.05507403
Kaempferol-3-rutinoside	5318767	-7.4941	-8.8	-8.14705	0.92341075
Chlorogenic acid	1794427	-7.4258	-8.8	-8.1129	0.97170614
Incensole oxide acetate	73755086	-6.9897	-9.1	-8.04485	1.49220744
8-dehydrogingerdione	131752598	-7.6329	-8.4	-8.01645	0.54242161
Caffeoyl feruloyl tartaric acid	129724266	-7.5822	-8.4	-7.9911	0.57827193
Arachidonic acid	444899	-8.4598	-7.5	-7.9799	0.67868109
[1,1-Bicyclopropyl-2-octanoic acid 2hexyl-methyl ester	50930793	-8.2349	-7.7	-7.96745	0.37823142
Eleutheroside B	5316860	-8.0566	-7.8	-7.9283	0.1814436
Isolariciresinol	160521	-7.7516	-8.1	-7.9258	0.246356
4-O-Caffeoylquinic acid	58427569	-7.0148	-8.8	-7.9074	1.26232703
Neochlorogenic acid	5280633	-7.3305	-8.4	-7.86525	0.7562507
Lutein	5281243	-8.8764	-6.8	-7.8382	1.46823652
Phytol	5280435	-8.1492	-7.4	-7.7746	0.5297644
O-Feruloyl quinic acid	10177048	-6.9456	-8.6	-7.7728	1.16983746
6-aminochrysene	17534	-5.689	-9.8	-7.7445	2.90691598
Hexahydrofarnesylacetone	10408	-8.1707	-7.3	-7.73535	0.61567787
Farnesylacetone	1711945	-7.7022	-7.7	-7.7011	0.00155563
Piperine	638024	-6.5656	-8.8	-7.6828	1.57995939
Linolenic acid	5280934	-8.2408	-7.1	-7.6704	0.80666742
Neophytadiene	10446	-7.9142	-7.4	-7.6571	0.36359431
Crysoeriol	5280666	-6.3932	-8.8	-7.5966	1.7018646
Naringenin	932	-6.4152	-8.7	-7.5576	1.61559757
Apigenin	5280443	-6.4031	-8.7	-7.55155	1.62415357
1,2-Diguaiacyl-1,3-propanediol	6426042	-7.1314	-7.9	-7.5157	0.54348227
Apoatropine	64695	-6.5304	-8.5	-7.5152	1.39271752
9-Hydroxy-10,12-octadecadienoic acid	1927	-8.4101	-6.6	-7.50505	1.27993398
Estra-1,3,5(10)-trien-17B-ol	9811784	-5.8899	-9.1	-7.49495	2.26988348
Isorhamnetin	5281654	-6.585	-8.4	-7.4925	1.28339881
Caffeoylquinic acid	10155076	-6.5783	-8.4	-7.48915	1.28813642
Kaempferol	5280863	-6.5767	-8.4	-7.48835	1.28926779
Quercetin	5280343	-6.5612	-8.4	-7.4806	1.30022795
Secoisolariciresinol	65373	-7.9543	-7	-7.47715	0.674792
cis-10-Heptadecenoic acid	5312435	-7.9386	-7	-7.4693	0.66369042
Podocarpic acid	93017	-6.0727	-8.8	-7.43635	1.92849232

Quinic acid	6508	-5.2299	-9.6	-7.41495	3.09012734
Genistein	5280961	-6.0202	-8.8	-7.4101	1.96561543
Isopilosine	72312	-6.7078	-8.1	-7.4039	0.98443406
Anthocyanins	145858	-5.6801	-9.1	-7.39005	2.41823448
Flavone	10680	-5.5661	-9.2	-7.38305	2.56955533
Luteolin	5280445	-6.051	-8.7	-7.3755	1.87312586
Osthole	10228	-6.2506	-8.5	-7.3753	1.59056599
Catechin	9064	-6.4598	-8.2	-7.3299	1.23050722
Myricetin	5281672	-6.344	-8.3	-7.322	1.38310086
3,4-dimethoxychalcone	5354494	-6.7336	-7.9	-7.3168	0.82476935
Heptadecanoic acid	10465	-8.0307	-6.6	-7.31535	1.01165767
α-Humulene	5281520	-5.9842	-8.6	-7.2921	1.84964992
Pterostilbene	5281727	-6.7367	-7.8	-7.26835	0.75186664
Flavan	94156	-5.6106	-8.9	-7.2553	2.32595705
Homatropine	5282593	-6.2776	-8.2	-7.2388	1.35934208
Dibutyl phthalate	3026	-6.7578	-7.7	-7.2289	0.66623601
Convolvamine	420422	-6.7529	-7.7	-7.22645	0.66970083
Catechin hydrate	107957	-6.2367	-8.2	-7.21835	1.38826274
Abscisic acid	5375199	-6.5213	-7.9	-7.21065	0.97488812
β-Bisabolene	10104370	-6.3613	-8	-7.18065	1.15873588
Tachioside	11962143	-6.9304	-7.4	-7.1652	0.33205734
γ-Cadinene	92313	-5.9392	-8.3	-7.1196	1.66933769
Dihydrokavain	10220256	-6.3213	-7.9	-7.11065	1.11630948
Calamenene	6429077	-6.0978	-8.1	-7.0989	1.4157692
Feruloyl malate	71694479	-6.6857	-7.5	-7.09285	0.57579705
δ-Cadinene	441005	-5.963	-8.2	-7.0815	1.58179787
Benzyl salicilate	8363	-5.9259	-8.2	-7.06295	1.60803153
ar-Curcumene	92139	-6.1119	-8	-7.05595	1.33508831
α-Selinene	10856614	-5.9081	-8.2	-7.05405	1.62061803
β-Caryophyllene	5281515	-5.7323	-8.3	-7.01615	1.81563808
Methyl palmitate	8181	-7.5954	-6.4	-6.9977	0.84527545
4-shogaol	9794897	-6.585	-7.4	-6.9925	0.57629203
β-Bourbonene	62566	-5.8698	-8.1	-6.9849	1.57698954
β - Vetivenene	14475467	-5.7566	-8.2	-6.9783	1.72774471
Isotachioside	15098566	-6.3482	-7.6	-6.9741	0.88515627
Palmitoleic acid	445638	-7.1472	-6.8	-6.9736	0.24550747
2H-Indeno[1,2-b]furan-2-one, 3,3a,4,5,6,7,8,8b-octahydro-8,8-dimethyl	605626	-5.4818	-8.4	-6.9409	2.06347901
Palmitic acid	985	-7.3703	-6.5	-6.93515	0.61539503
α- Copaene-8-ol	25086830	-5.9479	-7.9	-6.92395	1.38034315
Caryophyllene oxide	1742210	-5.5388	-8.3	-6.9194	1.95246324
Rutin	5280805	-7.9242	-5.9	-6.9121	1.43132555

3-Hydroxy-damascone	5366075	-6.2048	-7.6	-6.9024	0.98655538
β-Selinene	442393	-5.6783	-8.1	-6.88915	1.71240049
p-Coumaroylmalic acid	129720114	-6.3565	-7.4	-6.87825	0.73786593
Caffeoylmalic acid	6124299	-6.354	-7.4	-6.877	0.73963369
Harmine	5280953	-5.7357	-8	-6.86785	1.60110188
α-Copaene	442355	-5.5044	-8.2	-6.8522	1.90607704
Safranal	61041	-4.7847	-8.9	-6.84235	2.90995654
Caffeoyl tartaric acid	9857913	-6.3693	-7.2	-6.78465	0.5873936
Farnesol	445070	-6.6151	-6.9	-6.75755	0.20145472
β - Sesquiphellandrene	519764	-6.0007	-7.5	-6.75035	1.0601652
Geranyl acetone	1549778	-6.2355	-7.2	-6.71775	0.68200449
β-Ionone	638014	-5.9657	-7.4	-6.68285	1.01420326
Kavain	5281565	-5.8216	-7.5	-6.6608	1.18680802
6-benzylaminopurine	62389	-5.5097	-7.8	-6.65485	1.61948666
α-Longipinene	520957	-5.3015	-8	-6.65075	1.90812765
4-(3-Hydroxy-1-butyn-1-yl)- 3,5,5-trimethyl-2-cyclohexen-1-ol	5280654	-6.1756	-7.1	-6.6378	0.65364951
Isopropyl dodecanoate	25068	-6.8426	-6.4	-6.6213	0.31296546
Arbutine	440936	-6.0137	-7.2	-6.60685	0.83884077
Adenosine	60961	-5.8107	-7.4	-6.60535	1.12380481
Myristoleic acid	5281119	-6.6092	-6.6	-6.6046	0.00650538
4-(4-Hydroxy-2,6,6-trimethyl1-cyclohexen-1-yl)-3-buten2-one	538953	-5.9061	-7.3	-6.60305	0.98563614
Myristic acid	11005	-6.8045	-6.4	-6.60225	0.28602469
Carvacryl acetate	80792	-5.8687	-7.3	-6.58435	1.01208194
4-methyl-7-ethoxycoumarin	66595	-5.7922	-7.3	-6.5461	1.0661756
1,2-Benzenedicarboxylic acid	90531	-6.0757	-7	-6.53785	0.6535788
Dodecendioic acid	5283028	-6.7256	-6.3	-6.5128	0.30094465
Harmol	68094	-4.89	-8.1	-6.495	2.26981277
Lotaustralin	441467	-6.1853	-6.8	-6.49265	0.43465854
α-Ionone	5282108	-5.8685	-7.1	-6.48425	0.870802
Carvylacetate	7335	-5.9426	-7	-6.4713	0.74769471
α- Terpinyl acetate	111037	-5.841	-7.1	-6.4705	0.89024744
3-Oxo-a-ionol	5370052	-5.8127	-7.1	-6.45635	0.91025856
Hydrocotarnine	3646	-5.6089	-7.3	-6.45445	1.19578828
(E)-Geranyl acetone	1713001	-5.9025	-6.8	-6.35125	0.63462834
4-aminoantipyrine	2151	-5.5903	-7.1	-6.34515	1.06751911
Phosphatidylcholine	10425706	-6.8767	-5.8	-6.33835	0.76134187
Shikimic acid	8742	-4.6246	-8	-6.3123	2.38676823

4-methylmethoxycoumarin	223821	-5.2558	-7.3	-6.2779	1.44546768
1-Dodecanamine, N, N-dimethyl	8168	-6.5779	-5.9	-6.23895	0.47934769
3,4-Dimethyl-5-pentylidene-2(5H)-furanone	6433214	-5.6012	-6.8	-6.2006	0.84767961
Carnosine	439224	-6.0939	-6.3	-6.19695	0.14573471
1,2-O-isopropylidene-D-glucoside	87704	-5.6552	-6.7	-6.1776	0.73878516
Sinapyl alcohol	5280507	-5.6522	-6.6	-6.1261	0.67019581
3,4-Dimethyl-5-pentylfuran-2(5H)-one	13192443	-5.6353	-6.6	-6.11765	0.68214591
Ferulic acid	445858	-5.3805	-6.8	-6.09025	1.00373808
DL-methyl-m-tyrosine	2110	-5.5675	-6.6	-6.08375	0.73008775
Sinapic acid	637775	-5.419	-6.7	-6.0595	0.90580379
Lauric acid	3893	-6.1082	-6	-6.0541	0.07650895
Diphenyl	7095	-4.8607	-7.2	-6.03035	1.65413489
Cinnamyl acetate	5282110	-5.6393	-6.4	-6.01965	0.53789613
2(3H)-Naphthalenone, 4, 4a,5,6,7,8- hexahydro-1-methoxy	534313	-5.128	-6.9	-6.014	1.25299322
Methylcoumarin	17130	-4.813	-7.2	-6.0065	1.68786389
Bornyl acetate	6448	-5.2107	-6.8	-6.00535	1.12380481
Coniferol	1549095	-5.5219	-6.4	-5.96095	0.62091046
Silane, triethyl(2-phenylethoxy)	610043	-6.3736	-5.5	-5.9368	0.61772848
Vitamin B5	6613	-5.8601	-6	-5.93005	0.09892424
Carvacrol	10364	-5.3572	-6.5	-5.9286	0.80808163
Isopropyl-β-D-thio-galacto-pyranoside	656894	-5.7351	-6.1	-5.91755	0.25802326
Esculetin	5281416	-4.7135	-7.1	-5.90675	1.68751033
Carvone	7439	-5.2277	-6.5	-5.86385	0.89965196
Caffeic acid	689043	-5.0755	-6.6	-5.83775	1.07798429
β-Homocyclocitral	61124	-5.2664	-6.4	-5.8332	0.80157625
Methyl eugenol	7127	-5.4662	-6.2	-5.8331	0.51887496
1-Hydroxy-1-(4-hydroxyphenyl)-2-propanone	10261435	-5.3115	-6.3	-5.80575	0.69897505
p-coumaric acid	637542	-5.0681	-6.5	-5.78405	1.0125062
Cumin aldehyde	326	-5.1216	-6.4	-5.7608	0.90396531
Decanal	8175	-5.7036	-5.8	-5.7518	0.06816509
Synephrine	7172	-5.4506	-6	-5.7253	0.38848447
α- Terpineol	17100	-5.1467	-6.3	-5.72335	0.81550625
Linalool	6549	-5.7636	-5.6	-5.6818	0.11568267

Umbelliferone	5281426	-4.5236	-6.8	-5.6618	1.60965788
Methyl chavicol	8815	-5.3025	-6	-5.65125	0.49320698
Eugenol	3314	-5.3001	-6	-5.65005	0.49490404
Decan-2-one	12741	-5.6493	-5.6	-5.62465	0.03486036
1-Methyl naphthalene	7002	-4.4304	-6.8	-5.6152	1.67556023
Cinnamic acid	444539	-4.7141	-6.5	-5.60705	1.262822
p-Cymene	7463	-4.9746	-6.2	-5.5873	0.86648865
Cinnamide	5273472	-4.845	-6.3	-5.5725	1.02884037
β-Cyclocitral	9895	-4.8427	-6.3	-5.57135	1.03046671
Hydroxycinnamaldehyde	71407359	-4.8899	-6.2	-5.54495	0.92638059
Phenylalanine	6140	-4.8746	-6.2	-5.5373	0.93719933
Thymol	6989	-4.8693	-6.2	-5.53465	0.94094699
Syringic acid	10742	-5.1464	-5.8	-5.4732	0.46216499
Nonanal	31289	-5.5464	-5.4	-5.4732	0.10352043
(E)-Anethole	637563	-4.8433	-6.1	-5.47165	0.88862109
2,4,6-Trimethyl-5H-1,3,5-dithiazine	12518	-4.6411	-6.3	-5.47055	1.17301944
Camphor	2537	-4.626	-6.3	-5.463	1.18369675
2-(1-Pentenyl)furan	5369956	-5.1747	-5.7	-5.43735	0.37144319
4-Acetyl-2-methylphenol	70135	-4.6724	-6.2	-5.4362	1.08017632
n-acetyl-L-glutamine	182230	-5.1643	-5.7	-5.43215	0.3787971
Homovanillyl alcohol	16928	-4.9815	-5.8	-5.39075	0.5787669
DL-a aminopimelic acid	101122	-5.1631	-5.6	-5.38155	0.30893495
Nonanol	8914	-5.3269	-5.4	-5.36345	0.05168951
m-Hydroxy-acetophenone	8487	-4.6801	-6	-5.34005	0.93331024
Borneol	64685	-4.6269	-6	-5.31345	0.97092832
2, 6,-Nonadienal, 3, 7-dimethyl	5364526	-5.6615	-4.9	-5.28075	0.53846181
Vanillic acid	8468	-4.7583	-5.8	-5.27915	0.73659313
2-Pentylfuran	19602	-5.0541	-5.5	-5.27705	0.31529891
Indole-3-carboxaldehyde	10256	-4.5462	-6	-5.2731	1.02799184
Citric acid	311	-5.1082	-5.4	-5.2541	0.20633376
Phthalic acid	1017	-4.5062	-6	-5.2531	1.05627611
Oxime- methoxy-phenyl	9602988	-4.606	-5.9	-5.253	0.91499617
Salicylic alcohol	5146	-4.1527	-6.3	-5.22635	1.51837039
4-Vinyl guaiacol	332	-4.8502	-5.6	-5.2251	0.53018866
Naphthalene	931	-4.247	-6.2	-5.2235	1.38097954
Dimethyl-L-tartrate	11851	-5.3114	-5.1	-5.2057	0.14948237
Scopoletin	5280460	-5.3105	-5.1	-5.20525	0.14884598
Anthranilic acid methyl ester	8635	-4.5022	-5.9	-5.2011	0.98839386
Mannitol	6251	-5.4638	-4.9	-5.1819	0.3986668
2,6-Dimethoxy-hydroquinone	96038	-4.8364	-5.5	-5.1682	0.46923606

3-Octanone	246728	-5.2265	-5.1	-5.16325	0.08944901
Levoglucosan	2724705	-4.8212	-5.5	-5.1606	0.47998408
Gallic acid	370	-4.4142	-5.9	-5.1571	1.05061926
n-Octanal	454	-5.206	-5.1	-5.153	0.07495332
L-2-aminoadipic acid	92136	-4.9894	-5.3	-5.1447	0.21962737
p-Hydroxy-acetophenone	7469	-4.4844	-5.8	-5.1422	0.93026968
Anisaldehyde	31244	-4.668	-5.6	-5.134	0.65902352
5,6-Dihydro-4-pentyl- 2,6-dimethyl-4H-1,3,5-dithiazine	528360	-4.8086	-5.4	-5.1043	0.41818295
Gentisic acid	3469	-4.4059	-5.8	-5.10295	0.98577756
Tyrosol	10393	-4.6817	-5.5	-5.09085	0.57862548
Glucosamine	439213	-5.0142	-5.1	-5.0571	0.06066976
3,4-Dihydroxybenzoic acid	72	-4.3518	-5.7	-5.0259	0.95332136
Acetophenone	7410	-4.4256	-5.6	-5.0128	0.8304262
4-deoxypyridoxine	6094	-4.5791	-5.4	-4.98955	0.58046396
Galactal	2734735	-4.6969	-5.2	-4.94845	0.35574542
p-hydroxybenzoic acid	135	-4.2859	-5.6	-4.94295	0.92920902
Benzofuran, 2,3,-dihydro	10329	-4.1153	-5.7	-4.90765	1.12055212
Indole	798	-4.1834	-5.6	-4.8917	1.00168747
1,6-dioxaspiro[4.4]non-3-ene	10374471	-4.3218	-5.4	-4.8609	0.76240253
Resorcinol	5054	-3.7987	-5.9	-4.84935	1.48584348
2-Heptanone	8051	-4.8427	-4.8	-4.82135	0.03019346
Pyrogallol	1057	-4.1368	-5.5	-4.8184	0.96392796
Rhamnose	25310	-4.7176	-4.9	-4.8088	0.12897628
Heptanal	8130	-4.709	-4.9	-4.8045	0.1350574
Levulinic acid	11579	-4.8489	-4.7	-4.77445	0.1052882
Leucine	6106	-4.5398	-5	-4.7699	0.32541054
Histidinol	776	-4.3507	-5.1	-4.72535	0.52983511
3-hydroxy-2,3 dihydromaltol	119838	-4.2345	-5.2	-4.71725	0.6827116
Niacinamide	936	-4.2304	-5.2	-4.7152	0.68561074
Glutaric acid	743	-4.7293	-4.7	-4.71465	0.02071823
p-Hydroxy-benzaldehyde	126	-3.9647	-5.4	-4.68235	1.01491036
p-Hydroxybenzyl alcohol	125	-4.1477	-5.1	-4.62385	0.67337779
Benzaldehyde	240	-3.9888	-5.2	-4.5944	0.85644773
2-deoxy-D-ribose	5460005	-4.6828	-4.4	-4.5414	0.1999698
2-acetylpyrrole	14079	-4.1426	-4.9	-4.5213	0.53556268
Proline	145742	-4.1668	-4.8	-4.4834	0.44774001
Succinic acid	1110	-4.3544	-4.6	-4.4772	0.17366543
Arabitol	94154	-4.7375	-4.2	-4.46875	0.38006989
Hexanal	6184	-4.3103	-4.6	-4.45515	0.20484883
adenine	190	-3.6329	-5.2	-4.41645	1.10810704

1,4-benzoquinone	4650	-3.7956	-4.9	-4.3478	0.78092873
L-threonine	6288	-4.0912	-4.3	-4.1956	0.1476439
Aminobutyric acid	119	-4.361	-4	-4.1805	0.25526555
1, 2, 3-Butanetriol	20497	-4.0756	-4.2	-4.1378	0.08796408
6-azacytosine	70265	-3.6023	-4.6	-4.10115	0.70548044
3,5-Dimethyl-1,2,4-trithiolane	32033	-4.4859	-3.7	-4.09295	0.55571522
Creatinine	588	-3.7662	-4.2	-3.9831	0.30674292
Choline	305	-4.2453	-3.3	-3.77265	0.66842804
Epicatechin	72276	1.6845	-8.2	-3.25775	6.98939698
Formic acid	284	-3.1066	-2.9	-3.0033	0.14608826

Table S5. Phytochemicals docking values against BK2R. Values in kcal/mol.

Phytochemical	PubChem CID	Moe Result	AutoVina Result	Mean value	Deviation
Isorhamnetin rutinoside	133562525	-7.9376	-9.5	-8.7188	1.10478363
Heptadecanoic acid	10465	-6.3952	-10.9	-8.6476	3.18537463
7β-Hydroxy-sitosterol	12309569	-7.49	-9.8	-8.645	1.63341666
$_{\gamma}$-sitosterol	457801	-7.5079	-9.7	-8.60395	1.55004878
7α-Hydroxy sitosterol	161816	-7.0381	-10.1	-8.56905	2.16509025
Amentoflavone	5281600	-7.7697	-9	-8.38485	0.86995347
JSM-10292	25019714	-7.4507	-9.3	-8.37535	1.30765257
Kaempferol-3-rutinoside	5318767	-8.657	-7.9	-8.2785	0.53527983
Bioallethrin	15558638	-6.4943	-9.9	-8.19715	2.40819356
Isorhamnetin 3-O-rutinoside	5481663	-8.8637	-7.5	-8.18185	0.96428152
Quercetin rhamnoside	15939939	-6.6669	-9.1	-7.88345	1.72046151
Solanidine	65727	-6.2638	-9.3	-7.7819	2.14691761
Piperine	638024	-5.646	-9.9	-7.773	3.00803225
Quercetin dihexoside	5320835	-7.94	-7.6	-7.77	0.24041631
Isorhamnetin-3-O-glucoside	5318645	-6.6619	-8.7	-7.68095	1.44115433
Cholecalciferol	5280795	-7.3929	-7.8	-7.59645	0.28786317
Kaempferol-3-O-glucoside	5282102	-6.924	-8.2	-7.562	0.90226825
Epigallocatechin gallate	65064	-6.6323	-8.4	-7.51615	1.24995266
Beta-Sitosterol	222284	-7.1653	-7.6	-7.38265	0.30737932
Dihydrokavain	10220256	-5.8489	-8.9	-7.37445	2.1574535
β-carotene	5280489	-6.9206	-7.8	-7.3603	0.6218297
Dibutyl phthalate	3026	-6.3725	-8.3	-7.33625	1.36294832
9-amino-camptothecin	72402	-5.5655	-9.1	-7.33275	2.49926892
Quercitrin	5280459	-6.6531	-8	-7.32655	0.95240212

Neophytadiene	10446	-7.1141	-7.5	-7.30705	0.27287251
Quercetin-3-glucoside	5280804	-6.387	-8.2	-7.2935	1.28198459
Isolariciresinol	160521	-6.2642	-8.3	-7.2821	1.43952799
Quercetin-3-O-glucoside	25203368	-6.5624	-8	-7.2812	1.01653671
Apoatropine	64695	-6.0488	-8.5	-7.2744	1.73326014
Kaempferol rhamnoside	5835713	-6.3532	-8.1	-7.2266	1.23517413
Dicaffeoylquinic acid	12358846	-7.7104	-6.7	-7.2052	0.71446069
Neoxanthin	5282217	-8.3686	-6	-7.1843	1.67485312
Isotachioside	15098566	-5.5622	-8.8	-7.1811	2.28947034
Epicatechin gallate	107905	-6.9613	-7.4	-7.18065	0.31020774
Aminobutyric acid	119	-3.843	-10.5	-7.1715	4.70720984
Vanillic acid	8468	-4.2275	-10.1	-7.16375	4.15248457
Kavain	5281565	-5.1955	-9.1	-7.14775	2.76089843
6-aminochrysene	17534	-5.1614	-9.1	-7.1307	2.78501077
Phenylalanine	6140	-4.8314	-9.2	-7.0157	3.08906668
Kaempferol pentoside	14749097	-6.4984	-7.5	-6.9992	0.70823815
Isopilosine	72312	-6.2557	-7.7	-6.97785	1.02127432
Isopropyl dodecanoate	25068	-6.5275	-7.4	-6.96375	0.61695067
Succinic acid	1110	-3.6245	-10.3	-6.96225	4.72029132
Alpha-tocotrienol	5282347	-7.6184	-6.3	-6.9592	0.93224958
8-dehydrogingerdione	131752598	-6.7783	-7.1	-6.93915	0.22747625
Carvacrol	10364	-4.8738	-9	-6.9369	2.917664
Choline	305	-4.1386	-9.7	-6.9193	3.93250365
10-gingerdione	5317591	-6.6196	-7.2	-6.9098	0.41040478
(+)-Neo-olivil	9976812	-6.3853	-7.4	-6.89265	0.71750125
Incensole oxide acetate	73755086	-5.884	-7.9	-6.892	1.42552727
Salicylic alcohol	5146	-4.0357	-9.7	-6.86785	4.00526494
Hecogenin	91453	-6.5983	-7.1	-6.84915	0.35475547
1,2-Diguaiacyl-1,3-propanediol	6426042	-6.6885	-7	-6.84425	0.22026376
Myristic acid	11005	-5.8632	-7.8	-6.8316	1.36952441
Sitosterol-β-D-glucoside	91884650	-7.9608	-5.7	-6.8304	1.59862701
Neochlorogenic acid	5280633	-6.2118	-7.4	-6.8059	0.84018428
Niacinamide	936	-3.7997	-9.8	-6.79985	4.24285282
Apigenin-7-O-glucoside	44257792	-6.2744	-7.3	-6.7872	0.72520871
Podocarpic acid	93017	-5.0645	-8.5	-6.78225	2.42926535
4-O-Caffeoylquinic acid	58427569	-6.1609	-7.4	-6.78045	0.87617601
Osthole	10228	-6.0402	-7.5	-6.7701	1.03223448
Phytosterols	12303662	-7.3182	-6.2	-6.7591	0.7906868
Catechin	9064	-6.27	-7.2	-6.735	0.65760931
Peonidin 3-O-rutinoside	44256842	-7.4297	-6	-6.71485	1.01095057
β-Selinene	442393	-5.9182	-7.5	-6.7091	1.11850151
Vitamin B5	6613	-5.2423	-8	-6.62115	1.94998837

Esculetin	5281416	-4.8241	-8.4	-6.61205	2.52854314
3,4-dimethoxychalcone	5354494	-5.4976	-7.7	-6.5988	1.55733197
Eugenol	3314	-4.7865	-8.4	-6.59325	2.55513035
Caffeoyl feruloyl tartaric acid	129724266	-7.3494	-5.8	-6.5747	1.09559125
δ-Cadinene	441005	-5.7434	-7.4	-6.5717	1.17139309
Caffeoyl tartaric acid	9857913	-5.4369	-7.7	-6.56845	1.60025336
Farnesylacetone	1711945	-6.5233	-6.6	-6.56165	0.05423509
Chlorogenic acid	1794427	-5.6932	-7.4	-6.5466	1.20688985
Catechin hydrate	107957	-5.6771	-7.4	-6.53855	1.21827427
Quinic acid	6508	-4.2436	-8.8	-6.5218	3.22186134
[1,1-Bicyclopropyl-2-octanoic acid 2hexyl-methyl ester	50930793	-6.9073	-6.1	-6.50365	0.5708473
Caffeoylquinic acid	10155076	-6.1991	-6.8	-6.49955	0.42490046
β-Bisabolene	10104370	-5.7508	-7.2	-6.4754	1.02473915
α-Copaene	442355	-5.4413	-7.5	-6.47065	1.45572073
γ-Cadinene	92313	-5.6146	-7.3	-6.4573	1.19175777
4-shogaol	9794897	-5.9798	-6.9	-6.4399	0.65067966
Rutin	5280805	-8.5605	-4.3	-6.43025	3.01262844
α-Selinene	10856614	-5.3092	-7.5	-6.4046	1.54912954
Calamenene	6429077	-5.58	-7.2	-6.39	1.14551299
Pukateine	442340	-5.6293	-7.1	-6.36465	1.03994194
9-Hydroxy-10,12-octadecadienoic acid	1927	-6.4157	-6.3	-6.35785	0.08181225
Flavone	10680	-5.013	-7.7	-6.3565	1.89999592
Abscisic acid	5375199	-5.8066	-6.9	-6.3533	0.77315055
β - Vetivenene	14475467	-5.0902	-7.6	-6.3451	1.7746966
Luteolin	5280445	-5.1511	-7.5	-6.32555	1.66092312
6-benzylaminopurine	62389	-5.5296	-7.1	-6.3148	1.11044049
Pyrogallol	1057	-3.9165	-8.7	-6.30825	3.38244529
Ursolic acid	64945	-6.7729	-5.8	-6.28645	0.68794419
Epicatechin	72276	-5.637	-6.9	-6.2685	0.89307586
Homovanillyl alcohol	16928	-4.9337	-7.6	-6.26685	1.88535881
Arbutine	440936	-5.5176	-7	-6.2588	1.04821509
Indole	798	-3.9141	-8.6	-6.25705	3.31343167
4-(4-Hydroxy-2,6,6-trimethyl1-cyclohexen-1-yl)-3-buten2-one	538953	-5.8071	-6.7	-6.25355	0.63137564
β-Bourbonene	62566	-5.379	-7.1	-6.2395	1.21693077
β-Caryophyllene	5281515	-5.3772	-7.1	-6.2386	1.21820356
β - Sesquiphellandrene	519764	-5.6425	-6.8	-6.22125	0.8184761
α-Humulene	5281520	-5.3147	-7.1	-6.20735	1.26239774
Caffeoylmalic acid	6124299	-5.4902	-6.9	-6.1951	0.99687914

1,2-Benzenedicarboxylic acid	90531	-5.578	-6.8	-6.189	0.86408449
Silane, triethyl(2-phenylethoxy)	610043	-5.6576	-6.7	-6.1788	0.73708811
Methyl palmitate	8181	-6.6989	-5.6	-6.14945	0.77703964
cis-10-Heptadecenoic acid	5312435	-6.286	-6	-6.143	0.20223254
α-Longipinene	520957	-5.2074	-7	-6.1037	1.26755962
Violaxanthin	448438	-6.9924	-5.2	-6.0962	1.26741819
Eleutheroside B	5316860	-6.5861	-5.6	-6.09305	0.697278
2H-Indeno[1,2-b]furan-2-one, 3,3a,4,5,6,7,8,8b-octahydro-8,8-dimethyl	605626	-5.162	-7	-6.081	1.29966226
Proline	145742	-3.7556	-8.4	-6.0778	3.28408673
Borneol	64685	-4.0251	-8.1	-6.06255	2.88138942
Harmol	68094	-5.009	-7.1	-6.0545	1.47856028
3-Hydroxy-damascone	5366075	-5.5994	-6.5	-6.0497	0.63682037
4-(3-Hydroxy-1-butyn-1-yl)- 3,5,5-trimethyl-2-cyclohexen-1-ol	5280654	-5.8612	-6.2	-6.0306	0.23956778
Anthranilic acid methyl ester	8635	-4.2414	-7.8	-6.0207	2.51631019
Arabitol	94154	-4.1238	-7.9	-6.0119	2.67017663
Palmitic acid	985	-6.621	-5.4	-6.0105	0.86337738
4-methyl-7-ethoxycoumarin	66595	-5.2972	-6.7	-5.9986	0.99192939
Cumin aldehyde	326	-4.4513	-7.5	-5.97565	2.15575644
Caryophyllene oxide	1742210	-5.5094	-6.4	-5.9547	0.6297493
α- Terpinyl acetate	111037	-5.4041	-6.5	-5.95205	0.77491832
ar-Curcumene	92139	-5.4835	-6.4	-5.94175	0.64806336
Ferulic acid	445858	-4.7655	-7.1	-5.93275	1.65074078
β-Ionone	638014	-5.4608	-6.4	-5.9304	0.66411469
(E)-Geranyl acetone	1713001	-5.7356	-6.1	-5.9178	0.25766971
Dimethyl-L-tartrate	11851	-4.6312	-7.2	-5.9156	1.8164159
Carnosol	442009	-6.1763	-5.6	-5.88815	0.40750564
Linolenic acid	5280934	-6.4517	-5.3	-5.87585	0.81437488
Deoxyharringtonine	285342	-7.0439	-4.7	-5.87195	1.65738758
Myristoleic acid	5281119	-5.8346	-5.9	-5.8673	0.04624478
Estra-1,3,5(10)-trien-17B-ol	9811784	-5.6215	-6.1	-5.86075	0.33835059
Phytol	5280435	-7.086	-4.6	-5.843	1.75786746
Flavan	94156	-5.0825	-6.6	-5.84125	1.07303454
Camphor	2537	-3.8695	-7.8	-5.83475	2.7792832
Incensole oxide	90470329	-6.0561	-5.6	-5.82805	0.3225114
p-Cymene	7463	-4.756	-6.9	-5.828	1.51603694

Resorcinol	5054	-3.447	-8.2	-5.8235	3.36087853
Mannitol	6251	-4.1127	-7.5	-5.80635	2.3951828
Farnesol	445070	-5.9907	-5.6	-5.79535	0.27626662
Lotaustralin	441467	-5.2855	-6.3	-5.79275	0.71735983
Feruloyl malate	71694479	-5.5754	-6	-5.7877	0.30023754
Oxime- methoxy-phenyl	9602988	-4.2618	-7.3	-5.7809	2.14833182
α-Ionone	5282108	-5.3832	-6.1	-5.7416	0.50685414
p-Coumaroylmalic acid	129720114	-5.6708	-5.8	-5.7354	0.0913582
α- Copaene-8-ol	25086830	-5.664	-5.8	-5.732	0.09616652
Dodecendioic acid	5283028	-5.6541	-5.8	-5.72705	0.10316688
Isorhamnetin	5281654	-5.753	-5.7	-5.7265	0.03747666
1-Dodecanamine, N, N-dimethyl	8168	-6.0455	-5.4	-5.72275	0.45643743
Carvylacetate	7335	-5.641	-5.8	-5.7205	0.11242998
Naringenin	932	-5.409	-6	-5.7045	0.41790011
Kaempferol	5280863	-5.3012	-6.1	-5.7006	0.5648369
4-methylmethoxycoumarin	223821	-5.1011	-6.3	-5.70055	0.84775032
Palmitoleic acid	445638	-6.3994	-5	-5.6997	0.98952523
Gentisic acid	3469	-4.2903	-7.1	-5.69515	1.98675792
DL-a aminopimelic acid	101122	-4.4749	-6.9	-5.68745	1.71480466
Convolvamine	420422	-5.7703	-5.6	-5.68515	0.12042028
Integerrimine	5281733	-5.8574	-5.5	-5.6787	0.25271996
Carvone	7439	-4.7445	-6.6	-5.67225	1.31203663
Lutein	5281243	-7.0445	-4.3	-5.67225	1.94065456
3,4-Dimethyl-5-pentylidene-2(5H)-furanone	6433214	-5.3365	-6	-5.66825	0.46916535
Sinapyl alcohol	5280507	-5.4247	-5.9	-5.66235	0.33608785
Carvacryl acetate	80792	-5.2166	-6.1	-5.6583	0.62465813
3-Oxo-a-ionol	5370052	-4.8799	-6.4	-5.63995	1.07487302
L-2-aminoadipic acid	92136	-4.447	-6.8	-5.6235	1.66382226
Phosphatidylcholine	10425706	-5.7195	-5.5	-5.60975	0.15520994
4-aminoantipyrine	2151	-4.7761	-6.4	-5.58805	1.1482707
Isopropyl-β-D-thio-galacto-pyranoside	656894	-5.4478	-5.7	-5.5739	0.17833233
3,4-Dimethyl-5-pentylfuran-2(5H)-one	13192443	-5.3792	-5.7	-5.5396	0.22683986
1-Methyl naphthalene	7002	-4.5469	-6.5	-5.52345	1.38105025
Cinnamyl acetate	5282110	-5.2274	-5.8	-5.5137	0.40488934
Methyl eugenol	7127	-5.4876	-5.5	-5.4938	0.00876812
Decan-2-one	12741	-5.0637	-5.9	-5.48185	0.5913534
Tachioside	11962143	-5.6605	-5.3	-5.48025	0.25491199
Benzaldehyde	240	-3.7431	-7.2	-5.47155	2.44439743

adenine	190	-3.7244	-7.2	-5.4622	2.45762033
Secoisolariciresinol	65373	-6.2135	-4.7	-5.45675	1.07020611
Thymol	6989	-4.5802	-6.3	-5.4401	1.21608224
Hexahydrofarnesylacetone	10408	-6.5722	-4.3	-5.4361	1.60668803
Caffeic acid	689043	-4.6311	-6.2	-5.41555	1.10937983
1,2-O-isopropylidene-D-glucoside	87704	-5.1208	-5.7	-5.4104	0.40955625
Hydroxycinnamaldehyde	71407359	-4.3221	-6.4	-5.36105	1.46929718
Creatinine	588	-3.6165	-7.1	-5.35825	2.46320647
Myricetin	5281672	-5.5059	-5.2	-5.35295	0.21630396
Apigenin	5280443	-5.4571	-5.2	-5.32855	0.18179715
α- Terpineol	17100	-4.909	-5.7	-5.3045	0.55932146
p-coumaric acid	637542	-4.5887	-6	-5.29435	0.9979398
Scopoletin	5280460	-5.0647	-5.5	-5.28235	0.30780358
1-Hydroxy-1-(4-hydroxyphenyl)-2-propanone	10261435	-5.0526	-5.5	-5.2763	0.31635957
Geranyl acetone	1549778	-5.4337	-5.1	-5.26685	0.23596153
O-Feruloyl quinic acid	10177048	-5.8298	-4.7	-5.2649	0.79888924
Carnosine	439224	-4.9741	-5.5	-5.23705	0.37186746
n-acetyl-L-glutamine	182230	-4.4594	-6	-5.2297	1.08936871
β-Homocyclocitral	61124	-4.8342	-5.6	-5.2171	0.54150237
Arachidonic acid	444899	-6.7279	-3.7	-5.21395	2.14104862
Formic acid	284	-2.609	-7.8	-5.2045	3.6705913
Hydrocotarnine	3646	-5.3011	-5.1	-5.20055	0.14219917
Quercetin	5280343	-5.7987	-4.6	-5.19935	0.8476089
m-Hydroxy-acetophenone	8487	-4.0782	-6.3	-5.1891	1.57104985
2(3H)-Naphthalenone, 4, 4a,5,6,7,8- hexahydro-1-methoxy	534313	-4.7437	-5.6	-5.17185	0.60549554
Genistein	5280961	-5.2302	-5.1	-5.1651	0.0920653
Benzyl salicilate	8363	-5.2847	-5	-5.14235	0.2013133
4-Acetyl-2-methylphenol	70135	-4.5647	-5.7	-5.13235	0.80277833
Tyrosol	10393	-4.3361	-5.9	-5.11805	1.1058443
Syringic acid	10742	-5.0142	-5.2	-5.1071	0.13138044
(E)-Anethole	637563	-4.5952	-5.6	-5.0976	0.71050089
Methylcoumarin	17130	-4.4802	-5.7	-5.0901	0.86252885
Cinnamide	5273472	-4.5747	-5.6	-5.08735	0.72499658
Bornyl acetate	6448	-4.9742	-5.2	-5.0871	0.15966471
Hexanal	6184	-4.1511	-6	-5.07555	1.30736973
DL-methyl-m-tyrosine	2110	-5.0145	-5.1	-5.05725	0.06045763
Decanal	8175	-5.0991	-5	-5.04955	0.07007428
Glucosamine	439213	-4.1962	-5.9	-5.0481	1.20476853
Citric acid	311	-4.2927	-5.8	-5.04635	1.06582205

Lauric acid	3893	-5.4902	-4.6	-5.0451	0.62946646
β-Cyclocitral	9895	-4.4352	-5.6	-5.0176	0.82363798
2-Pentylfuran	19602	-5.0254	-5	-5.0127	0.01796051
2-(1-Pentenyl)furan	5369956	-4.9239	-5.1	-5.01195	0.1245215
Sinapic acid	637775	-5.1094	-4.9	-5.0047	0.14806816
Shikimic acid	8742	-3.9934	-6	-4.9967	1.41888047
Leucine	6106	-4.3489	-5.6	-4.97445	0.88466129
Pterostilbene	5281727	-5.6394	-4.3	-4.9697	0.94709882
p-Hydroxy-acetophenone	7469	-4.2674	-5.6	-4.9337	0.9422905
4-Vinyl guaiacol	332	-4.5618	-5.3	-4.9309	0.52198623
Anthocyanins	145858	-5.1165	-4.7	-4.90825	0.29450997
2,4,6-Trimethyl-5H-1,3,5-dithiazine	12518	-4.2078	-5.6	-4.9039	0.98443406
Synephrine	7172	-5.3322	-4.4	-4.8661	0.65916494
Nonanol	8914	-5.2117	-4.5	-4.85585	0.5032479
Homatropine	5282593	-5.4974	-4.2	-4.8487	0.91740034
2, 6,-Nonadienal, 3, 7-dimethyl	5364526	-5.3957	-4.3	-4.84785	0.7747769
Umbelliferone	5281426	-4.6106	-5	-4.8053	0.27534738
5,6-Dihydro-4-pentyl- 2,6-dimethyl-4H-1,3,5-dithiazine	528360	-4.7002	-4.9	-4.8001	0.14127993
Coniferol	1549095	-5.0621	-4.5	-4.78105	0.39746472
Crysoeriol	5280666	-5.4412	-4.1	-4.7706	0.94837161
Heptanal	8130	-4.1084	-5.4	-4.7542	0.91329912
2,6-Dimethoxy-hydroquinone	96038	-4.4926	-5	-4.7463	0.35878598
4-deoxypyridoxine	6094	-4.5608	-4.9	-4.7304	0.23985062
L-threonine	6288	-3.8652	-5.5	-4.6826	1.15597817
Diphenyl	7095	-4.7619	-4.6	-4.68095	0.11448059
Adenosine	60961	-4.729	-4.6	-4.6645	0.09121677
Acetophenone	7410	-4.0987	-5.2	-4.64935	0.7787367
Indole-3-carboxaldehyde	10256	-4.2786	-5	-4.6393	0.51010683
Harmine	5280953	-5.0662	-4.2	-4.6331	0.61249589
Rhamnose	25310	-4.1071	-5.1	-4.60355	0.70208632
Phthalic acid	1017	-4.1274	-5	-4.5637	0.61702138
3-Octanone	246728	-4.5131	-4.6	-4.55655	0.06144758
p-Hydroxybenzyl alcohol	125	-4.0127	-5.1	-4.55635	0.7688372
Nonanal	31289	-4.608	-4.5	-4.554	0.07636753
Linalool	6549	-5.0963	-4	-4.54815	0.77520116
n-Octanal	454	-4.4863	-4.6	-4.54315	0.08039804
Safranal	61041	-4.3466	-4.7	-4.5233	0.24989154
3,4-Dihydroxybenzoic acid	72	-3.9448	-5.1	-4.5224	0.81684975
Naphthalene	931	-4.1375	-4.9	-4.51875	0.53916892

p-Hydroxy-benzaldehyde	126	-3.7679	-5.2	-4.48395	1.01264762
Methyl chavicol	8815	-4.7947	-4.1	-4.44735	0.49122708
1,6-dioxaspiro[4.4]non-3-ene	10374471	-4.0949	-4.7	-4.39745	0.42787031
3-hydroxy-2,3 dihydromaltol	119838	-4.1745	-4.5	-4.33725	0.23016326
Levulinic acid	11579	-3.8395	-4.8	-4.31975	0.67917606
Gallic acid	370	-4.1275	-4.4	-4.26375	0.1926866
2-Heptanone	8051	-4.1249	-4.4	-4.26245	0.19452508
p-hydroxybenzoic acid	135	-3.7817	-4.7	-4.24085	0.64933616
Benzofuran, 2,3,-dihydro	10329	-3.8269	-4.6	-4.21345	0.54666425
Glutaric acid	743	-3.7356	-4.5	-4.1178	0.54051242
Levoglucosan	2724705	-3.8328	-4.4	-4.1164	0.40107097
2-acetylpyrrole	14079	-3.8702	-4.3	-4.0851	0.30391449
Histidinol	776	-4.1646	-3.9	-4.0323	0.18710045
1,4-benzoquinone	4650	-3.5998	-4.4	-3.9999	0.56582685
6-azacytosine	70265	-3.3645	-4.6	-3.98225	0.87363043
Anisaldehyde	31244	-3.9278	-3.9	-3.9139	0.01965757
1, 2, 3-Butanetriol	20497	-3.8812	-3.8	-3.8406	0.05741707
2-deoxy-D-ribose	5460005	-3.7603	-3.9	-3.83015	0.09878282
Cinnamic acid	444539	-4.4157	-3	-3.70785	1.00105107
3,5-Dimethyl-1,2,4-trithiolane	32033	-3.8905	-3.3	-3.59525	0.41754655
Galactal	2734735	-4.0501	-2.9	-3.47505	0.81324351

Table S6. The affinity values and residues of HR1 that interacted with fexofenadine and the best 15 molecules, with both software.

Phytochemical	Mean Value of Affinity	Software	Affinity Value	Residues	Number of residues forming interactions	Number of repeated residues
7α-Hydroxy sitosterol	-7.465	MOE	-6.230	**Phe116**, **Leu154**, **Leu157**, **Trp158**, Ile160, His167, Phe168, Trp189, **Phe190**, **Thr194**, **Ile197**, Asn198	12	7
		AutoVina	-8.7	**Phe116**, Phe119, Ala151, **Leu154**, **Leu157**, **Trp158**, Pro161, **Phe190**, **Thr194**, **Ile197**, Leu201, Pro202	12	
β-Sitosterol	-7.387	MOE	-6.075	**Leu154**, **Leu157**, **Trp158**, Ile160, **Pro161**, His167, Phe168, Trp189, **Phe190**, Thr194, **Ile197**	10	5
		AutoVina	-8.7	Phe116, Phe119, Ala151, **Leu154**, **Leu157**, **Trp158**, **Pro161**, **Phe190**, **Ile197**, Leu201, Pro202	10	
γ-sitosterol	-7.337	MOE	-6.075	**Phe116**, **Leu154**, **Leu157**, **Trp158**, Ile160, **Pro161**, His167, Phe168, Trp189, **Phe190**, Met193, **Ile197**	12	7

		AutoVina	-8.6	**Phe116**, Phe119, **Leu154**, **Leu157**, **Trp158**, **Pro161**, **Phe190**, **Ile197**, Leu201, Pro202	10	
Phytosterols	-7.331	MOE	-5.962	**Leu154**, **Leu157**, **Trp158**, Ile160, **Pro161**, His 167, Trp189, **Phe190**, Met193, Thr194, **Ile197**	11	6
		AutoVina	-8.7	Phe116, Phe119, **Leu154**, **Leu157**, **Trp158**, **Pro161**, **Phe190**, **Ile197**, Leu201, Pro202	10	
Fexofenadine	-7.311	MOE	-6.522	**Leu154**, **Leu157**, **Trp158**, **Ile160**, **Pro161**, His167, Trp189, **Phe190**, Met193, Thr194, **Ile197**, Asn198	12	7
		AutoVina	-8.1	Phe116, Phe119, **Leu154**, **Leu157**, **Trp158**, **Ile160**, **Pro161**, **Phe190**, **Ile197**, Pro202	10	
Alpha-tocotrienol	-7.310	MOE	-6.821	**Phe116**, **Leu154**, **Leu157**, **Trp158**, Ile160, **Pro161**, His167, Phe168, Trp189, **Phe190**, **Met193**, **Thr194**, **Ile197**, Asn198	14	9
		AutoVina	-7.8	**Phe116**, Phe119, Ala151, **Leu154**, **Ile157**, **Trp158**, **Pro161**, **Phe190**, **Met193**, **Thr194**, **Ile197**, Pro202	12	
Cholecalciferol	-7.294	MOE	-6.088	**Phe116**, **Leu154**, **Leu157**, **Trp158**, Ile160, **Pro161**, His167, **Phe190**, **Met193**, **Thr194**, **Ile197**	11	9
		AutoVina	-8.5	**Phe116**, Ile120, Gly150, Ala151, **Leu154**, **Leu157**, **Trp158**, **Pro161**, **Phe190**, **Met193**, **Thr194**, **Ile197**, Asn198,	13	
Solanidine	-7.234	MOE	-5.369	**Phe116**, **Leu154**, **Leu157**, **Trp158**, Ile160, **Pro161**, His167, **Phe190**, **Met193**, **Ile197**	10	8
		AutoVina	-9.1	**Phe116**, **Leu154**, **Leu157**, **Trp158**, **Pro161**, **Phe190**, **Met193**, Thr194, **Ile197**	9	
Hecogenin	-7.091	MOE	-5.183	**Phe116**, **Leu154**, **Leu157**, **Trp158**, **Ile160**, **Pro161**, His167, **Phe190**, **Ile197**	9	8
		AutoVina	-9	**Phe116**, **Leu154**, **Leu157**, **Trp158**, **Ile160**, **Pro161**, **Phe190**, **Ile197**, Asn198, Leu201	10	
Apigenin-7-O-glucoside	-6.892	MOE	-5.884	Thr112, **Phe116**, **Leu154**, **Leu157**, **Trp158**, **Ile160**, **Pro161**, Phe168, **Phe190**, Met193, **Thr194**, **Ile197**, Asn198	13	9
		AutoVina	-7.9	**Phe116**, Phe119, Ile120, Ala151, **Leu154**, Ser155, **Leu157**, **Trp158**, **Ile160**, **Pro161**, **Phe190**, **Thr194**, **Ile197**	13	
Neoxanthin	-6.856	MOE	-7.112	**Phe116**, **Leu154**, **Leu157**, **Trp158**, Ile160, **Pro161**, His167, Phe168, Met169, Gln170, Val187, Trp189, **Phe190**, **Met193**, **Ile197**	15	8
		AutoVina	-6.6	**Phe116**, Phe119, **Leu154**, **Leu157**, **Trp158**, **Pro161**, **Phe190**, **Met193**, thr194, **Ile197**, Pro202	11	
Piperine	-6.704	MOE	-5.409	Thr112, **Phe116**, **Leu154**, **Leu157**, **Trp158**, Ile160, **Pro161**, His167, Trp189, **Phe190**, **Ile197**, Asn198	12	7
		AutoVina	-8	**Phe116**, Phe119, Ile120, Ala151, **Leu154**, **Leu157**, **Trp158**, **Pro161**, **Phe190**, Thr194, **Ile197**	11	
8-dehydrogingerdione	-6.698	MOE	-6.296	**Thr112**, **Phe116**, **Leu154**, Ser155, **Leu157**, **Trp158**, Ile160, **Pro161**, His167, Phe168, Trp189, **Phe190**, **Met193**, **Thr194**, **Ile197**	15	10
		AutoVina	-7.1	**Thr112**, **Phe116**, Phe119, Ala151, **Leu154**, **Leu157**, **Trp158**, **Pro161**,	13	

				**Phe190**, **Met193**, **Thr194**, **Ile197**, Asn198		
6-aminochrysene	-6.646	MOE	-4.493	**Phe116**, **Leu154**, **Leu157**, **Trp158**, **Pro161**, **Phe190**, Met193, **Ile197**	8	7
		AutoVina	-8.8	**Phe116**, **Leu154**, **Leu157**, **Trp158**, **Pro161**, **Phe190**, Thr194, **Ile197**, Asn198	9	
Chlorogenic acid	-6.633	MOE	-5.966	Thr112, **Phe116**, **Leu154**, Ser155, **Leu157**, **Trp158**, Ile160, **Pro161**, His167, Phe168, Trp189, **Phe190**, **Thr194**, **Ile197**, **Asn198**	15	9
		AutoVina	-7.3	**Phe116**, Phe119, **Leu154**, **Leu157**, **Trp158**, **Pro161**, **Phe190**, Met193, **Thr194**, **Ile197**, **Asn198**, Leu201, Pro202	13	
3,4-dimethoxychalcone	-6.611	MOE	-5.322	**Phe116**, **Leu154**, **Leu157**, **Trp158**, Pro161, His167, Phe168, Trp189, Phe190, Met193, **Thr194**, **Ile197**, **Asn198**	13	7
		AutoVina	-7.9	**Phe116**, Phe119, **Leu154**, **Leu157**, **Trp158**, **Thr194**, **Ile197**, **Asn198**, Pro202	9	

The residues in bold are the residues that form interactions with the molecule in both software.

Table S7. The affinity values and residues of NKR1 that interacted with Aprepitant and the best 15 molecules, with both software.

Phytochemical	Mean Value of Affinity	Software	Affinity Value	Residues	Number of residues forming interactions	Number of repeated residues
Amentoflavone	-9.604	MOE	-8.209	**Asn89**, **Tyr92**, **Ala93**, His108, **Asn109**, **Pro112**, **Ile113**, Val116, Phe164, **Gln165**, Cys180, **Ile182**, **His197**, Thr201, Ile204, His265, **Phe267**, **Phe268**, Pro271, **Tyr287**	20	12
		AutoVina	-11	**Asn89**, **Tyr92**, **Ala93**, Asn96, **Asn109**, **Pro112**, **Ile113**, **Gln165**, **Ile182**, Glu193, **His197**, **Phe267**, **Phe268**, Tyr272, **Tyr287**	15	
Aprepitant	-9.039	MOE	-7.678	**Asn89**, **His108**, **Asn109**, **Pro112**, **Ile113**, Val116, **Gln165**, **Ile182**, Trp184, Glu193, Tyr196, **His197**, **Ile204**, **Trp261**, **Phe264**, **Phe268**, Tyr272, Tyr287	18	12
		AutoVina	-10.4	Met81, Val88, **Asn89**, Tyr92, Ala93, **His108**, **Asn109**, **Pro112**, **Ile113**, **Gln165**, Cys180, **Ile182**, **His197**, Val200, Thr201, **Ile204**, **Trp261**, **Phe264**, His265, **Phe268**, Tyr287, Met291, Met295	23	
Peonidin 3-O-rutinoside	-8.576	MOE	-7.853	**Asn89**, **Tyr92**, **Asn96**, **His108**, **Asn109**, **Pro112**, **Ile113**, **Gln165**, **Ile182**, **His197**, Val200, **Ile204**, **Phe264**, Phe267, **Phe268**, **Tyr272**, Ile283, **Tyr287**	18	15

		AutoVina	-9.3	**Asn89**, **Tyr92**, Ala93, **Asn96**, **His108**, **Asn109**, **Pro112**, **Ile113**, **Gln165**, Cys180, **Ile182**, Glu193, **His197**, **Ile204**, **Phe264**, **Phe268**, Pro271, **Tyr272**, **Tyr287**	19	
Isorhamnetin 3-O-rutinoside	-8.519	MOE	-8.239	**Asn89**, **Tyr92**, Asn96, **His108**, **Asn109**, **Pro112**, **Ile113**, **Gln165**, Phe167, Cys180, **Met181**, **Ile182**, **His197**, Val200, Thr201, **Phe264**, **Phe268**, Ile283, Gln284, **Tyr287**	20	13
		AutoVina	-8.8	**Asn89**, **Tyr92**, Ala93, **His108**, **Asn109**, **Pro112**, **Ile113**, **Gln165**, **Met181**, **Ile182**, Glu193, **His197**, Ile204, **Phe264**, **Phe268**, Pro271, Tyr272, **Tyr287**, Met291	19	
Quercitrin	-8.455	MOE	-7.51	**Asn85**, **Asn89**, **His108**, **Asn109**, **Pro112**, **Ile113**, **Gln165**, **Ile182**, Glu193, **His197**, **Val200**, **Thr201**, Ile204, **Phe264**, **His265**, **Phe268**, **Tyr272**	17	15
		AutoVina	-9.4	**Asn85**, **Asn89**, **His108**, **Asn109**, **Pro112**, **Ile113**, **Gln165**, **Ile182**, **His197**, **Val200**, **Thr201**, **Phe264**, **His265**, **Phe268**, **Tyr272**	15	
Quercetin dihexoside	-8.420	MOE	-7.841	**Asn89**, **Tyr92**, **Ala93**, Asn96, **His108**, **Asn109**, **Pro112**, **Ile113**, **Gln165**, **Ile182**, **Glu193**, **His197**, Trp261, **Phe264**, Phe267, **Phe268**, Ile283, Gln284, **Tyr287**, **Met295**	20	15
		AutoVina	-9	**Asn89**, **Tyr92**, **Ala93**, **His108**, **Asn109**, **Pro112**, **Ile113**, **Gln165**, **Ile182**, **Glu193**, Tyr196, **His197**, **Phe264**, **Phe268**, Pro271, Tyr272, **Tyr287**, Met291, **Met295**	19	
Kaempferol-3-rutinoside	-8.404	MOE	-7.708	**Asn89**, **Tyr92**, **Asn109**, **Pro112**, **Ile113**, **Gln165**, **Met181**, **Ile182**, **Glu193**, **His197**, Thr201, Trp261, **Phe264**, **Phe268**, **Tyr272**, **Tyr287**, **Met291**, Ala294, Met295,	19	15
		AutoVina	-9.1	**Asn89**, **Tyr92**, His108, **Asn109**, **Pro112**, **Ile113**, **Gln165**, Cys180, **Met181**, **Ile182**, **Glu193**, **His197**, Val200, Ile204, **Phe264**, His265, **Phe268**, **Tyr272**, **Tyr287**, **Met291**	20	
Alpha-tocotrienol	-8.402	MOE	-7.804	**Tyr92**, **Ala93**, **Asn96**, **His108**, **Asn109**, **Pro112**, **Ile113**, **Gln165**, **Cys180**, **Ile182**, **His197**, **Val200**, **Ile204**, **Phe264**, **His265**, Phe267, **Phe268**, Ile283, Gln284, **Tyr287**	20	17
		AutoVina	-9	Asn89, **Tyr92**, **Ala93**, **Asn96**, **His108**, **Asn109**, **Pro112**, **Ile113**, **Gln165**, **Cys180**, Met181, **Ile182**, **His197**, **Val200**, **Ile204**, **Phe264**, **His265**, **Phe268**, **Tyr287**	19	
Isorhamnetin-3-O-glucoside	-8.366	MOE	-7.333	Tyr92, His108, **Asn109**, **Pro112**, **Ile113**, **Gln165**, **Ile182**, **Glu193**, Tyr196, **His197**, **Thr201**, **Ile204**, **Phe264**, **Phe268**, **Tyr272**, **Tyr287**	16	13
		AutoVina	-9.4	Asn89, **Asn109**, **Pro112**, **Ile113**, **Gln165**, **Ile182**, Trp184, **Glu193**, **His197**, Val200, **Thr201**, **Ile204**, **Phe264**, His265, **Phe268**, **Tyr272**, **Tyr287**, Met291	18	
Quercetin-3-O-glucoside	-8.176	MOE	-7.353	**Asn89**, Tyr92, **His108**, **Asn109**, **Pro112**, **Ile113**, Val116, **Gln165**, **Ile182**, **Trp184**, **Glu193**, **His197**, **Val200**, **Ile204**, **Phe264**, **Phe268**, Tyr272, Tyr287	18	14

		AutoVina	-9	Asn85, **Asn89**, **His108**, **Asn109**, **Pro112**, **Ile113**, **Gln165**, **Ile182**, **Trp184**, **Glu193**, Tyr196, **His197**, **Val200**, Thr201, **Ile204**, **Phe264**, His265, **Phe268**	18	
Quercetin-3-glucoside	-8.140	MOE	-7.580	**Asn89**, **His108**, **Asn109**, **Pro112**, **Ile113**, **His165**, **Ile182**, **Glu193**, **His197**, **Val200**, **Thr201**, **Ile204**, **Phe264**, **Phe268**, Pro271, Tyr272	16	14
		AutoVina	-8.7	Asn85, **Asn89**, **His108**, **Asn109**, **Pro112**, **Ile113**, **Gln165**, **Ile182**, Trp184, **Glu193**, Tyr196, **His197**, **Val200**, **Thr201**, **Ile204**, **Phe264**, His265, **Phe268**,	18	
Dicaffeoylquinic acid	-8.128	MOE	-7.557	**Asn89**, **Tyr92**, Asn96, **His108**, **Asn109**, **Ile113**, **Gln165**, Cys180, Met181, **His197**, **Val200**, **Thr201**, **Ile204**, **His265**, **Phe264**, **Phe268**, **Tyr287**	17	14
		AutoVina	-8.7	Asn85, **Asn89**, **Tyr92**, **His108**, **Asn109**, Pro112, **Ile113**, **Gln165**, Ile182, **His197**, **Val200**, **Thr201**, **Ile204**, **Phe264**, **His265**, **Phe268**, Tyr272, **Tyr287**	18	
Neoxanthin	-7.964	MOE	-7.129	Phe25, Gln27, **Tyr92**, Asn96, Asn109, Gln165, Ile182, Phe264, **Phe268**, Leu279, Lys281, Phe282, **Ile283**, **Gln284**, **Tyr287**	15	5
		AutoVina	-8.8	Asn89, **Tyr92**, Ala93, His108, Asn189, Lys190, Glu193, Lys194, Phe267, **Phe268**, Pro271, Tyr272, Tyr278, **Ile283**, **Gln284**, **Tyr287**	16	
7α-Hydroxy sitosterol	-7.963	MOE	-6.627	**Asn89**, **Tyr92**, **Ala93**, **Asn96**, **His108**, **Asn109**, **Pro112**, **Ile113**, **Gln165**, His197, **Val200**, **Ile204**, **Phe264**, **His265**, **Phe268**, **Tyr287**, **Met291**	17	16
		AutoVina	-9.3	**Asn89**, **Tyr92**, **Ala93**, **Asn96**, **His108**, **Asn109**, **Pro112**, **Ile113**, Val116, **Gln165**, **Val200**, **Ile204**, Trp261, **Phe264**, **His265**, **Phe268**, Gln284, **Tyr287**, **Met291**	19	
Hecogenin	-7.953	MOE	-6.307	**Asn89**, **Tyr92**, Ala93, **Asn96**, His108, **Asn109**, Pro112, **Gln165**, Arg177, Val179, **Cys180**, Met181, **Phe264**, **Phe268**, **Tyr287**	15	9
		AutoVina	-9.6	**Asn89**, **Tyr92**, **Asn96**, **Asn109**, Ile113, **Gln165**, **Cys180**, Ile182, His197, Val200, Thr201, Ile204, **Phe264**, His265, **Phe268**, **Tyr287**	16	
Sitosterol-β-D-glucoside	-7.953	MOE	-7.106	**Asn89**, **Tyr92**, **Ala93**, **His108**, **Asn109**, **Gln165**, **Cys180**, Met181, **Ile182**, **Glu193**, **His197**, **Phe268**, Pro271, Tyr272, **Tyr287**, Met291	16	12
		AutoVina	-8.8	**Asn89**, **Tyr92**, **Ala93**, Asn96, **His108**, **Asn109**, Ile113, **Gln165**, **Cys180**, **Ile182**, **Glu193**, **His197**, Phe267, **Phe268**, Tyr278, Ile283, **Tyr287**	17	

The residues in bold are the residues that form interactions with the molecule in both software.

Table S8. The affinity values and residues of CLR1 that interacted with Zafirlukast and the best 15 molecules, with both software.

Phytochemical	Mean Value of Affinity	Software	Affinity Value	Residues	Number of residues forming interactions	Number of repeated residues
Zafirlukast	-11.292	MOE	-9.285	Thr100, **Tyr104**, **Tyr108**, **Phe112**, **Thr154**, **Ser155**, **Pro157**, **Phe158**, Ala161, Gln164, Phe174, Glu175, **Pro176**, **Pro177**, **Val186**, **Leu189**, **His190**, **Ser193**, **Val196**, **Gly197**, **Tyr249**, **Arg253**, **Leu257**	23	18
		AutoVina	-13.3	**Tyr104**, **Tyr108**, **Phe112**, Phe150, **Thr154**, **Ser155**, **Pro157**, **Phe158**, **Pro176**, **Pro177**, **Val186**, **Leu189**, **His190**, **Ser193**, **Val196**, **Gly197**, **Tyr249**, Gln252, **Arg253**, His256, **Leu257**, Val277, Thr280, Leu281	24	
Amentoflavone	-9.565	MOE	-7.730	Thr100, **Tyr104**, **Phe158**, Ala161, **Gln164**, **Phe174**, **Glu175**, **Pro176**, **Pro177**, Val186, **Leu189**, His190, **Ser193**, **Arg253**, His256, **Leu257**, **Val277**	17	12
		AutoVina	-11.4	**Tyr104**, Tyr108, Thr154, **Phe158**, **Gln164**, **Phe174**, **Glu175**, **Pro176**, **Pro177**, **Leu189**, **Ser193**, Tyr249, Gln252, **Arg253**, **Leu257**, **Val277**, Thr280, Leu281	18	
Lutein	-9.161	MOE	-8.822	Tyr104, Tyr108, **Phe150**, **Thr154**, Ser155, **Phe158**, Phe174, **Glu175**, **Pro176**, **Pro177**, **Val186**, **Leu189**, His190, **Val192**, **Ser193**, **Val196**, Tyr249, Arg253, Leu257, Val277, Leu281	21	11
		AutoVina	-9.5	**Phe150**, **Thr154**, Pro157, **Phe158**, Gln164, Asp166, Lys165, Lys172, **Glu175**, **Pro176**, **Pro177**, Gln181, **Val186**, **Leu189**, **Val192**, **Ser193**, **Val196**	17	
Alpha-tocotrienol	-9.108	MOE	-8.816	Thr100, **Tyr104**, **Tyr108**, Phe112, **Phe150**, Val151, **Thr154**, **Ser155**, **Phe158**, Phe174, Glu175, Pro176, Pro177, **Val186**, **Ser193**, Val196, Gly197, **Tyr249**, **Arg253**, **Val277**, Leu281	21	11
		AutoVina	-9.4	**Tyr104**, **Tyr108**, **Phe150**, **Thr154**, **Ser155**, **Phe158**, **Val186**, Leu189, His190, Val192, **Ser193**, **Tyr249**, Gln252, **Arg253**, His256, Leu257, **Val277**	17	
Violaxanthin	-8.945	MOE	-8.391	**Tyr108**, **Phe150**, **Thr154**, **Ser155**, **Pro157**, **Phe158**, Ala161, **Gln164**, **Lys165**, **Asp166**, **Lys172**, **Glu175**, **Pro176**, **Gln178**, **Gln181**, **Val186**, **Leu189**, **Val192**, **Ser193**, **Val196**, **Leu257**	21	20
		AutoVina	-9.5	**Tyr108**, **Phe150**, **Thr154**, **Ser155**, **Pro157**, **Phe158**, **Gln164**, **Lys165**, **Asp166**, **Lys172**, **Glu175**, **Pro176**, Pro177, **Gln178**, **Gln181**, **Val186**, **Leu189**, **Val192**, **Ser193**, **Val196**, Gly197, **Leu257**	22	
Epicatechin gallate	-8.938	MOE	-7.677	**Thr100**, **Tyr104**, Tyr108, **Phe158**, **Gln164**, **Phe174**, **Glu175**, **Pro176**, **Pro177**, **Val186**, Leu189, **His190**,	20	17

				**Ser193**, **Tyr249**, **Arg253**, His256, **Leu257**, **Val277**, **Thr280**, **Leu281**		
		AutoVina	-10.2	Arg79, **Thr100**, **Tyr104**, **Phe158**, **Gln164**, **Phe174**, **Glus175**, **Pro176**, **Pro177**, **Val186**, **His190**, **Ser193**, **Tyr249**, Gln252, **Arg253**, **Leu257**, **Val277**, **Thr280**, **Leu281**	19	
Epigallocatechin gallate	-8.923	MOE	-7.846	**Tyr104**, Tyr108, **Phe158**, **Gln164**, **Phe174**, **Glu175**, **Pro176**, **Pro177**, **Val186**, **His190**, **Ser193**, **Tyr249**, **Gln252**, **Arg253**, **His256**, **Leu257**, **Val277**, **Thr280**, **Leu281**	19	18
		AutoVina	-10	Arg79, Thr100, **Tyr104**, **Phe158**, **Gln164**, **Phe174**, **Glu175**, **Pro176**, **Pro177**, **Val186**, **His190**, **Ser193**, **Tyr249**, **Gln252**, **Arg253**, **His256**, **Leu257**, **Val277**, **Thr280**, **Leu281**	20	
β-carotene	-8.874	MOE	-8.249	**Thr154**, Ser155, Pro157, **Phe158**, **Gln164**, **Lys165**, **Asp166**, **Lys172**, **Glu175**, **Pro176**, **Pro177**, **Gln178**, **Gln181**, **Val186**, **Leu189**, **Val192**, **Ser193**, **Val196**, Arg253	19	16
		AutoVina	-9.5	Tyr108, **Thr154**, **Phe158**, **Gln164**, **Lys165**, **Asp166**, **Lys172**, **Glu175**, **Pro176**, **Pro177**, **Gln178**, **Gln181**, **Val186**, **Leu189**, His190, **Val192**, **Ser193**, **Val196**, Leu257	19	
Neoxanthin	-8.872	MOE	-7.845	**Tyr104**, **Phe150**, **Thr154**, **Pro157**, **Phe158**, **Pro177**, **Val186**, **Leu189**, **His190**, **Val192**, **Ser193**, **Val196**, Ile200, Tyr249, **Arg253**, **His256**, **Leu257**	17	15
		AutoVina	-9.9	**Tyr104**, **Phe150**, **Thr154**, **Pro157**, **Phe158**, Pro176, **Pro177**, **Val186**, **Leu189**, **His190**, **Val192**, **Ser193**, **Val196**, Gln252, **Arg253**, **His256**, **Leu257**, Val277, Thr280, Leu281	20	
Sitosterol-β-D-glucoside	-8.746	MOE	-7.992	**Thr100**, **Tyr104**, **Thr154**, **Pro157**, **Phe158**, **Ala161**, **Gln164**, **Glu175**, Pro176, Pro177, **Val186**, **Leu189**, **His190**, **Ser193**, **Arg253**, Leu257, Val277, Leu281	18	13
		AutoVina	-9.5	**Thr100**, **Tyr104**, Tyr108, Phe112, Phe150, Val151, **Thr154**, Ser155, **Pro157**, **Phe158**, **Ala161**, **Gln164**, Phe174, **Glu175**, **Val186**, **Leu189**, **His190**, **Ser193**, Val196, Gly197, **Arg253**	21	
Peonidin 3-O-rutinoside	-8.479	MOE	-7.658	**Thr100**, **Tyr104**, Pro157, **Phe158**, Met160, **Ala161**, **Gln164**, **Phe174**, **Glu175**, **Pro176**, **Val186**, **Leu189**, **His190**, **Tyr249**, **Arg253**, **Leu257**, **Val277**, **Leu281**	18	16
		AutoVina	-9.3	Arg79, **Thr100**, **Tyr104**, **Phe158**, **Ala161**, **Gln164**, **Phe174**, **Glu175**, **Pro176**, Pro177, **Val186**, **Leu189**, **His190**, Ser193, **Tyr249**, **Arg253**, His256, **Leu257**, **Val277**, **Leu281**	20	
Kaempferol-3-rutinoside	-8.418	MOE	-7.536	**Thr100**, **Tyr104**, **Thr154**, **Pro157**, **Phe158**, **Ala161**, Lys162, Phe174, **Glu175**, Pro176, Pro177, **Val186**, **Leu189**, **His190**, **Ser193**, **Tyr249**, **Arg253**, **Leu257**, Val277, Leu281	20	14
		AutoVina	-9.3	**Thr100**, **Tyr104**, Tyr108, **Thr154**, Ser155, **Pro157**, **Phe158**, **Ala161**, Gln164, **Glu175**, **Val186**, Leu189, **His190**, **Ser193**, **Tyr249**, **Arg253**, **Leu257**	17	

Isorhamnetin 3-O-rutinoside	-8.373	MOE	-7.847	**Thr100**, **Tyr104**, **Pro157**, **Phe158**, **Ala161**, **Gln164**, Phe174, **Glu175**, **Pro176**, **Pro177**, **Val186**, **Leu189**, **His190**, **Tyr249**, **Arg253**, His256, Val277, Leu281	18	14
		AutoVina	-8.9	**Thr100**, **Tyr104**, Tyr108, Thr154, Ser155, **Pro157**, **Phe158**, **Ala161**, **Gln164**, **Glu175**, **Pro176**, **Pro177**, **Val186**, **Leu189**, **His190**, Ser193, **Tyr249**, **Arg253**, Leu257	19	
9-amino-camptothecin	-8.291	MOE	-6.582	**Tyr104**, **Tyr108**, **Thr154**, **Ser155**, **Pro157**, **Phe158**, Pro177, **Val186**, **Leu189**, **Ser193**, **Tyr249**, **Arg253**, **Leu257**	13	12
		AutoVina	-10	**Tyr104**, **Tyr108**, **Thr154**, **Ser155**, **Pro157**, **Phe158**, **Val186**, **Leu189**, Val192, **Ser193**, **Tyr249**, **Arg253**, **Leu257**	13	
Quercetin rhamnoside	-8.282	MOE	-7.364	**Tyr104**, **Tyr108**, **Phe112**, **Phe150**, **Val151**, **Thr154**, **Ser155**, Pro157, **Phe158**, Val186, **Leu189**, **His190**, **Val192**, **Ser193**, **Val196**, **Gly197**, **Tyr249**, **Arg253**, Leu257	19	16
		AutoVina	-9.2	**Tyr104**, **Tyr108**, **Phe112**, **Phe150**, **Val151**, **Thr154**, **Ser155**, **Phe158**, **Leu189**, **His190**, **Val192**, **Ser193**, **Val196**, **Gly197**, **Tyr249**, **Arg253**	16	
Apigenin-7-O-glucoside	-8.263	MOE	-7.326	**Tyr104**, **Tyr108**, **Thr154**, **Ser155**, **Phe158**, **Phe174**, **Glu175**, **Pro176**, **Pro177**, **Val186**, **Leu189**, **His190**, **Ser193**, **Tyr249**, **Arg253**, **Leu257**, **Val277**, **Leu281**	18	18
		AutoVina	-9.2	**Tyr104**, **Tyr108**, Val151, **Thr154**, **Ser155**, **Phe158**, **Phe174**, **Glu175**, **Pro176**, **Pro177**, **Val186**, **Leu189**, **His190**, **Ser193**, **Tyr249**, **Arg253**, **Leu257**, **Val277**, **Leu281**	19	

The residues in bold are the residues that form interactions with the molecule in both software.

Table S9. The affinity values and residues of CRTH2 that interacted with Fevipiprant and the best 15 molecules, with both software.

Phytochemical	Mean Value of Affinity	Software	Affinity Value	Residues	Number of residues forming interactions	Number of repeated residues
Neoxanthin	-9.529	MOE	-9.158	**Leu20**, **Gln21**, **Ser22**, **Phe87**, **Phe90**, **His95**, **His107**, **Ser108**, **Phe111**, **Phe112**, **Arg170**, **Arg175**, Met181, Cys182, **Tyr183**, Tyr184, Tyr262, **Trp283**, **Arg284**, **Pro287**, **Thr290**	21	17
		AutoVina	-9.9	**Leu20**, **Gln21**, **Ser22**, Tyr30, **Phe87**, **Phe90**, **His95**, **His107**, **Ser108**, **Phe111**, **Phe112**, **Arg170**, **Arg175**, Arg179, **Tyr183**, **Trp283**, **Arg284**, Leu286, **Pro287**, **Thr290**, Phe294	21	
Alpha-tocotrienol	-9.258	MOE	-8.817	**Met17**, Leu20, Ser22, Tyr30, **Phe87**, **Phe90**, His95, Trp97, **His107**,	21	13

				**Ser108**, **Phe111**, **Phe112**, **Arg170**, **Arg175**, Arg179, Met181, **Cys182**, **Tyr183**, **Trp283**, **Pro287**, Thr290		
		AutoVina	-9.7	**Met17**, **Phe87**, **Phe90**, **His107**, **Ser108**, **Phe111**, **Phe112**, **Arg170**, **Arg175**, **Cys182**, **Tyr183**, Tyr184, Thr190, Tyr262, Val282, **Trp283**, **Pro287**, Leu286, Phe294	19	
Fevipiprant	-9.207	MOE	-7.614	**Met17**, **Phe87**, **Phe90**, **His107**, **Ser108**, **Phe111**, **Phe112**, **Arg170**, **Arg175**, **Met181**, **Cys182**, **Tyr183**, **Tyr184**, **Lys210**, **Tyr262**, **Pro287**, **Thr290**	17	17
		AutoVina	-10.8	**Met17**, **Phe87**, **Phe90**, **His107**, **Ser108**, **Phe111**, **Phe112**, **Arg170**, **Arg175**, **Met181**, **Cys182**, **Tyr183**, **Tyr184**, **Lys210**, **Tyr262**, Leu286, **Pro287**, **Thr290**	18	
Ursolic acid	-8.958	MOE	-5.617	Met17, Leu20, **Phe87**, **Phe90**, **His95**, **His107**, **Ser108**, **Phe111**, **Phe112**, **Arg170**, **Arg175**, **Cys182**, **Tyr183**, **Trp283**, Leu286, **Pro287**, **Thr290**, **Phe294**	18	15
		AutoVina	-12.3	**Phe87**, **Phe90**, **His95**, **His107**, **Ser108**, **Phe111**, **Phe112**, **Arg170**, **Arg175**, **Cys182**, **Tyr183**, Tyr262, **Trp283**, **Pro287**, **Thr290**, **Phe294**	16	
Integerrimine	-8.909	MOE	-6.819	**Phe87**, **Phe90**, **His107**, **Ser108**, **Phe111**, **Arg170**, **Cys182**, **Tyr183**, **Tyr184**, **Tyr262**, **Leu286**, **Pro287**, **Thr290**, **Phe294**	14	14
		AutoVina	-11	**Phe87**, **Phe90**, **His107**, **Ser108**, **Phe111**, Phe112, **Arg170**, **Cys182**, **Tyr183**, **Tyr184**, **Tyr262,** **Leu286**, **Pro287**, **Thr290**, **Phe294**	15	
Amentoflavone	-8.880	MOE	-7.960	**Met17**, **Leu20**, **His39**, **Phe87**, **Phe90**, **Leu91**, **His95**, Phe111, Phe112, Arg170, **Arg175**, **Met181**, **Cys182**, **Tyr183**, Lys210, Tyr262, **Trp283**, **Leu286**, **Pro287**, Thr290, **Ser291**	21	15
		AutoVina	-9.8	**Met17**, **Leu20**, **His39**, **Phe87**, **Phe90**, **Leu91**, **His95**, Trp97, His107, **Arg175**, **Met181**, **Cys182**, **Tyr183**, Val282, **Trp283**, **Leu286**, **Pro287**, **Ser291**	18	
β-carotene	-8.868	MOE	-9.036	**Gln21**, **Ser22**, **His23**, **Phe87**, His95, **His107**, **Ser108**, **Phe111**, **Phe112**, **Arg170**, **Arg175**, Arg179, **Tyr183**, **Trp283**, **Pro287**, **Thr290**, **Phe294**	17	15
		AutoVina	-8.7	Met17, Leu20, **Gln21**, **Ser22**, **His23**, **Phe87**, Phe90, **His107**, **Ser108**, **Phe111**, **Phe112**, **Arg170**, **Arg175**, Cys182, **Tyr183**, **Trp283**, Arg284, **Pro287**, **Thr290**, **Phe294**	20	
9-amino-camptothecin	-8.853	MOE	-7.407	**Met17**, **Phe90**, **His107**, **Ser108**, Phe111, Phe112, **Arg170**, **Arg175**, Met181, **Cys182**, **Tyr183**, Tyr262, **Leu286**, **Pro287**, **Thr290**, Phe294	16	11
		AutoVina	-10.3	**Met17**, Phe87, **Phe90**, Trp97, **His107**, **Ser108**, **Arg170**, **Arg175**, **Cys182**, **Tyr183**, Val282, Trp283, **Leu286**, **Pro287**, **Thr290**	15	
Hecogenin	-8.852	MOE	-7.105	**Met17**, **Leu20**, **Phe87**, **Phe90**, **His107**, **Ser108**, **Phe111**, **Arg170**, **Arg175**, **Cys182**, **Tyr183**, **Trp283**, **Leu286**, **Pro287**, **Thr290**, **Phe294**	16	16
		AutoVina	-10.6	**Met17**, **Leu20**, **Phe87**, **Phe90**, His95, **His107**, **Ser108**, **Phe111**, **Arg170**,	17	

				**Arg175**, **Cys182**, **Tyr183**, **Trp283**, **Leu286**, **Pro287**, **Thr290**, **Phe294**		
Kaempferol rhamnoside	-8.777	MOE	-7.855	Met17, **Phe87**, **Phe90**, **Trp97**, **His107**, Ser108, Phe111, **Phe112**, **Arg170**, **Arg175**, Met181, **Cys182**, **Tyr183**, **Tyr262**, **Trp283**, **Leu286**, **Pro287**, **Thr290**, Phe294	19	14
		AutoVina	-9.7	**Phe87**, **Phe90**, His95, **Trp97**, **His107**, **Phe112**, **Arg170**, **Arg175**, **Cys182**, **Tyr183**, Tyr184, **Tyr262**, Val282, **Trp283**, **Leu286**, **Pro287**, **Thr290**	17	
Cholecalciferol	-8.705	MOE	-7.71	Leu20, **Phe87**, **Phe90**, His95, **His107**, **Ser108**, **Phe111**, Phe112, **Arg170**, **Arg175**, Arg179, **Met181**, **Cys182**, **Tyr183**, **Trp283**, Arg284, **Leu286**, **Pro287**, **Thr290**, **Phe294**	20	15
		AutoVina	-9.7	Met17, **Phe87**, **Phe90**, Trp97, **His107**, **Ser108**, **Phe111**, **Arg170**, **Arg175**, **Met181**, **Cys182**, **Tyr183**, Tyr262, **Trp283**, **Leu286**, **Pro287**, **Thr290**, **Phe294**	18	
Solanidine	-8.703	MOE	-5.707	Met17, **Phe87**, **Phe90**, **His95**, His105, **Ser108**, **Phe111**, **Arg170**, **Arg175**, **Cys182**, **Tyr183**, Val282, **Trp283**, **Leu286**, **Pro287**, **Thr290**, **Phe294**	17	14
		AutoVina	-11.7	**Phe87**, **Phe90**, **His95**, His107, **Ser108**, **Phe111**, Phe112, **Arg170**, **Arg175**, **Cys182**, **Tyr183**, Tyr262, **Trp283**, **Leu286**, **Pro287**, **Thr290**, **Phe294**	17	
Isorhamnetin 3-O-rutinoside	-8.698	MOE	-8.497	**Leu20**, Ser22, **Phe87**, **Phe90**, **Leu91**, **His95**, **Ser108**, Phe112, **Arg170**, **Arg175**, Arg179, **Met181**, **Cys182**, **Tyr183**, Tyr184, Tyr262, **Trp283**, **Arg284**, Leu286, **Pro287**, **Thr290**	21	15
		AutoVina	-8.9	Met17, **Leu20**, His39, **Phe87**, **Phe90**, **Leu91**, **His95**, Trp97, **Ser108**, **Arg170**, **Arg175**, **Met181**, **Cys182**, **Tyr183**, **Trp283**, **Arg284**, **Pro287**, Phe288, **Thr290**, Ser291	20	
Isorhamnetin rutinoside	-8.620	MOE	-7.641	Met17, **Phe87**, **Phe90**, **Leu91**, **His95**, **His107**, **Ser108**, Phe111, Phe112, **Arg170**, **Arg175**, Met181, **Cys182**, **Tyr183**, **Trp283**, **Pro287**, **Thr290**, **Ser291**, **Phe294**	19	15
		AutoVina	-9.6	Leu20, His39, **Phe87**, **Phe90**, **Leu91**, **His95**, **His107**, **Ser108**, **Arg170**, **Arg175**, Arg179, **Cys182**, **Tyr183**, **Trp283**, **Pro287**, **Thr290**, **Ser291**, **Phe294**	18	
Isorhamnetin-3-O-glucoside	-8.611	MOE	-8.423	**Met17**, **Phe87**, **Phe90**, **His95**, **Trp97**, **His107**, **Ser108**, **Phe111**, **Phe112**, **Arg170**, **Arg175**, **Cys182**, **Tyr183**, **Tyr184**, **Tyr262**, **Trp283**, **Leu286**, **Pro287**, **Thr290**, **Phe294**	20	20
		AutoVina	-8.8	**Met17**, **Phe87**, **Phe90**, **His95**, **Trp97**, **His107**, **Ser108**, **Phe111**, **Phe112**, **Arg170**, **Arg175**, **Cys182**, **Tyr183**, **Tye184**, **Tyr262**, **Trp283**, **Leu286**, **Pro287**, **Thr290**, **Phe294**	20	
Epicatechin gallate	-8.610	MOE	-7.621	Met17, **Phe87**, **Phe90**, His95, **His107**, **Ser108**, **Phe111**, **Phe112**, **Arg170**, **Arg175**, **Met181**, **Cys182**, **Tyr183**, **Tyr184**, **Tyr262**, Leu286, **Pro287**, **Thr290**, **Phe294**	19	16

		AutoVina	-9.6	**Phe87**, **Phe90**, **His107**, **Ser108**, **Phe111**, **Phe112**, **Arg170**, **Arg175**, **Met181**, **Cys182**, **Tyr183**, **Tyr184**, Lys210, **Tyr262**, Ser266, Glu269, Leu285, **Pro287**, **Thr290**, **Phe294**	20	

The residues in bold are the residues that form interactions with the molecule in both software.

Table S10. The affinity values and residues of BK2R that interacted with JSM10292 and the best 15 molecules, with both software.

Phytochemical	Mean Value of Affinity	Software	Affinity Value	Residues	Number of residues forming interactions	Number of repeated residues
Isorhamnetin rutinoside	-8.718	MOE	-7.937	**Trp113**, **Phe121**, **Asn134**, **Ile137**, Ser138, **Leu141**, Met192, **Arg196**, **Val212**, **Ile213**, Glu221, Thr224, Asn225, Leu228, Phe286, Thr290, Asp293, Arg297, **Ser318**, **Tyr322**	20	10
		AutoVina	-9.5	Leu53, **Trp113**, Ile117, **Phe121**, **Asn134**, **Ile137**, **Leu141**, **Arg196**, Tyr201, Glu204, Ala210, Cys211, **Val212**, **Ile213**, Asp311, Thr314, Gln315, **Ser318**, Phe319, **Tyr322**	20	
Heptadecanoic acid	-8.647	MOE	-6.395	**Leu53**, **Trp113**, **Phe121**, Ile137, Leu141, **Tyr201**, **Glu204**, Trp283, Phe286, Asp311, **Thr314**, **Gln315**, Ser318, Phe319, Ala321, Tyr322	16	7
		AutoVina	-10.9	**Leu53**, **Trp113**, **Phe121**, Arg196, **Tyr201**, **Glu204**, Ala210, Cys211, Val212, Asp311, **Thr314**, **Gln315**	12	
7β-Hydroxy-sitosterol	-8.645	MOE	-7.49	Leu53, **Trp113**, **Phe121**, Asn134, **Ile137**, Ser138, Met192, Arg196, **Tyr201**, **Glu204**, **Phe286**, **Asp311**, **Thr314**, **Gln315**, **Ser318**, **Phe319**, **Tyr322**	17	12
		AutoVina	-9.8	**Trp113**, **Phe121**, **Ile137**, **Tyr201**, **Glu204**, Ala210, Val212, **Phe286**, **Asp311**, **Thr314**, **Gln315**, **Ser318**, **Phe319**, **Tyr322**	14	
γ-sitosterol	-8.603	MOE	-7.507	Leu106, **Ile137**, **Leu141**, Met192, **Arg196**, Ile213, Tyr215, Gl221, Thr224, Asn225, **Phe286**, Asp293, Arg297, **Ser318**, Ala321, **Tyr322**,	16	6
		AutoVina	-9.7	Trp113, Phe121, **Ile137**, **Leu141**, **Arg196**, Tyr201, Glu204, Ala210, Val212, **Phe286**, **Ser318**, Phe319, **Tyr322**	13	
7α-Hydroxy sitosterol	-8.569	MOE	-7.038	**Trp113**, **Phe121**, **Ile137**, Ser138, Leu141, Met192, Leu228, **Phe286**, Asp293, Arg297, **Asp311**, **Thr314**, **Gln315**, **Ser318**, **Phe319**, **Tyr322**,	16	10
		AutoVina	-10.1	**Trp113**, **Phe121**, **Ile137**, Tyr201, Glu204, Ala210, Val212, **Phe286**, **Asp311**, **Thr314**, **Gln315**, **Ser318**, **Phe319**, **Tyr322**	14	
Amentoflavone	-8.384	MOE	-7.769	Leu53, **Trp113**, **Phe121**, **Asn134**, **Ile137**, **Met192**, **Arg196**, Tyr201,	20	15

				Glu204, Val212, Asn225, **Leu228**, **Phe286**, **Thr290**, **Asp293**, **Thr314**, **Gln315**, **Ser318**, **Phe319**, **Tyr322**		
		AutoVina	-9	**Trp113**, **Phe121**, **Asn134**, **Ile137**, Ser138, **Met192**, **Arg196**, **Leu228**, **Phe286**, Ser289, **Thr290**, **Asp293**, Asp311, **Thr314**, **Gln315**, **Ser318**, **Phe319**, **Tyr322**	18	
JSM-10292	-8.375	MOE	-7.450	**Trp113**, **Phe121**, **Asn134**, Ser138, Leu141, **Met192**, **Arg196**, Tyr201, Ala210, Cys211, Val212, **Thr224**, **Asn225**, **Leu228**, **Asp293**, Asp311, Thr314, Gln315, Ser318	19	9
		AutoVina	-9.3	**Trp113**, **Phe121**, **Asn134**, **Met192**, **Arg196**, Ile213, Tyr215, Glu221, **Thr224**, **Asn225**, **Leu228**, Phe286, Thr290, **Asp293**, Arg297, Ser318, Phe319, Tyr322	18	
Kaempferol-3-rutinoside	-8.278	MOE	-8.657	Leu53, Phe121, **Arg196**, Tyr201, Glu204, Val212, Ile213, Tyr215, Glu221, **Thr224**, **Asn225**, **Asp293**, Arg297, Asp311, **Thr314**, Gln315	16	5
		AutoVina	-7.9	Trp113, Asn134, Ile137, Ser138, Leu141, Met192, **Arg196**, **Thr224**, **Asn225**, Leu228, Trp283, Phe286, Thr290, **Asp293**, **Thr314**, Ser318, Ala321, Tyr322	18	
Bioallethrin	-8.197	MOE	-6.494	**Trp113**, **Ile117**, **Phe121**, Asn134, Ile137, Leu141, Ser138, Met192, Arg196, Phe286, **Thr314**, **Gln315**, **Ser318**, **Phe319**, Ala321, **Tyr322**	16	8
		AutoVina	-9.9	**Trp113**, **Ile117**, **Phe121**, Tyr201, Ala210, Cys211, Val212, **Thr314**, **Gln315**, **Ser318**, **Phe319**, **Tyr322**	12	
Isorhamnetin 3-O-rutinoside	-8.181	MOE	-8.863	**Trp113**, **Ile117**, **Phe121**, Asn134, Ile137, Leu141, Met192, **Arg196**, **Val212**, Ile213, Tyr215, Glu221, **Asn225**, **Leu228**, Trp283, **Phe286**, **Thr290**, **Asp293**, **Arg297**, **Gln315**, **Ser318**, **Phe319**, Ala321, **Tyr322**	24	15
		AutoVina	-7.5	**Trp113**, **Ile117**, **Phe121**, **Arg196**, Ala210, Cys211, **Val212**, **Asn225**, **Leu228**, **Phe286**, **Thr290**, **Asp293**, **Arg297**, Asp311, Thr314, **Gln315**, **Ser318**, **Phe319**, **Tyr322**	19	
Quercetin rhamnoside	-7.883	MOE	-6.666	**Trp113**, **Phe121**, Asn134, Ile137, Met192, Arg196, Ile213, Thr224, **Asn225**, **Leu228**, **Thr290**, **Asp293**, **Thr314**, Gln315, **Ser318**, **Phe319**, **Tyr322**	17	10
		AutoVina	-9.1	**Trp113**, Ile117, **Phe121**, **Asn225**, **Leu228**, Phe286, Ser289, **Thr290**, **Asp293**, Arg297, Asp311, **Thr314**, **Ser318**, **Phe319**, **Tyr322**	15	
Solanidine	-7.7819	MOE	-6.263	**Trp113**, **Asn134**, Leu141, **Arg196**, Ile213, Tyr215, Glu221, Asn225, Leu228, Trp283, Phe286, Asp293, Arg297, **Ser318**, Ala321, **Tyr322**	16	5
		AutoVina	-9.3	**Trp113**, Phe121, **Asn134**, Ile137, Ser138, Met192, **Arg196**, Tyr201, Asp311, Thr314, Gln315, **Ser318**, Phe319, **Tyr322**	14	
Piperine	-7.773	MOE	-5.646	**Ile137**, Ser138, Leu141, Met192, **Arg196**, Ile213, Tyr215, Glu221, Thr224, Asn225, Leu228, Phe286, Asp293, Arg297, **Ser318**, Ala321, **Tyr322**	17	4

		AutoVina	-9.9	Trp113, Phe121, Asn134, **Ile137**, **Arg196**, Tyr201, Glu204, Asp311, Gln315, **Ser318**, Phe319, **Tyr322**	12	
Quercetin dihexoside	-7.770	MOE	-7.94	Leu53, **Trp113**, **Phe121**, **Asn134**, **Ile137**, **Ser138**, **Leu141**, **Met192**, **Arg196**, **Tyr201**, Glu204, Ile213, Tyr215, Asn225, Leu228, Phe285, Thr290, **Asp293**, **Asp311**, **Thr314**, Gln315, **Ser318**	22	13
		AutoVina	-7.6	**Trp113**, **Phe121**, **Asn134**, **Ile137**, **Ser138**, **Leu141**, **Met192**, **Arg196**, **Tyr201**, Ala210, Phe286, **Asp293**, Arg297, Ile310, **Asp311**, **Thr314**, **Ser318**, Phe319	18	
Isorhamnetin-3-O-glucoside	-7.680	MOE	-6.661	**Trp113**, **Phe121**, **Asn134**, **Ile137**, **Met192**, Ala210, Cys211, Val212, Ile213, Tyr215, **Thr224**, **Asn225**, **Asp293**, Arg297, **Thr314**, **Gln315**, **Ser318**, **Phe319**, Tyr322	19	12
		AutoVina	-8.7	**Trp113**, Ile117, **Phe121**, **Asn134**, **Ile137**, Ser138, Leu141, **Met192**, Arg196, **Thr224**, **Asn225**, Leu228, Phe286, Ser289, Thr290, **Asp293**, **Thr314**, **Gln315**, **Ser318**, **Phe319**	20	
Cholecalciferol	-7.596	MOE	-7.392	**Trp113**, **Ile117**, **Phe121**, Asn134, **Ile137**, Leu141, Arg196, **Tyr201**, Glu204, **Phe286**, **Asp311**, **Thr314**, **Gln315**, **Ser318**, **Phe319**, Ala321, **Tyr322**	17	12
		AutoVina	-7.8	Leu53, **Trp113**, **Ile117**, **Phe121**, **Ile137**, **Tyr201**, **Phe286**, **Asp311**, **Thr314**, **Gln315**, **Ser318**, **Phe319**, **Tyr322**	13	

The residues in bold are the residues that form interactions with the molecule in both software.

Table S11. UD phytochemicals mean values against the 5 receptors. Values in kcal/mol.

Phytochemical	PubChem CID	HR1	NKR1	CLR1	CRTH2	BK2R	Mean Value	Deviation
Amentoflavone	5281600	-6.3622	-9.6049	-9.5653	-8.8802	-8.3849	-8.5595	1.3291
Alpha-tocotrienol	5282347	-7.3107	-8.4025	-9.1082	-9.2589	-6.9592	-8.2079	1.0389
Neoxanthin	5282217	-6.8562	-7.9645	-8.8725	-9.5292	-7.1843	-8.0813	1.1227
7α-Hydroxy sitosterol	161816	-7.4651	-7.9638	-7.9392	-8.4453	-8.5691	-8.0765	0.4427
Isorhamnetin 3-O-rutinoside	5481663	-6.1382	-8.5197	-8.3735	-8.6986	-8.1819	-7.9824	1.0483
γ-sitosterol	457801	-7.3376	-7.7386	-7.6039	-8.3561	-8.6040	-7.9280	0.5314
Cholecalciferol	5280795	-7.2943	-7.6479	-8.1808	-8.7050	-7.5965	-7.8849	0.5586
Kaempferol-3-rutinoside	5318767	-5.9934	-8.4043	-8.4183	-8.1471	-8.2785	-7.8483	1.0427
Isorhamnetin rutinoside	133562525	-6.0691	-7.8149	-7.9169	-8.6208	-8.7188	-7.8281	1.0635
Epigallocatechin gallate	65064	-6.4312	-7.8261	-8.9232	-8.3555	-7.5162	-7.8104	0.9387
Hecogenin	91453	-7.0916	-7.9539	-8.0591	-8.8530	-6.8492	-7.7613	0.8059
Solanidine	65727	-7.2348	-7.3500	-7.7051	-8.7038	-7.7819	-7.7551	0.5784

7β-Hydroxy-sitosterol	12309569	-5.9741	-7.7017	-7.8675	-8.4825	-8.6450	-7.7342	1.0614
Dicaffeoylquinic acid	12358846	-6.5190	-8.1286	-8.2536	-8.5119	-7.2052	-7.7236	0.8346
Epicatechin gallate	107905	-6.4581	-7.3296	-8.9389	-8.6107	-7.1807	-7.7036	1.0385
Isorhamnetin-3-O-glucoside	5318645	-5.7953	-8.3667	-8.0454	-8.6119	-7.6810	-7.7000	1.1207
Beta-Sitosterol	222284	-7.3879	-7.5666	-7.5114	-8.5866	-7.3827	-7.6870	0.5091
Quercetin dihexoside	5320835	-6.2730	-8.4205	-7.7118	-8.2362	-7.7700	-7.6823	0.8436
Quercetin rhamnoside	15939939	-5.8567	-7.9486	-8.2821	-8.2158	-7.8835	-7.6373	1.0098
Apigenin-7-O-glucoside	44257792	-6.8922	-7.7854	-8.2634	-8.4462	-6.7872	-7.6349	0.7658
Sitosterol-β-D-glucoside	91884650	-6.4003	-7.9531	-8.7461	-8.2148	-6.8304	-7.6289	0.9802
Phytosterols	12303662	-7.3311	-7.5270	-7.8672	-8.5865	-6.7591	-7.6142	0.6761
Peonidin 3-O-rutinoside	44256842	-5.5160	-8.5767	-8.4791	-8.5170	-6.7149	-7.5607	1.3862
Quercitrin	5280459	-5.5743	-8.4552	-7.9197	-8.1540	-7.3266	-7.4859	1.1461
9-amino-camptothecin	72402	-5.2801	-7.4164	-8.2912	-8.8538	-7.3328	-7.4348	1.3602
Kaempferol rhamnoside	5835713	-5.7680	-7.7362	-7.6518	-8.7779	-7.2266	-7.4321	1.0912
Quercetin-3-O-glucoside	25203368	-5.6563	-8.1769	-7.7072	-8.3310	-7.2812	-7.4305	1.0741
Kaempferol-3-O-glucoside	5282102	-5.6214	-7.9124	-7.5151	-8.5024	-7.5620	-7.4226	1.0815
Quercetin-3-glucoside	5280804	-5.5699	-8.1405	-7.4166	-8.4531	-7.2935	-7.3747	1.1197
Bioallethrin	15558638	-5.4565	-7.1724	-7.6128	-8.3146	-8.1972	-7.3507	1.1548
Piperine	638024	-6.7045	-6.9975	-7.3059	-7.6828	-7.7730	-7.2927	0.4517
Caffeoyl feruloyl tartaric acid	129724266	-6.3052	-7.8550	-7.7324	-7.9911	-6.5747	-7.2917	0.7887
10-gingerdione	5317591	-6.5775	-7.1963	-7.3408	-8.2780	-6.9098	-7.2605	0.6394
6-aminochrysene	17534	-6.6466	-7.2198	-7.4682	-7.7445	-7.1307	-7.2420	0.4096
Pukateine	442340	-6.1982	-7.3474	-7.6025	-8.5737	-6.3647	-7.2173	0.9709
(+)-Neo-olivil	9976812	-5.8546	-7.0561	-7.8146	-8.3863	-6.8927	-7.2008	0.9631
Chlorogenic acid	1794427	-6.6335	-7.0618	-7.4535	-8.1129	-6.5466	-7.1616	0.6437
Isolariciresinol	160521	-5.9928	-7.1439	-7.4408	-7.9258	-7.2821	-7.1571	0.7146
Kaempferol pentoside	14749097	-5.5959	-7.4915	-7.2946	-8.3687	-6.9992	-7.1500	1.0075
Ursolic acid	64945	-5.9003	-7.1326	-7.2937	-8.9587	-6.2865	-7.1143	1.1825
4-O-Caffeoylquinic acid	58427569	-6.4796	-7.0126	-7.1985	-7.9074	-6.7805	-7.0757	0.5370
Deoxyharringtonine	285342	-5.8281	-7.2078	-7.7410	-8.5847	-5.8720	-7.0467	1.1977
8-dehydrogingerdione	131752598	-6.6981	-6.4452	-7.1146	-8.0165	-6.9392	-7.0427	0.6000
Incensole oxide acetate	73755086	-5.9571	-6.8593	-7.3097	-8.0449	-6.8920	-7.0126	0.7593
Neochlorogenic acid	5280633	-6.1686	-6.9089	-7.2051	-7.8653	-6.8059	-6.9907	0.6180
Apoatropine	64695	-6.3308	-6.6831	-7.1208	-7.5152	-7.2744	-6.9848	0.4750
Carnosol	442009	-6.5238	-6.4984	-7.6619	-8.2736	-5.8882	-6.9691	0.9708
3,4-dimethoxychalcone	5354494	-6.6114	-6.7287	-7.4917	-7.3168	-6.5988	-6.9495	0.4228
[1,1-Bicyclopropyl-2-octanoic acid 2hexyl-methyl ester	50930793	-6.2276	-6.9148	-6.9217	-7.9675	-6.5037	-6.9070	0.6613
Estra-1,3,5(10)-trien-17B-ol	9811784	-6.5498	-7.1896	-7.3148	-7.4950	-5.8608	-6.8820	0.6728
Integerrimine	5281733	-6.2116	-6.4280	-7.1716	-8.9099	-5.6787	-6.8799	1.2549
Farnesylacetone	1711945	-6.3866	-6.7844	-6.9455	-7.7011	-6.5617	-6.8758	0.5080

Caffeoylquinic acid	10155076	-6.4479	-6.8281	-7.0850	-7.4892	-6.4996	-6.8699	0.4321
Neophytadiene	10446	-6.1466	-6.2363	-7.0023	-7.6571	-7.3071	-6.8698	0.6620
Incensole oxide	90470329	-6.3100	-6.7215	-7.1159	-8.3293	-5.8281	-6.8609	0.9501
Isopilosine	72312	-6.2071	-6.8846	-6.7655	-7.4039	-6.9779	-6.8478	0.4315
1,2-Diguaiacyl-1,3-propanediol	6426042	-5.7753	-6.8790	-7.2153	-7.5157	-6.8443	-6.8459	0.6581
Heptadecanoic acid	10465	-5.7894	-5.8944	-6.5392	-7.3154	-8.6476	-6.8372	1.1813
Luteolin	5280445	-6.1779	-7.0635	-7.1435	-7.3755	-6.3256	-6.8172	0.5313
Dihydrokavain	10220256	-6.1289	-6.3316	-6.9994	-7.1107	-7.3745	-6.7890	0.5328
Catechin	9064	-5.6033	-6.7767	-7.2409	-7.3299	-6.7350	-6.7371	0.6878
Catechin hydrate	107957	-5.7423	-6.7530	-7.4298	-7.2184	-6.5386	-6.7364	0.6597
Eleutheroside B	5316860	-5.8462	-6.8671	-6.9259	-7.9283	-6.0931	-6.7321	0.8185
Podocarpic acid	93017	-6.0328	-6.5483	-6.8506	-7.4364	-6.7823	-6.7301	0.5088
Flavone	10680	-6.2877	-6.4600	-6.9261	-7.3831	-6.3565	-6.6827	0.4642
Osthole	10228	-5.6363	-6.6026	-6.9366	-7.3753	-6.7701	-6.6642	0.6426
Convolvamine	420422	-6.2133	-6.8045	-7.3396	-7.2265	-5.6852	-6.6538	0.6988
Isorhamnetin	5281654	-6.0470	-6.7048	-7.2750	-7.4925	-5.7265	-6.6491	0.7616
Phytol	5280435	-6.2047	-6.3331	-6.9966	-7.7746	-5.8430	-6.6304	0.7637
Arachidonic acid	444899	-6.3187	-6.6338	-6.8767	-7.9799	-5.2140	-6.6046	0.9977
β-Bisabolene	10104370	-5.9351	-6.4944	-6.7929	-7.1807	-6.4754	-6.5757	0.4583
Linolenic acid	5280934	-6.2521	-6.4886	-6.5454	-7.6704	-5.8759	-6.5665	0.6710
Dibutyl phthalate	3026	-5.3436	-6.3070	-6.5974	-7.2289	-7.3363	-6.5626	0.8055
9-Hydroxy-10,12-octadecadienoic acid	1927	-5.9247	-6.4005	-6.6195	-7.5051	-6.3579	-6.5615	0.5845
δ-Cadinene	441005	-5.8228	-6.6077	-6.6929	-7.0815	-6.5717	-6.5553	0.4570
Naringenin	932	-5.9078	-6.6512	-6.9378	-7.5576	-5.7045	-6.5518	0.7588
Crysoeriol	5280666	-6.0959	-6.9503	-7.2555	-7.5966	-4.7706	-6.5338	1.1319
Apigenin	5280443	-6.0489	-6.7676	-6.9276	-7.5516	-5.3286	-6.5248	0.8563
O-Feruloyl quinic acid	10177048	-5.2641	-7.0122	-7.2897	-7.7728	-5.2649	-6.5207	1.1786
Kaempferol	5280863	-5.8954	-6.4778	-7.0332	-7.4884	-5.7006	-6.5191	0.7524
Flavan	94156	-6.1529	-6.2548	-6.9350	-7.2553	-5.8413	-6.4878	0.5860
γ-Cadinene	92313	-5.7030	-6.6488	-6.4682	-7.1196	-6.4573	-6.4794	0.5104
Kavain	5281565	-5.8292	-6.0151	-6.7440	-6.6608	-7.1478	-6.4794	0.5450
Secoisolariciresinol	65373	-5.7888	-6.8865	-6.7604	-7.4772	-5.4568	-6.4739	0.8311
4-shogaol	9794897	-6.0727	-6.2321	-6.6070	-6.9925	-6.4399	-6.4688	0.3561
Isotachioside	15098566	-5.3638	-6.4890	-6.3345	-6.9741	-7.1811	-6.4685	0.7075
Caffeoylmalic acid	6124299	-5.8538	-6.4262	-6.9698	-6.8770	-6.1951	-6.4644	0.4670
Caffeoyl tartaric acid	9857913	-5.7412	-6.6354	-6.5801	-6.7847	-6.5685	-6.4620	0.4120
Quinic acid	6508	-5.1402	-6.6492	-6.5813	-7.4150	-6.5218	-6.4615	0.8228
α-Copaene	442355	-5.8725	-6.2691	-6.7851	-6.8522	-6.4707	-6.4499	0.4002
Feruloyl malate	71694479	-5.8101	-6.5362	-7.0136	-7.0929	-5.7877	-6.4481	0.6298
α-Humulene	5281520	-5.8863	-6.6616	-6.1420	-7.2921	-6.2074	-6.4379	0.5533
Myricetin	5281672	-5.7343	-6.6571	-7.0894	-7.3220	-5.3530	-6.4311	0.8553
Abscisic acid	5375199	-5.6874	-6.4988	-6.3993	-7.2107	-6.3533	-6.4299	0.5414

Quercetin	5280343	-5.7511	-6.4868	-7.2135	-7.4806	-5.1994	-6.4263	0.9613
cis-10-Heptadecenoic acid	5312435	-5.8718	-6.1868	-6.4549	-7.4693	-6.1430	-6.4251	0.6192
Hexahydrofarnesylacetone	10408	-5.8428	-6.4893	-6.4975	-7.7354	-5.4361	-6.4002	0.8718
Genistein	5280961	-5.7754	-6.6600	-6.9841	-7.4101	-5.1651	-6.3989	0.9143
6-benzylaminopurine	62389	-5.7938	-6.2981	-6.8932	-6.6549	-6.3148	-6.3909	0.4162
β - Vetivenene	14475467	-5.8801	-6.0860	-6.6519	-6.9783	-6.3451	-6.3883	0.4385
β-Selinene	442393	-5.7991	-5.9835	-6.5378	-6.8892	-6.7091	-6.3837	0.4709
β - Sesquiphellandrene	519764	-6.1487	-6.0864	-6.5165	-6.7504	-6.2213	-6.3446	0.2804
Isopropyl dodecanoate	25068	-5.7310	-6.1037	-6.2993	-6.6213	-6.9638	-6.3438	0.4734
Anthocyanins	145858	-6.2222	-6.3687	-6.8287	-7.3901	-4.9083	-6.3436	0.9228
Calamenene	6429077	-5.3714	-6.3695	-6.4727	-7.0989	-6.3900	-6.3405	0.6195
ar-Curcumene	92139	-5.9891	-6.1173	-6.5559	-7.0560	-5.9418	-6.3320	0.4718
α-Selinene	10856614	-5.7665	-5.9521	-6.4786	-7.0541	-6.4046	-6.3312	0.5031
β-Bourbonene	62566	-5.5712	-6.4420	-6.4172	-6.9849	-6.2395	-6.3309	0.5082
2H-Indeno[1,2-b]furan-2-one, 3,3a,4,5,6,7,8,8b-octahydro-8,8-dimethyl	605626	-5.7166	-6.7316	-6.1499	-6.9409	-6.0810	-6.3240	0.5012
Homatropine	5282593	-6.1455	-6.6355	-6.6187	-7.2388	-4.8487	-6.2974	0.8980
Methyl palmitate	8181	-5.6972	-6.0816	-6.5060	-6.9977	-6.1495	-6.2864	0.4904
Pterostilbene	5281727	-6.1096	-6.3931	-6.6795	-7.2684	-4.9697	-6.2840	0.8507
Benzyl salicilate	8363	-5.9221	-6.1509	-7.0944	-7.0630	-5.1424	-6.2745	0.8239
Farnesol	445070	-5.8619	-6.3446	-6.5692	-6.7576	-5.7954	-6.2657	0.4256
β-Caryophyllene	5281515	-5.7762	-6.1732	-6.0858	-7.0162	-6.2386	-6.2580	0.4594
Rutin	5280805	-5.3880	-6.0581	-6.4438	-6.9121	-6.4303	-6.2465	0.5675
Palmitic acid	985	-5.9119	-6.0657	-6.2614	-6.9352	-6.0105	-6.2369	0.4106
Tachioside	11962143	-5.2972	-6.6465	-6.3336	-7.1652	-5.4803	-6.1845	0.7875
α- Copaene-8-ol	25086830	-5.8932	-6.1527	-6.1982	-6.9240	-5.7320	-6.1800	0.4576
p-Coumaroylmalic acid	129720114	-5.3350	-6.1971	-6.6970	-6.8783	-5.7354	-6.1685	0.6454
Lutein	5281243	-0.2712	-7.8756	-9.1613	-7.8382	-5.6723	-6.1637	3.5243
Caryophyllene oxide	1742210	-5.6242	-6.0024	-6.3045	-6.9194	-5.9547	-6.1610	0.4878
Myristic acid	11005	-5.5500	-5.8020	-5.9892	-6.6023	-6.8316	-6.1550	0.5422
4-(4-Hydroxy-2,6,6-trimethyl1-cyclohexen-1-yl)-3-buten2-one	538953	-5.2198	-6.4290	-6.2392	-6.6031	-6.2536	-6.1489	0.5402
Palmitoleic acid	445638	-5.8937	-5.8311	-6.1812	-6.9736	-5.6997	-6.1159	0.5108
Arbutine	440936	-5.5010	-6.0119	-6.1661	-6.6069	-6.2588	-6.1089	0.4040
Safranal	61041	-5.1985	-6.9771	-6.9494	-6.8424	-4.5233	-6.0981	1.1555
1,2-Benzenedicarboxylic acid	90531	-5.2133	-6.0589	-6.3786	-6.5379	-6.1890	-6.0755	0.5153
4-methyl-7-ethoxycoumarin	66595	-5.3936	-6.0342	-6.3645	-6.5461	-5.9986	-6.0674	0.4408
3-Hydroxy-damascone	5366075	-5.3113	-5.8678	-6.0572	-6.9024	-6.0497	-6.0377	0.5711
Myristoleic acid	5281119	-5.7930	-5.8311	-6.0624	-6.6046	-5.8673	-6.0317	0.3367
α-Longipinene	520957	-5.7151	-5.6431	-5.9151	-6.6508	-6.1037	-6.0055	0.4031
(E)-Geranyl acetone	1713001	-5.6101	-5.8823	-6.2529	-6.3513	-5.9178	-6.0029	0.3000

β-Ionone	638014	-5.2321	-6.0650	-6.0993	-6.6829	-5.9304	-6.0019	0.5184
4-(3-Hydroxy-1-butyn-1-yl)- 3,5,5-trimethyl-2-cyclohexen-1-ol	5280654	-5.3517	-5.5642	-6.2163	-6.6378	-6.0306	-5.9601	0.5140
Harmol	68094	-5.3526	-5.8927	-5.9489	-6.4950	-6.0545	-5.9487	0.4087
Geranyl acetone	1549778	-5.6919	-5.8844	-6.1236	-6.7178	-5.2669	-5.9369	0.5377
Carvacryl acetate	80792	-5.3688	-5.8990	-6.1173	-6.5844	-5.6583	-5.9255	0.4618
α-Ionone	5282108	-5.3409	-5.9716	-6.0411	-6.4843	-5.7416	-5.9159	0.4192
3-Oxo-a-ionol	5370052	-5.0972	-5.9972	-6.3270	-6.4564	-5.6400	-5.9035	0.5508
α- Terpinyl acetate	111037	-5.3116	-5.7212	-6.0232	-6.4705	-5.9521	-5.8957	0.4247
Harmine	5280953	-5.4396	-6.1734	-6.2831	-6.8679	-4.6331	-5.8794	0.8621
Carvylacetate	7335	-5.3149	-5.8927	-5.9939	-6.4713	-5.7205	-5.8787	0.4206
Epicatechin	72276	-5.6570	-6.7852	-7.3543	-3.2578	-6.2685	-5.8645	1.5865
Silane, triethyl(2-phenylethoxy)	610043	-5.2711	-5.6942	-6.1969	-5.9368	-6.1788	-5.8555	0.3858
4-methylmethoxycoumarin	223821	-5.3371	-5.8328	-6.0223	-6.2779	-5.7006	-5.8341	0.3526
Adenosine	60961	-5.3056	-6.0485	-6.4404	-6.6054	-4.6645	-5.8129	0.8145
Hydrocotarnine	3646	-5.2446	-6.0794	-5.9947	-6.4545	-5.2006	-5.7947	0.5504
Carvacrol	10364	-5.1289	-5.4227	-5.5043	-5.9286	-6.9369	-5.7843	0.7050
Shikimic acid	8742	-5.3374	-5.7985	-6.3755	-6.3123	-4.9967	-5.7641	0.6014
4-aminoantipyrine	2151	-5.1906	-5.5495	-6.0895	-6.3452	-5.5881	-5.7525	0.4606
Dodecendioic acid	5283028	-5.2954	-5.4128	-5.7982	-6.5128	-5.7271	-5.7492	0.4757
Phosphatidylcholine	10425706	-4.8376	-5.7325	-6.1749	-6.3384	-5.6098	-5.7386	0.5870
1-Dodecanamine, N, N-dimethyl	8168	-5.3214	-5.6511	-5.7060	-6.2390	-5.7228	-5.7280	0.3290
3,4-Dimethyl-5-pentylidene-2(5H)-furanone	6433214	-5.1422	-5.7655	-5.8252	-6.2006	-5.6683	-5.7203	0.3810
β-carotene	5280489	4.2123	-7.6869	-8.8746	-8.8681	-7.3603	-5.7155	5.5917
Phenylalanine	6140	-5.1290	-5.3283	-5.5109	-5.5373	-7.0157	-5.7042	0.7512
Esculetin	5281416	-4.9242	-5.3585	-5.6528	-5.9068	-6.6121	-5.6908	0.6315
Lotaustralin	441467	-4.8642	-5.6851	-5.5574	-6.4927	-5.7928	-5.6784	0.5817
Cinnamyl acetate	5282110	-5.2420	-5.5954	-5.9324	-6.0197	-5.5137	-5.6606	0.3178
Ferulic acid	445858	-4.8177	-5.5267	-5.8818	-6.0903	-5.9328	-5.6498	0.5088
Eugenol	3314	-4.6486	-5.6217	-5.6500	-5.6501	-6.5933	-5.6327	0.6877
3,4-Dimethyl-5-pentylfuran-2(5H)-one	13192443	-5.1268	-5.4865	-5.8485	-6.1177	-5.5396	-5.6238	0.3766
Vitamin B5	6613	-4.7431	-5.2418	-5.4048	-5.9301	-6.6212	-5.5882	0.7162
1-Methyl naphthalene	7002	-5.3832	-5.3720	-5.9389	-5.6152	-5.5235	-5.5666	0.2315
2(3H)-Naphthalenone, 4, 4a,5,6,7,8- hexahydro-1-methoxy	534313	-5.1183	-5.8116	-5.6172	-6.0140	-5.1719	-5.5466	0.3929
DL-methyl-m-tyrosine	2110	-5.2422	-5.6073	-5.7318	-6.0838	-5.0573	-5.5444	0.4058
Sinapyl alcohol	5280507	-4.5369	-5.5558	-5.7658	-6.1261	-5.6624	-5.5294	0.5949
Carvone	7439	-5.1828	-5.3002	-5.6138	-5.8639	-5.6723	-5.5266	0.2793

Methylcoumarin	17130	-5.1152	-5.5132	-5.8441	-6.0065	-5.0901	-5.5138	0.4154
Diphenyl	7095	-5.5218	-5.5660	-5.7659	-6.0304	-4.6810	-5.5130	0.5066
1,2-O-isopropylidene-D-glucoside	87704	-5.0145	-5.3462	-5.5310	-6.1776	-5.4104	-5.4959	0.4264
Salicylic alcohol	5146	-4.8123	-4.9981	-5.5048	-5.2264	-6.8679	-5.4819	0.8169
1-Hydroxy-1-(4-hydroxyphenyl)-2-propanone	10261435	-5.6043	-5.1653	-5.5189	-5.8058	-5.2763	-5.4741	0.2566
Bornyl acetate	6448	-5.6084	-5.0820	-5.5147	-6.0054	-5.0871	-5.4595	0.3887
Methyl eugenol	7127	-4.8827	-5.5823	-5.5052	-5.8331	-5.4938	-5.4594	0.3502
Vanillic acid	8468	-4.5669	-4.9937	-5.2518	-5.2792	-7.1638	-5.4511	0.9992
Caffeic acid	689043	-4.9517	-5.2185	-5.7374	-5.8378	-5.4156	-5.4322	0.3655
Lauric acid	3893	-5.2231	-5.2221	-5.6035	-6.0541	-5.0451	-5.4296	0.4043
Isopropyl-β-D-thio-galacto-pyranoside	656894	-4.7344	-5.2554	-5.6489	-5.9176	-5.5739	-5.4260	0.4530
Carnosine	439224	-5.0618	-5.3580	-5.1692	-6.1970	-5.2371	-5.4046	0.4558
Cumin aldehyde	326	-4.8067	-5.2423	-5.2117	-5.7608	-5.9757	-5.3994	0.4674
Sinapic acid	637775	-4.6197	-5.4570	-5.8421	-6.0595	-5.0047	-5.3966	0.5913
p-Cymene	7463	-4.9832	-5.2950	-5.2840	-5.5873	-5.8280	-5.3955	0.3226
Violaxanthin	448438	3.9458	-7.5934	-8.9456	-8.1983	-6.0962	-5.3775	5.3160
β-Homocyclocitral	61124	-4.8749	-5.5430	-5.3879	-5.8332	-5.2171	-5.3712	0.3582
Homovanillyl alcohol	16928	-4.6889	-5.1832	-5.3027	-5.3908	-6.2669	-5.3665	0.5719
Umbelliferone	5281426	-5.0417	-5.3074	-5.9450	-5.6618	-4.8053	-5.3522	0.4596
Coniferol	1549095	-4.7666	-5.4568	-5.7612	-5.9610	-4.7811	-5.3453	0.5518
Thymol	6989	-5.0208	-5.3568	-5.2675	-5.5347	-5.4401	-5.3240	0.1963
α- Terpineol	17100	-4.9173	-5.1544	-5.4499	-5.7234	-5.3045	-5.3099	0.3037
4-Acetyl-2-methylphenol	70135	-5.1517	-5.3063	-5.4874	-5.4362	-5.1324	-5.3028	0.1611
Cinnamide	5273472	-5.1799	-5.1574	-5.5014	-5.5725	-5.0874	-5.2997	0.2207
p-coumaric acid	637542	-4.7502	-5.1093	-5.4714	-5.7841	-5.2944	-5.2819	0.3875
Anthranilic acid methyl ester	8635	-4.9190	-4.7690	-5.3613	-5.2011	-6.0207	-5.2542	0.4872
Oxime- methoxy-phenyl	9602988	-4.7400	-5.0545	-5.3849	-5.2530	-5.7809	-5.2427	0.3867
Linalool	6549	-5.1607	-5.0813	-5.6415	-5.6818	-4.5482	-5.2227	0.4650
Hydroxycinnamaldehyde	71407359	-4.9221	-4.9348	-5.3165	-5.5450	-5.3611	-5.2159	0.2760
Camphor	2537	-5.0866	-4.4741	-5.1348	-5.4630	-5.8348	-5.1986	0.5040
β-Cyclocitral	9895	-4.7897	-5.2637	-5.3249	-5.5714	-5.0176	-5.1934	0.2996
Decan-2-one	12741	-4.7636	-4.9097	-5.1246	-5.6247	-5.4819	-5.1809	0.3669
Decanal	8175	-4.8324	-4.9359	-5.3154	-5.7518	-5.0496	-5.1770	0.3683
Syringic acid	10742	-4.4130	-5.2982	-5.5740	-5.4732	-5.1071	-5.1731	0.4606
n-acetyl-L-glutamine	182230	-5.0439	-4.9032	-5.2305	-5.4322	-5.2297	-5.1679	0.2019
(E)-Anethole	637563	-4.7835	-5.2422	-5.1679	-5.4717	-5.0976	-5.1526	0.2497
Borneol	64685	-4.9783	-4.4676	-4.8554	-5.3135	-6.0626	-5.1354	0.6001
Indole	798	-4.8180	-4.5620	-5.1387	-4.8917	-6.2571	-5.1335	0.6609
Synephrine	7172	-4.6730	-4.9305	-5.3140	-5.7253	-4.8661	-5.1018	0.4191
2-Pentylfuran	19602	-5.0310	-4.9303	-5.2244	-5.2771	-5.0127	-5.0951	0.1482

Naphthalene	931	-5.0007	-5.0522	-5.6491	-5.2235	-4.5188	-5.0888	0.4081
4-Vinyl guaiacol	332	-4.6287	-5.1831	-5.4477	-5.2251	-4.9309	-5.0831	0.3133
Gentisic acid	3469	-4.8036	-4.6925	-5.1136	-5.1030	-5.6952	-5.0816	0.3894
Scopoletin	5280460	-4.6832	-4.8160	-5.3984	-5.2053	-5.2824	-5.0770	0.3103
m-Hydroxy-acetophenone	8487	-4.7395	-4.8035	-5.2591	-5.3401	-5.1891	-5.0663	0.2753
2-(1-Pentenyl)furan	5369956	-4.7946	-4.8292	-5.2579	-5.4374	-5.0120	-5.0662	0.2772
2, 6,-Nonadienal, 3, 7-dimethyl	5364526	-4.4135	-4.8484	-5.9227	-5.2808	-4.8479	-5.0626	0.5703
Methyl chavicol	8815	-4.8676	-5.2022	-5.0431	-5.6513	-4.4474	-5.0423	0.4418
DL-a aminopimelic acid	101122	-4.5595	-4.5355	-5.0472	-5.3816	-5.6875	-5.0422	0.5053
Niacinamide	936	-4.4355	-4.2718	-4.8786	-4.7152	-6.7999	-5.0202	1.0225
Indole-3-carboxaldehyde	10256	-4.7967	-5.0215	-5.3429	-5.2731	-4.6393	-5.0147	0.3012
p-Hydroxy-acetophenone	7469	-4.7486	-4.8066	-5.2590	-5.1422	-4.9337	-4.9780	0.2179
Cinnamic acid	444539	-5.0207	-5.1500	-5.3667	-5.6071	-3.7079	-4.9704	0.7401
Citric acid	311	-4.8990	-4.4921	-5.1548	-5.2541	-5.0464	-4.9693	0.2976
L-2-aminoadipic acid	92136	-4.8207	-4.4815	-4.7437	-5.1447	-5.6235	-4.9628	0.4386
Tyrosol	10393	-4.5520	-4.7035	-5.2737	-5.0909	-5.1181	-4.9476	0.3050
Nonanol	8914	-4.7471	-4.6630	-5.0192	-5.3635	-4.8559	-4.9297	0.2766
Dimethyl-L-tartrate	11851	-4.1652	-4.5993	-4.7369	-5.2057	-5.9156	-4.9245	0.6669
Pyrogallol	1057	-4.3147	-4.2788	-4.8332	-4.8184	-6.3083	-4.9107	0.8250
2,4,6-Trimethyl-5H-1,3,5-dithiazine	12518	-4.8606	-4.8304	-4.3958	-5.4706	-4.9039	-4.8922	0.3827
Resorcinol	5054	-4.5554	-4.4482	-4.7618	-4.8494	-5.8235	-4.8876	0.5470
Acetophenone	7410	-4.8719	-4.7035	-5.1200	-5.0128	-4.6494	-4.8715	0.1995
Succinic acid	1110	-4.8364	-3.8311	-4.2310	-4.4772	-6.9623	-4.8676	1.2268
Phthalic acid	1017	-4.6535	-4.9183	-4.9317	-5.2531	-4.5637	-4.8641	0.2708
3,4-Dihydroxybenzoic acid	72	-4.8333	-4.6672	-5.1600	-5.0259	-4.5224	-4.8418	0.2587
Mannitol	6251	-4.1161	-4.4726	-4.6281	-5.1819	-5.8064	-4.8410	0.6623
Glucosamine	439213	-4.5563	-4.5802	-4.9389	-5.0571	-5.0481	-4.8361	0.2491
Nonanal	31289	-4.8495	-4.4232	-4.8488	-5.4732	-4.5540	-4.8297	0.4050
3-Octanone	246728	-4.9014	-4.5722	-4.9537	-5.1633	-4.5566	-4.8294	0.2611
4-deoxypyridoxine	6094	-4.5434	-4.7093	-5.1075	-4.9896	-4.7304	-4.8160	0.2281
2,6-Dimethoxy-hydroquinone	96038	-4.4394	-4.7970	-4.8829	-5.1682	-4.7463	-4.8068	0.2623
Aminobutyric acid	119	-4.7204	-3.6754	-4.1091	-4.1805	-7.1715	-4.7714	1.3921
Gallic acid	370	-4.5537	-4.7069	-5.1618	-5.1571	-4.2638	-4.7686	0.3906
Benzofuran, 2,3,-dihydro	10329	-4.8107	-4.6757	-5.1567	-4.9077	-4.2135	-4.7528	0.3490
n-Octanal	454	-4.7856	-4.5152	-4.7390	-5.1530	-4.5432	-4.7472	0.2558
5,6-Dihydro-4-pentyl- 2,6-dimethyl-4H-1,3,5-dithiazine	528360	-4.5530	-4.4662	-4.6909	-5.1043	-4.8001	-4.7229	0.2486
p-hydroxybenzoic acid	135	-4.8171	-4.4785	-5.1055	-4.9430	-4.2409	-4.7170	0.3520
Benzaldehyde	240	-4.4619	-4.1636	-4.7090	-4.5944	-5.4716	-4.6801	0.4871
Arabitol	94154	-4.2416	-4.1367	-4.4950	-4.4688	-6.0119	-4.6708	0.7648
p-Hydroxybenzyl alcohol	125	-4.5106	-4.4335	-5.1881	-4.6239	-4.5564	-4.6625	0.3019

Leucine	6106	-4.4909	-4.2681	-4.7724	-4.7699	-4.9745	-4.6551	0.2765
Heptanal	8130	-4.8824	-4.3856	-4.4354	-4.8045	-4.7542	-4.6524	0.2262
adenine	190	-4.3196	-4.2281	-4.7564	-4.4165	-5.4622	-4.6365	0.5029
Rhamnose	25310	-4.4924	-4.2429	-5.0173	-4.8088	-4.6036	-4.6330	0.2964
Proline	145742	-4.0063	-4.0830	-4.4314	-4.4834	-6.0778	-4.6164	0.8433
p-Hydroxy-benzaldehyde	126	-4.5205	-4.3241	-5.0289	-4.6824	-4.4840	-4.6079	0.2675
2-Heptanone	8051	-4.9034	-4.4818	-4.5563	-4.8214	-4.2625	-4.6050	0.2602
1,6-dioxaspiro[4.4]non-3-ene	10374471	-4.2796	-4.6823	-4.7223	-4.8609	-4.3975	-4.5885	0.2413
Anisaldehyde	31244	-4.4606	-4.6160	-4.7277	-5.1340	-3.9139	-4.5704	0.4437
Levoglucosan	2724705	-4.2356	-4.4146	-4.8533	-5.1606	-4.1164	-4.5561	0.4387
Levulinic acid	11579	-5.0129	-4.0932	-4.3727	-4.7745	-4.3198	-4.5146	0.3713
3-hydroxy-2,3 dihydromaltol	119838	-4.4129	-4.4033	-4.5699	-4.7173	-4.3373	-4.4881	0.1540
Hexanal	6184	-4.3494	-4.0535	-4.1694	-4.4552	-5.0756	-4.4206	0.3979
Histidinol	776	-4.6736	-4.0396	-4.4760	-4.7254	-4.0323	-4.3894	0.3358
Glutaric acid	743	-4.6733	-4.0851	-4.3199	-4.7147	-4.1178	-4.3821	0.2989
2-acetylpyrrole	14079	-4.7035	-4.0104	-4.5642	-4.5213	-4.0851	-4.3769	0.3091
Galactal	2734735	-4.2527	-4.3599	-4.8189	-4.9485	-3.4751	-4.3710	0.5811
Creatinine	588	-4.0338	-4.0479	-4.3710	-3.9831	-5.3583	-4.3588	0.5793
Choline	305	-3.5379	-3.4924	-3.8792	-3.7727	-6.9193	-4.3203	1.4618
2-deoxy-D-ribose	5460005	-4.6597	-3.9711	-4.3789	-4.5414	-3.8302	-4.2762	0.3605
1,4-benzoquinone	4650	-4.1461	-4.0304	-4.7595	-4.3478	-3.9999	-4.2567	0.3124
L-threonine	6288	-3.9644	-3.7595	-4.3687	-4.1956	-4.6826	-4.1942	0.3574
6-azacytosine	70265	-4.3448	-4.0075	-4.4565	-4.1012	-3.9823	-4.1784	0.2114
1, 2, 3-Butanetriol	20497	-3.9823	-3.5833	-4.2596	-4.1378	-3.8406	-3.9607	0.2636
3,5-Dimethyl-1,2,4-trithiolane	32033	-3.8039	-3.6558	-4.0430	-4.0930	-3.5953	-3.8382	0.2238
Formic acid	284	-2.8025	-2.7363	-2.8215	-3.0033	-5.2045	-3.3136	1.0617

Table S12. Main values from the HR1 per-residue decomposition of the free energy. The values are in kcal/mol. The cutoff point was -0.5 kcal/mol.

Residue	Alpha-tocotrienol	Amentoflavone	Isorhamnetin 3-O-rutinoside
Thr112		-0.557	
Phe116	-1.316		
Phe119	-1.193		
Ala151	-0.582		
Leu154	-2.204		
Leu157	-1.712	-2.233	
Trp158	-0.882	-2.946	
Ile160		-1.861	-1.997

Pro161		-2.511	-2.073
Gly164			-0.648
His167		-0.778	-4.335
Phe168		-0.627	-2.201
Gln170			-1.552
Gln171			-1.107
Trp189			-1.065
Phe190		-3.099	-1.101
Met193		-1.302	-0.584
Thr194	-1.018	-0.506	
Ile197	-2.855	-1.315	
Asn198	-0.838	-1.538	
Leu201	-1.142		
Pro202	-1.031		

Table S13. Main values from the NKR1 per-residue decomposition of the free energy. The values are in kcal/mol. The cutoff point was -0.5 kcal/mol.

Residue	Alpha-tocotrienol	Amentoflavone	Isorhamnetin 3-O-rutinoside	Neoxanthin	Aprepitant
Val26				-1.432	
Gln27				-0.629	
Asn89			-4.12		
Tyr92	-1.119	-0.892	-1.616	-0.732	
Ala93		-0.678	-0.879	-0.904	
Val94				-0.736	
Asn96				-0.673	
His108		-0.539	-1.257		-0.532
Asn109		-0.64	-2.32	-0.904	-0.94
Pro112	-1.147	-0.681	-0.713	-0.618	-0.555
Ile113	-1.349	-0.603	-1.108		-1.574
Val116	-0.63			-0.781	
Gln165		-0.854	-0.965		-2.474
Ser169			-0.733		
Val179	-0.719				
Cys180			-0.586		

Met181	-0.805		-2.462		
Ile182	-0.949	-1.531	-2.351	-0.58	-1.362
Trp184					-0.839
Tyr196					-0.835
His197			-0.592		-1.753
Val200					-0.733
Phe264	-0.644		-0.736		
His265	-1.134		-1.963		
Phe267	-0.533	-2.525	-0.848	-0.872	
Phe268	-2.013	-4.613	-4.755	-1.711	-2.097
Leu270		-0.708			
Leu279	-0.711				
Phe282				-0.831	
Ile283		-1.359	-0.674	-1.451	
Gln284	-0.577	-1.132		-1.783	
Tyr287	-0.715	-2.297	-1.027	-1.082	

Table S14. Main values from the CLR1 per-residue decomposition of the free energy. The values are in kcal/mol. The cutoff point was -0.5 kcal/mol.

Residue	Alpha-tocotrienol	Amentoflavone	Isorhamnetin 3-O-rutinoside	Neoxanthin	Zafirlukast
Arg97				-0.709	
Thr100		-0.965		-1.408	-0.858
Tyr101				-0.876	
Leu103		-0.639		-1.299	
Tyr104	-1.047	-0.849			
Val105	-0.52				
Tyr108	-1.123				-1.278
Phe112				-2.052	
Thr115				-1.196	
Ile147				-1.338	
Ile149			-0.627		
Phe150			-1.375	-1.694	
Val151	-1.038			-0.763	
Leu153			-1.515		
Thr154	-0.531		-1.535	-0.969	

Pro157			-0.656		-1.211
Phe158	-0.724	-1.576	-0.937	-1.836	-2.324
Ala161					-0.936
Pro163				-1.498	
Cys173				-1.238	
Phe174		-1.834		-1.383	-1.45
Glu175		-1.335		-1.224	
Pro176	-2.329	-3.254	-0.945	-1.755	-1.585
Pro177	-1.691	-2.728	-0.519	-0.506	-1.241
Val186	-1.301	-1.399	-0.762		-1.012
Leu189	-2.411	-1.955	-2.241	-0.753	-2.908
His190	-0.702	-0.613	-0.643		-1.134
Val192	-1.226		-1.196	-0.7	-0.505
Ser193	-1.301		-0.718	-0.579	-1.399
Val196	-0.866			-1.504	-0.569
Ile200				-0.736	
Pro201				-0.984	
Ile204				-0.548	
Arg253	-1.689	-1.855	-1.437		-2.179
His256	-0.98	-1.85	-0.846		
Leu257	-1.021	-1.317	-1.421		-0.682
Leu260	-0.731		-0.6		

Table S15. Main values from the CRTH2 per-residue decomposition of the free energy. The values are in kcal/mol. The cutoff point was -0.5 kcal/mol.

Residue	Alpha-tocotrienol	Amentoflavone	Isorhamnetin 3-O-rutinoside	Neoxanthin	Fevipiprant
Leu20			-0.527		
Phe87	-0.653	-2.806	-1.332	-0.644	-1.212
Thr88	-0.648	-0.648			
Phe90	-1.751	-2.45	-3.365	-2.04	-0.743
Leu91		-0.948	-1.045		
His95	-0.67			-1.77	
Trp97	-0.707	-0.867			

His107	-0.677	-0.785		-0.844	
Ser108	-0.708				-0.542
Phe111	-0.885			-1.028	-1.266
Phe112					-0.524
Arg170	-1.99	-1.238	-0.58	-1.093	-9.101
Met181	-1.334	-2.875	-1.926	-1.298	-0.664
Cys182	-0.969	-2.496	-1.135		
Tyr183	-0.596	-1.147	-1.592		-0.763
Tyr184		-0.574	-0.506		-1.439
Lys210		-1.071	-3.566	-1.703	-6.742
Tyr262				-0.832	-1.182
Glu269		-0.899			
Val282			-0.948		
Trp283		-0.806	-3.176		
Arg284			-0.802	-0.713	
Leu286	-1.036	-2.707	-2.237	-1.775	-3.188
Pro287	-2.752	-1.977	-3.249	-2.414	-1.168
Phe288	-0.728			-0.512	
Thr290	-1.727	-1.034		-1.603	-1.5
Phe294	-0.753				-0.586

Table S16. Main values from the BK2R per-residue decomposition of the free energy. The values are in kcal/mol. The cutoff point was -0.5 kcal/mol.

Residue	Alpha-tocotrienol	Amentoflavone	Isorhamnetin 3-O-rutinoside	Neoxanthin	JSM-10292
Gln49		-0.746			
Leu110		-0.627			
Trp113	-1.69	-3.607	-1.025	-1.758	-2.127
Thr116			-0.503		-0.622
Ile117	-0.632	-0.687			-0.895
Phe121	-0.688	-2.964			-2.388
Trp123			-0.54		
Asn134			-0.989		
Ile137	-1.309	-1.532	-0.741	-1.542	-1.013

Ser138	-0.83		-0.739	-1.193	-0.634
Leu141	-0.708			-1.031	
Met192	-0.685		-1.87	-0.848	-0.518
Arg196			-3.251		
Tyr201				-1.257	
Ala210		-0.884			
Cys211			-1.029		
Val212				-1.988	
Ile213	-0.703			-0.9	
Ser214				-0.609	
Tyr215				-1.218	
Leu218				-0.698	
Glu221			-1.74		
Thr224			-2.127		
Asn225			-0.69		
Leu228	-0.647		-0.831		-0.812
Phe286	-1.247	-0.753	-0.509		-0.781
Thr290	-0.505				
Arg297			-0.897		
Asp311		-1.85			
Thr314	-1.126	-1.33	-3.256		-0.722
Gln315	-2.369	-2.222	-1.772		-1.944
Ser318		-2.027		-1.097	-0.843
Phe319	-0.873				-0.502
Tyr322	-0.614			-1.006	

Table S17. UD´s Phytochemical Database.

Molecule	PubChem CID	Phytochemical Screening
**3,4-Dihydroxybenzoic acid**	**72**	**[Repajić, et al., 2021]**
**Aminobutyric acid**	**119**	**[Roschek Jr., et al., 2009]**
**p-Hydroxybenzyl alcohol**	**125**	**[Grauso, et al., 2020] [Majedi, et al., 2021]**
**p-Hydroxy-benzaldehyde**	**126**	**[Grauso, et al., 2020]**
**p-hydroxybenzoic acid**	**135**	**[Repajić, et al., 2021] [Grauso, et al., 2020]**
**adenine**	**190**	**[Roschek Jr., et al., 2009] [Ayers, et al.,2008]**

**Benzaldehyde**	**240**	**[Ilies, et al., 2012]**
**Formic acid**	**284**	**[Grauso, et al., 2020; Grauso et al., 2019]**
**Choline**	**305**	**[Grauso, et al., 2020] [Grauso, et al., 2019]**
**Citric acid**	**311**	**[Grauso, et al., 2019] [Grauso, et al., 2020]**
**Cumin aldehyde**	**326**	**[Gül, et al., 2012] [Grauso, et al., 2020]**
**4-Vinyl guaiacol**	**332**	**[Gül, et al., 2012]**
**Gallic acid**	**370**	**[Repajić, et al., 2021] [Grauso, et al., 2020]**
**n-Octanal**	**454**	**[Ilies, et al., 2012]**
**Creatinine**	**588**	**[Roschek Jr., et al., 2009]**
**Glutaric acid**	**743**	**[Roschek Jr., et al., 2009]**
**Histidinol**	**776**	**[Roschek Jr., et al., 2009]**
**Indole**	**798**	**[Roschek Jr., et al., 2009]**
**Naphthalene**	**931**	**[Gül, et al., 2012] [Grauso, et al., 2020]**
**Naringenin**	**932**	**[Repajić, et al., 2021]**
**Niacinamide**	**936**	**[Roschek Jr., et al., 2009]**
**Palmitic acid**	**985**	**[Grauso, et al., 2020]**
**Phthalic acid**	**1017**	**[Majedi, et al., 2021]**
**Pyrogallol**	**1057**	**[Roschek Jr., et al., 2009] [Ayers, et al.,2008]**
**Succinic acid**	**1110**	**[Grauso, et al., 2020] [Grauso, et al., 2019]**
**9-Hydroxy-10,12- octadecadienoic acid**	**1927**	**[Grauso, et al., 2020]**
**DL-methyl-m-tyrosine**	**2110**	**[Ayers, et al.,2008]**
**4-aminoantipyrine**	**2151**	**[Roschek Jr., et al., 2009]**
**Camphor**	**2537**	**[Ilies, et al., 2012]**
**Dibutyl phthalate**	**3026**	**[Majedi, et al., 2021]**
**Eugenol**	**3314**	**[Gül, et al., 2012] [Roschek Jr., et al., 2009]**
**Gentisic acid**	**3469**	**[Repajić, et al., 2021] [Grauso, et al., 2020]**
**Hydrocotarnine**	**3646**	**[Roschek Jr., et al., 2009]**
**Lauric acid**	**3893**	**[Grauso, et al., 2020]**
**1,4-benzoquinone**	**4650**	**[Roschek Jr., et al., 2009]**
**Resorcinol**	**5054**	**[Roschek Jr., et al., 2009]**
**Salicylic alcohol**	**5146**	**[Grauso, et al., 2020]**
**4-deoxypyridoxine**	**6094**	**[Roschek Jr., et al., 2009]**
**Leucine**	**6106**	**[Roschek Jr., et al., 2009]**
**Phenylalanine**	**6140**	**[Roschek Jr., et al., 2009]**
**Hexanal**	**6184**	**[Gül, et al., 2012] [Grauso, et al., 2020]**
**Mannitol**	**6251**	**[Roschek Jr., et al., 2009]**
**L-threonine**	**6288**	**[Roschek Jr., et al., 2009]**
**Bornyl acetate**	**6448**	**[Ilies, et al., 2012]**
**Quinic acid**	**6508**	**[Repajić, et al., 2021] [Garcia, et al., 2021] [Grauso, et al., 2020] [Grauso, et al., 2019]**
**Linalool**	**6549**	**[Gül, et al., 2012] [Grauso, et al., 2020]**

Vitamin B5	6613	[Roschek Jr., et al., 2009] [Ayers, et al.,2008]
Thymol	6989	[Ilies, et al., 2012] [Gül, et al., 2012]
1-Methyl naphthalene	7002	[Gül, et al., 2012]
Diphenyl	7095	[Roschek Jr., et al., 2009]
Methyl eugenol	7127	[Gül, et al., 2012]
Synephrine	7172	[Roschek Jr., et al., 2009] [Ayers, et al.,2008]
Carvylacetate	7335	[Roschek Jr., et al., 2009]
Acetophenone	7410	[Roschek Jr., et al., 2009]
Carvone	7439	[Gül, et al., 2012] [Grauso, et al., 2020]
p-Cymene	7463	[Gül, et al., 2012] [Grauso, et al., 2020]
p-Hydroxy-acetophenone	7469	[Grauso, et al., 2020]
2-Heptanone	8051	[Gül, et al., 2012] [Grauso, et al., 2020]
Heptanal	8130	[Gül, et al., 2012] [Grauso, et al., 2020]
1-Dodecanamine, N, N-dimethyl	8168	[Al-Tameme, et al., 2015] [Majedi, et al., 2021]
Decanal	8175	[Ilies, et al., 2012]
Methyl palmitate	8181	[Ilies, et al., 2012] [Grauso, et al., 2020]
Benzyl salicilate	8363	[Grauso, et al., 2020]
Vanillic acid	8468	[Grauso, et al., 2020]
m-Hydroxy-acetophenone	8487	[Grauso, et al., 2020]
Anthranilic acid methyl ester	8635	[Roschek Jr., et al., 2009]
Shikimic acid	8742	[Roschek Jr., et al., 2009] [Grauso, et al., 2020] [Grauso, et al., 2019]
Methyl chavicol	8815	[Gül, et al., 2012]
Nonanol	8914	[Roschek Jr., et al., 2009]
Catechin	9064	[Repajić, et al., 2021] [Grauso, et al., 2020]
β-Cyclocitral	9895	[Ilies, et al., 2012] [Gül, et al., 2012]
Osthole	10228	[Roschek Jr., et al., 2009] [Ayers, et al.,2008]
Indole-3-carboxaldehyde	10256	[Grauso, et al., 2020]
Benzofuran, 2,3,-dihydro	10329	[Al-Tameme, et al., 2015] [Majedi, et al., 2021]
Carvacrol	10364	[Ilies, et al., 2012] [Gül, et al., 2012] [Grauso, et al., 2020]
Tyrosol	10393	[Roschek Jr., et al., 2009] [Grauso, et al., 2020]
Hexahydrofarnesylacetone	10408	[Ilies, et al., 2012] [Gül, et al., 2012] [Grauso, et al., 2020]
Neophytadiene	10446	[Grauso, et al., 2020] [Majedi, et al., 2021]
Heptadecanoic acid	10465	[Grauso, et al., 2020]
Flavone	10680	[Majedi, et al., 2021]
Syringic acid	10742	[Repajić, et al., 2021] [Grauso, et al., 2020]
Myristic acid	11005	[Grauso, et al., 2020]
Levulinic acid	11579	[Roschek Jr., et al., 2009]
Dimethyl-L-tartrate	11851	[Roschek Jr., et al., 2009]
2,4,6-Trimethyl-5H-1,3,5-dithiazine	12518	[Ilies, et al., 2012]
Decan-2-one	12741	[Ilies, et al., 2012]

2-acetylpyrrole	14079	[Roschek Jr., et al., 2009]
Homovanillyl alcohol	16928	[Grauso, et al., 2020]
α- Terpineol	17100	[Gül, et al., 2012] [Grauso, et al., 2020]
Methylcoumarin	17130	[Roschek Jr., et al., 2009]
6-aminochrysene	17534	[Roschek Jr., et al., 2009]
2-Pentylfuran	19602	[Ilies, et al., 2012] [Gül, et al., 2012] [Grauso, et al., 2020]
α-Copaene	19725	[Grauso, et al., 2020]
1, 2, 3-Butanetriol	20497	[Al-Tameme, et al., 2015] [Majedi, et al., 2021]
Isopropyl dodecanoate	25068	[Ilies, et al., 2012]
Rhamnose	25310	[Grauso, et al., 2020]
Anisaldehyde	31244	[Roschek Jr., et al., 2009]
Nonanal	31289	[Ilies, et al., 2012] [Gül, et al., 2012] [Grauso, et al., 2020]
3,5-Dimethyl-1,2,4-trithiolane	32033	[Ilies, et al., 2012]
Adenosine	60961	[Roschek Jr., et al., 2009]
Safranal	61041	[Ilies, et al., 2012]
β-Homocyclocitral	61124	[Ilies, et al., 2012]
6-benzylaminopurine	62389	[Roschek Jr., et al., 2009]
β-Bourbonene	62566	[Grauso, et al., 2020] [Gül, et al., 2012]
Borneol	64685	[Ilies, et al., 2012] [Gül, et al., 2012]
Apoatropine	64695	[Ilies, et al., 2012]
Ursolic acid	64945	[Majedi, et al., 2021]
Epigallocatechin gallate	65064	[Repajić, et al., 2021]
Secoisolariciresinol	65373	[Grauso, et al., 2020]
Solanidine	65727	[Roschek Jr., et al., 2009]
4-methyl-7-ethoxycoumarin	66595	[Roschek Jr., et al., 2009]
Harmol	68094	[Roschek Jr., et al., 2009]
4-Acetyl-2-methylphenol	70135	[Grauso, et al., 2020]
6-azacytosine	70265	[Roschek Jr., et al., 2009]
Epicatechin	72276	[Repajić, et al., 2021] [Grauso, et al., 2020]
Isopilosine	72312	[Roschek Jr., et al., 2009]
9-amino-camptothecin	72402	[Roschek Jr., et al., 2009]
Carvacryl acetate	80792	[Roschek Jr., et al., 2009]
1,2-O-isopropylidene-D-glucoside	87704	[Roschek Jr., et al., 2009]
1,2-Benzenedicarboxylic acid	90531	[Majedi, et al., 2021]
Hecogenin	91453	[Roschek Jr., et al., 2009]
L-2-aminoadipic acid	92136	[Roschek Jr., et al., 2009]
ar-Curcumene	92139	[Gül, et al., 2012]
γ-Cadinene	92313	[Ilies, et al., 2012] [Gül, et al., 2012] [Grauso, et al., 2020]
Podocarpic acid	93017	[Roschek Jr., et al., 2009]
Arabitol	94154	[Roschek Jr., et al., 2009]

Flavan	94156	[Roschek Jr., et al., 2009]
2,6-Dimethoxy-hydroquinone	96038	[Grauso, et al., 2020]
DL-a aminopimelic acid	101122	[Roschek Jr., et al., 2009]
Epicatechin gallate	107905	[Repajić, et al., 2021]
Catechin hydrate	107957	[Grauso, et al., 2020]
α- Terpinyl acetate	111037	[Gül, et al., 2012]
3-hydroxy-2,3 dihydromaltol	119838	[Roschek Jr., et al., 2009]
Proline	145742	[Roschek Jr., et al., 2009]
Anthocyanins	145858	[Majedi, et al., 2021]
Isolariciresinol	160521	[Grauso, et al., 2020]
7α-Hydroxy sitosterol	161816	[Grauso, et al., 2020]
n-acetyl-L-glutamine	182230	[Roschek Jr., et al., 2009]
Beta-Sitosterol	222284	[Majedi, et al., 2021]
4-methylmethoxycoumarin	223821	[Roschek Jr., et al., 2009]
3-Octanone	246728	[Ilies, et al., 2012]
Deoxyharringtonine	285342	[Roschek Jr., et al., 2009]
Convolvamine	420422	[Roschek Jr., et al., 2009]
Glucosamine	439213	[Roschek Jr., et al., 2009]
Carnosine	439224	[Roschek Jr., et al., 2009]
Arbutine	440936	[Grauso, et al., 2020]
δ-Cadinene	441005	[Ilies, et al., 2012] [Gül, et al., 2012] [Grauso, et al., 2020]
Lotaustralin	441467	[Roschek Jr., et al., 2009]
Carnosol	442009	[Roschek Jr., et al., 2009]
Pukateine	442340	[Roschek Jr., et al., 2009]
α-Copaene	442355	[Gül, et al., 2012]
β-Selinene	442393	[Ilies, et al., 2012]
Cinnamic acid	444539	[Repajić, et al., 2021]
Arachidonic acid	444899	[Grauso, et al., 2020]
Farnesol	445070	[Ilies, et al., 2012]
Palmitoleic acid	445638	[Grauso, et al., 2020]
Ferulic acid	445858	[Repajić, et al., 2021] [Garcia, et al., 2021] [Roschek Jr., et al., 2009] [Grauso, et al., 2020] [Majedi, et al., 2021]
Violaxanthin	448438	[Grauso, et al., 2020]
γ-sitosterol	457801	[Grauso, et al., 2020]
Silane, triethyl(2phenylethoxy)	518978	[Majedi, et al., 2021]
β - Sesquiphellandrene	519764	[Gül, et al., 2012]
α-Longipinene	520957	[Ilies, et al., 2012]
5,6-Dihydro-4-pentyl- 2,6-dimethyl-4H-1,3,5-dithiazine	528360	[Ilies, et al., 2012]
2(3H)-Naphthalenone, 4, 4a,5,6,7,8-hexahydro-1-methoxy	534313	[Al-Tameme, et al., 2015] [Majedi, et al., 2021]
4-(4-Hydroxy-2,6,6-trimethyl1-cyclohexen-1-yl)-3-buten2-one	538953	[Grauso, et al., 2020]

2H-Indeno[1,2-b]furan-2-one, 3,3a,4,5,6,7,8,8b-octahydro-8,8-dimethyl	605626	[Majedi, et al., 2021]
Silane, triethyl(2-phenylethoxy)	610043	[Al-Tameme, et al., 2015]
p-coumaric acid	637542	[Repajić, et al., 2021] [Pinelli, et al., 2008] [Grauso, et al., 2020]
(E)-Anethole	637563	[Gül, et al., 2012] [Grauso, et al., 2020]
Sinapic acid	637775	[Repajić, et al., 2021] [Grauso, et al., 2020]
β-Ionone	638014	[Ilies, et al., 2012] [Gül, et al., 2012] [Grauso, et al., 2020]
Piperine	638024	[Roschek Jr., et al., 2009]
Isopropyl-β-D-thio-galacto-pyranoside	656894	[Roschek Jr., et al., 2009]
Caffeic acid	689043	[Garcia, et al., 2021] [Repajić, et al., 2021] [Grauso, et al., 2020]
Coniferol	1549095	[Grauso, et al., 2020] [Roschek Jr., et al., 2009]
Geranyl acetone	1549778	[Ilies, et al., 2012]
Farnesylacetone	1711945	[Ilies, et al., 2012] [Gül, et al., 2012]
(E)-Geranyl acetone	1713001	[Gül, et al., 2012] [Grauso, et al., 2020]
Caryophyllene oxide	1742210	[Gül, et al., 2012]
Chlorogenic acid	1794427	[Repajić, et al., 2021] [Pinelli, et al., 2008] [Grauso, et al., 2020] [Majedi, et al., 2021] [Grauso, et al., 2019]
Levoglucosan	2724705	[Roschek Jr., et al., 2009]
Galactal	2734735	[Roschek Jr., et al., 2009]
Cinnamide	5273472	[Roschek Jr., et al., 2009]
Quercetin	5280343	[Repajić, et al., 2021] [Grauso, et al., 2020] [Majedi, et al., 2021]
Phytol	5280435	[Ilies, et al., 2012] [Gül, et al., 2012] [Grauso, et al., 2020]
Apigenin	5280443	[Repajić, et al., 2021]
Luteolin	5280445	[Repajić, et al., 2021] [Grauso, et al., 2019]
Quercitrin	5280459	[Grauso, et al., 2020]
Scopoletin	5280460	[Repajić, et al., 2021] [Grauso, et al., 2020] [Majedi, et al., 2021]
β-carotene	5280489	[Grauso, et al., 2020]
Sinapyl alcohol	5280507	[Grauso, et al., 2020]
Neochlorogenic acid	5280633	[Grauso, et al., 2020] [Grauso, et al., 2019]
4-(3-Hydroxy-1-butyn-1-yl)- 3,5,5-trimethyl-2- cyclohexen-1-ol	5280654	[Grauso, et al., 2020]
Crysoeriol	5280666	[Grauso, et al., 2020]
Cholecalciferol	5280795	[Roschek Jr., et al., 2009]
Quercetin-3-glucoside	5280804	[Repajić, et al., 2021] [Pinelli, et al., 2008] [Garcia, et al., 2021]
Rutin	5280805	[Repajić, et al., 2021] [Pinelli, et al., 2008] [Garcia, et al., 2021] [Majedi, et al., 2021]
Kaempferol	5280863	[Repajić, et al., 2021] [Grauso, et al., 2020]
Linolenic acid	5280934	[Roschek Jr., et al., 2009] [Grauso, et al., 2020]
Harmine	5280953	[Roschek Jr., et al., 2009]
Genistein	5280961	[Repajić, et al., 2021]
Myristoleic acid	5281119	[Grauso, et al., 2020]

Lutein	5281243	[Grauso, et al., 2020]
Esculetin	5281416	[Repajić, et al., 2021] [Grauso, et al., 2020]
Umbelliferone	5281426	[Repajić, et al., 2021]
β-Caryophyllene	5281515	[Ilies, et al., 2012] [Grauso, et al., 2020] [Gül, et al., 2012]
α-Humulene	5281520	[Ilies, et al., 2012]
Kavain	5281565	[Roschek Jr., et al., 2009]
Amentoflavone	5281600	[Grauso, et al., 2020]
Isorhamnetin	5281654	[Repajić, et al., 2021] [Grauso, et al., 2020]
Myricetin	5281672	[Repajić, et al., 2021]
Pterostilbene	5281727	[Roschek Jr., et al., 2009]
Integerrimine	5281733	[Roschek Jr., et al., 2009]
Kaempferol-3-O-glucoside	5282102	[Grauso, et al., 2020]
α-Ionone	5282108	[Ilies, et al., 2012]
Cinnamyl acetate	5282110	[Roschek Jr., et al., 2009]
Neoxanthin	5282217	[Grauso, et al., 2020]
Alpha-tocotrienol	5282347	[Roschek Jr., et al., 2009]
Homatropine	5282593	[Roschek Jr., et al., 2009]
Dodecendioic acid	5283028	[Grauso, et al., 2020]
cis-10-Heptadecenoic acid	5312435	[Grauso, et al., 2020]
Eleutheroside B	5316860	[Roschek Jr., et al., 2009]
10-gingerdione	5317591	[Roschek Jr., et al., 2009]
Isorhamnetin-3-O-glucoside	5318645	[Grauso, et al., 2020]
Kaempferol-3-rutinoside	5318767	[Repajić, et al., 2021] [Pinelli, et al., 2008] [Grauso, et al., 2020]
Quercetin dihexoside	5320835	[Grauso, et al., 2020]
3,4-dimethoxychalcone	5354494	[Roschek Jr., et al., 2009]
2, 6,-Nonadienal, 3, 7-dimethyl	5364526	[Al-Tameme, et al., 2015] [Majedi, et al., 2021]
3-Hydroxy-damascone	5366075	[Grauso, et al., 2020]
2-(1-Pentenyl)furan	5369956	[Ilies, et al., 2012]
3-Oxo-a-ionol	5370052	[Grauso, et al., 2020]
Abscisic acid	5375199	[Roschek Jr., et al., 2009]
2-deoxy-D-ribose	5460005	[Roschek Jr., et al., 2009]
Isorhamnetin 3-O-rutinoside	5481663	[Pinelli, et al., 2008] [Grauso, et al., 2020]
Kaempferol rhamnoside	5835713	[Repajić, et al., 2021]
Caffeoylmalic acid	6124299	[Grauso, et al., 2020] [Majedi, et al., 2021] [Pinelli, et al., 2008] [Garcia, et al., 2021]
1,2-Diguaiacyl-1,3- propanediol	6426042	[Grauso, et al., 2020]
Calamenene	6429077	[Gül, et al., 2012]
3,4-Dimethyl-5-pentylidene-2(5H)-furanone	6433214	[Gül, et al., 2012]
Oxime- methoxy-phenyl	9602988	[Al-Tameme, et al., 2015] [Majedi, et al., 2021]
4-shogaol	9794897	[Roschek Jr., et al., 2009] [Ayers, et al.,2008]
Estra-1,3,5(10)-trien-17B-ol	9811784	[Majedi, et al., 2021]

Caffeoyl tartaric acid	9857913	[Grauso, et al., 2020]
(+)-Neo-olivil	9976812	[Grauso, et al., 2020]
β-Bisabolene	10104370	[Ilies, et al., 2012] [Gül, et al., 2012] [Grauso, et al., 2020]
Caffeoylquinic acid	10155076	[Pinelli, et al., 2008]
O-Feruloyl quinic acid	10177048	[Grauso, et al., 2020]
Dihydrokavain	10220256	[Roschek Jr., et al., 2009]
1-Hydroxy-1-(4- hydroxyphenyl)-2-propanone	10261435	[Grauso, et al., 2020]
1,6-dioxaspiro[4.4]non-3-ene	10374471	[Roschek Jr., et al., 2009]
Phosphatidylcholine	10425706	[Roschek Jr., et al., 2009]
α-Selinene	10856614	[Ilies, et al., 2012]
Tachioside	11962143	[Grauso, et al., 2020]
Phytosterols	12303662	[Majedi, et al., 2021]
7β-Hydroxy-sitosterol	12309569	[Grauso, et al., 2020]
Dicaffeoylquinic acid	12358846	[Grauso, et al., 2020]
3,4-Dimethyl-5-pentylfuran-2(5H)-one	13192443	[Gül, et al., 2012]
β - Vetivenene	14475467	[Ilies, et al., 2012]
Kaempferol pentoside	14749097	[Repajić, et al., 2021]
Isotachioside	15098566	[Grauso, et al., 2020]
Bioallethrin	15558638	[Roschek Jr., et al., 2009]
Quercetin rhamnoside	15939939	[Repajić, et al., 2021]
α- Copaene-8-ol	25086830	[Ilies, et al., 2012]
Quercetin-3-O-glucoside	25203368	[Grauso, et al., 2020]
Peonidin 3-O-rutinoside	44256842	[Pinelli, et al., 2008] [Grauso, et al., 2020]
Apigenin-7-O-glucoside	44257792	[Grauso, et al., 2020] [Grauso, et al., 2019]
[1,1-Bicyclopropyl-2-octanoic acid 2hexyl-methyl ester	50930793	[Al-Tameme, et al., 2015] [Majedi, et al., 2021]
4-O-Caffeoylquinic acid	58427569	[Grauso, et al., 2020]
Hydroxycinnamaldehyde	71407359	[Roschek Jr., et al., 2009]
Feruloyl malate	71694479	[Grauso, et al., 2020]
Incensole oxide acetate	73755086	[Roschek Jr., et al., 2009]
Incensole oxide	90470329	[Roschek Jr., et al., 2009]
Sitosterol-β-D-glucoside	91884650	[Grauso, et al., 2020]
p-Coumaroylmalic acid	129720114	[Grauso, et al., 2020]
Caffeoyl feruloyl tartaric acid	129724266	[Grauso, et al., 2020]
8-dehydrogingerdione	131752598	[Roschek Jr., et al., 2009]
Isorhamnetin rutinoside	133562525	[Repajić, et al., 2021] [Garcia, et al., 2021]

Table S18. Structure used and detailed information about the system characteristics.

Receptor	PDB ID	Co-crystallized ligand	Resolution	Reference
Histamine receptor 1 (HR1)	7DFL	Histamine	3.30 Å	[Xia, et al., 2021]
Neurokinin 1 receptor (NK1R)	6J20	Aprepitant	2.70 Å	[Chen, et al., 2019]
Cysteinyl leukotriene receptor 1 (CLR1)	6rz5	Zafirlukast	2.53 Å	[Luginina, et a., 2019]
Chemoattractant receptor–homologous molecule expressed on type 2 helper T cells (CRTH2)	7m8w	15mPGD2	2.61 Å	[Liu, et al., 2021]
Bradykinin receptor type 2 (BK2R)	7F6H	Bradykinin	2.90 Å	[Shen, et al., 2022]

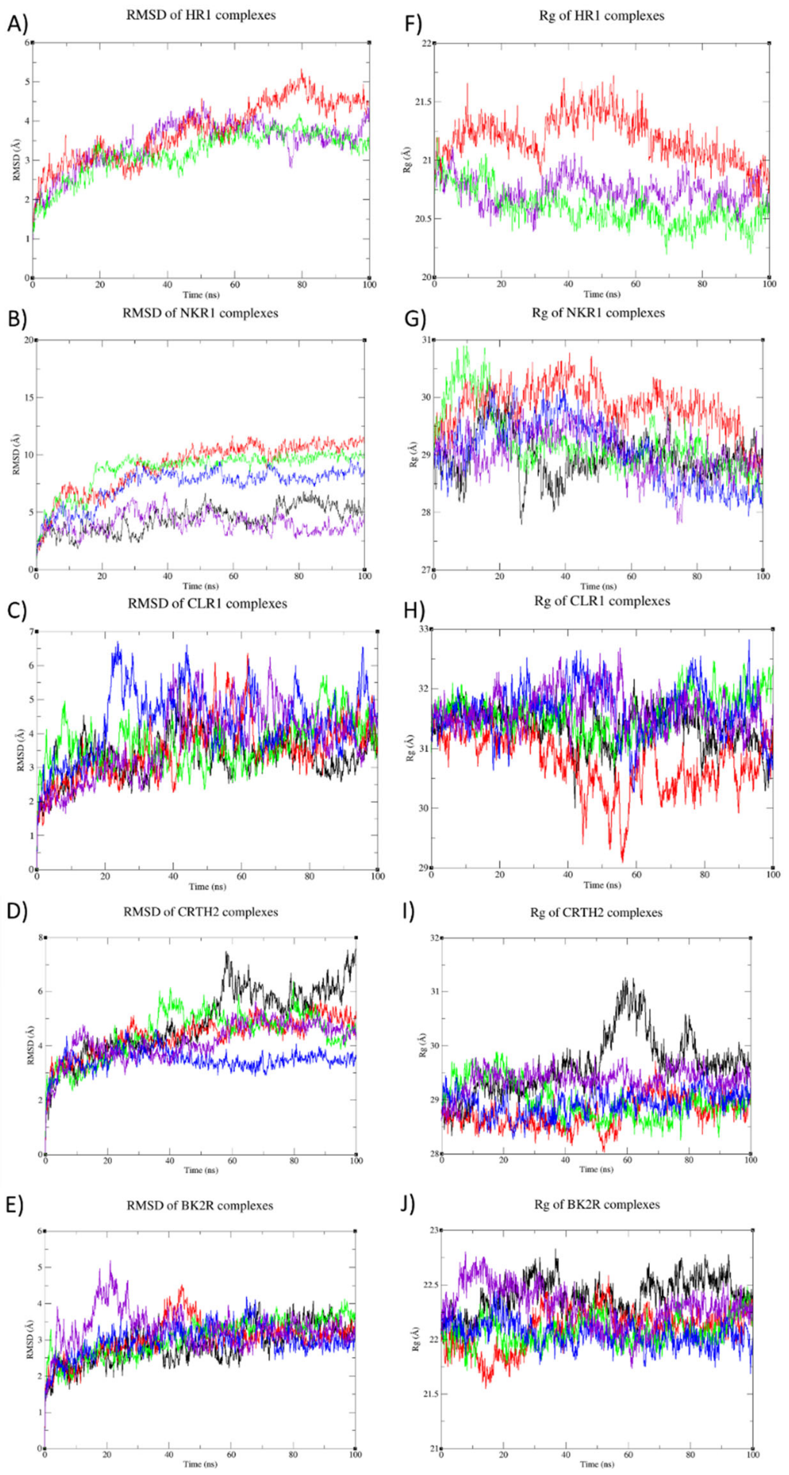

Figure S1. RMSD of A) HR1, B) NKR1, C) CLR1, D) CRTH2, E) BK2R. Rg of F) HR1, G) NKR1, H) CLR1, I) CRTH2, J) BK2R. Color code: Black: Known inhibitor, Red: Alpha-tocotrienol, Green: Amentoflavone, Purple: Isorhamnetin-3-O-rutinoside, and Blue: neoxanthin.

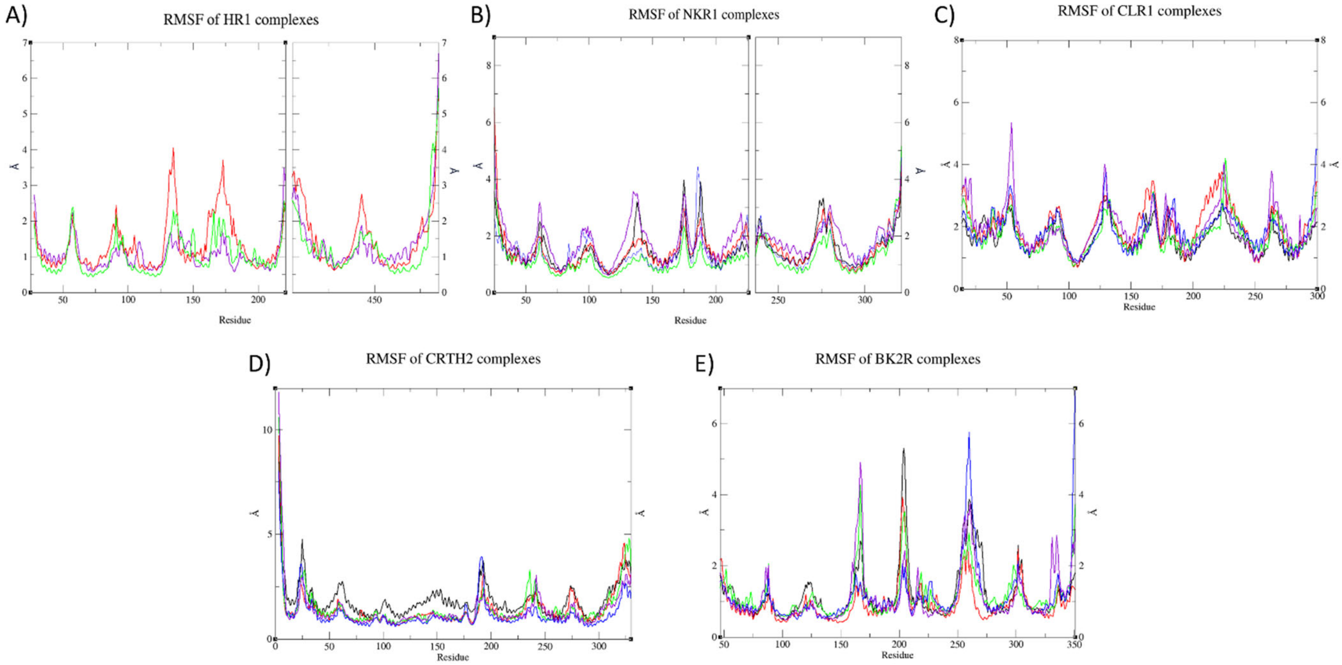

Figure S2. RMSF of A) HR1, B) NKR1, C) CLR1, D) CRTH2, E) BK2R. Color code: Black: Known inhibitor, Red: Alpha-tocotrienol, Green: Amentoflavone, Purple: Isorhamnetin-3-O-rutinoside, and Blue: neoxanthin.

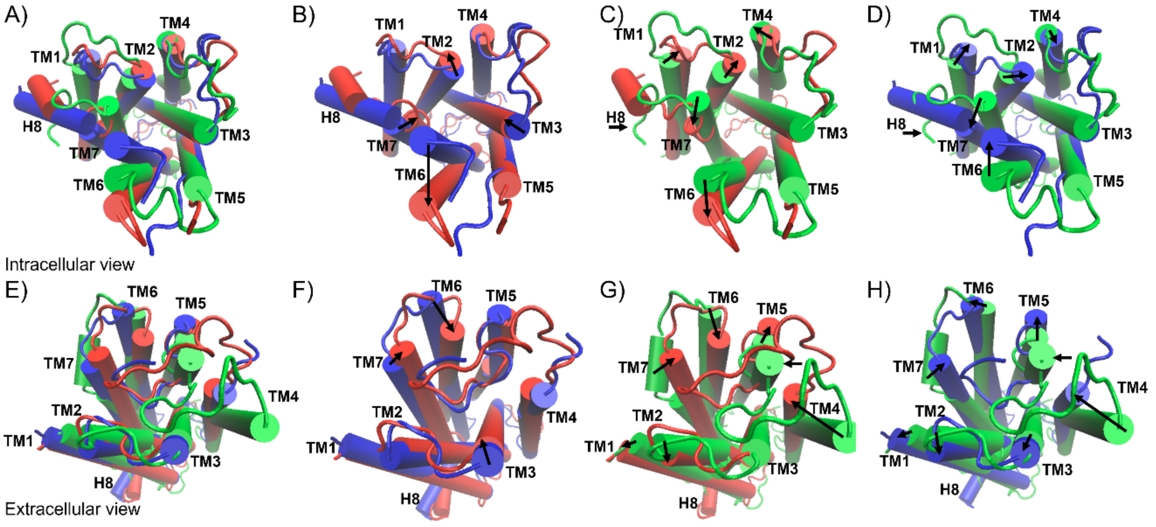

Figure S3. Structural comparison between HR1-alpha-tocotrienol complex (Green), HR1-Histamine (Red) and HR1-Doxepin (Blue). Intracellular view of A) the 3 structures, B) HR1-Histamine and HR1-

Doxepin, C) HR1-Histamine and HR1-alpha-tocotrienol, D) HR1-alpha-tocotrienol and HR1-Doxepin. Extracellular view of E) the 3 structures, F) HR1-Histamine and HR1-Doxepin, G) HR1-Histamine and HR1-alpha-tocotrienol, H) HR1-alpha-tocotrienol and HR1-Doxepin.

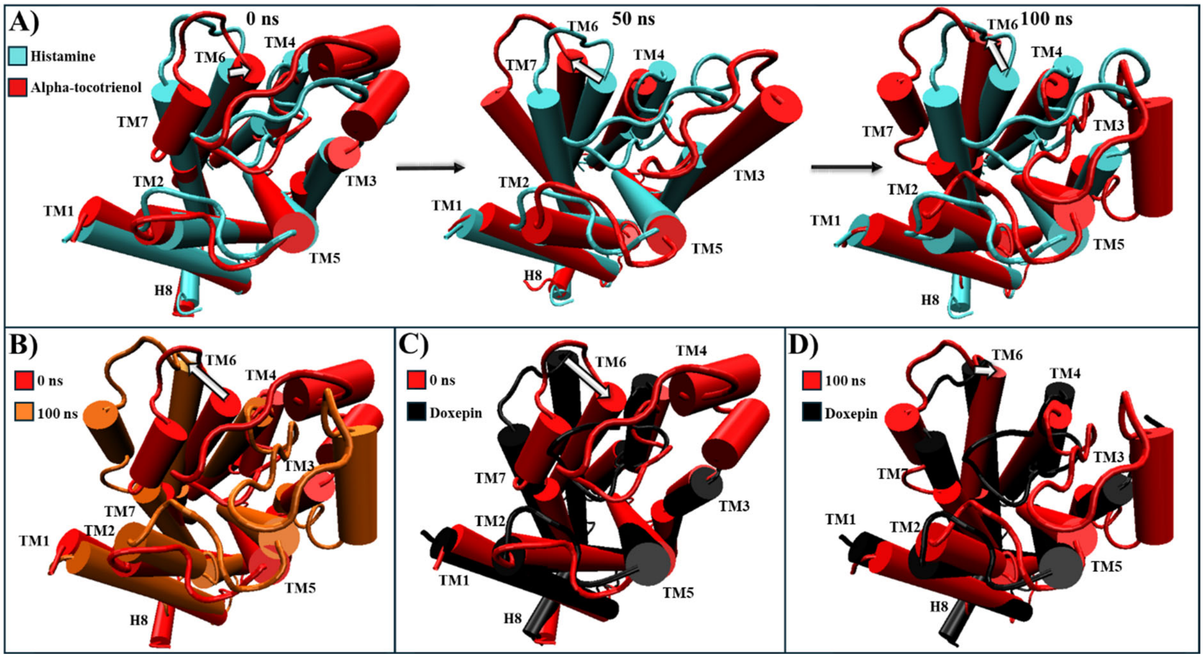

Figure S4. Structural comparison at the extracellular view of A) alpha-tocotrienol complex (red) within 0, 50 and 100 ns and Histamine experimental structure (cyan), B) alpha-tocotrienol complex at 0 ns (red), and 100 ns (orange), C) alpha-tocotrienol complex at 0 ns (red), and Doxepin experimental structure (black), D) alpha-tocotrienol complex at 100 ns (red), and Doxepin experimental structure (black).

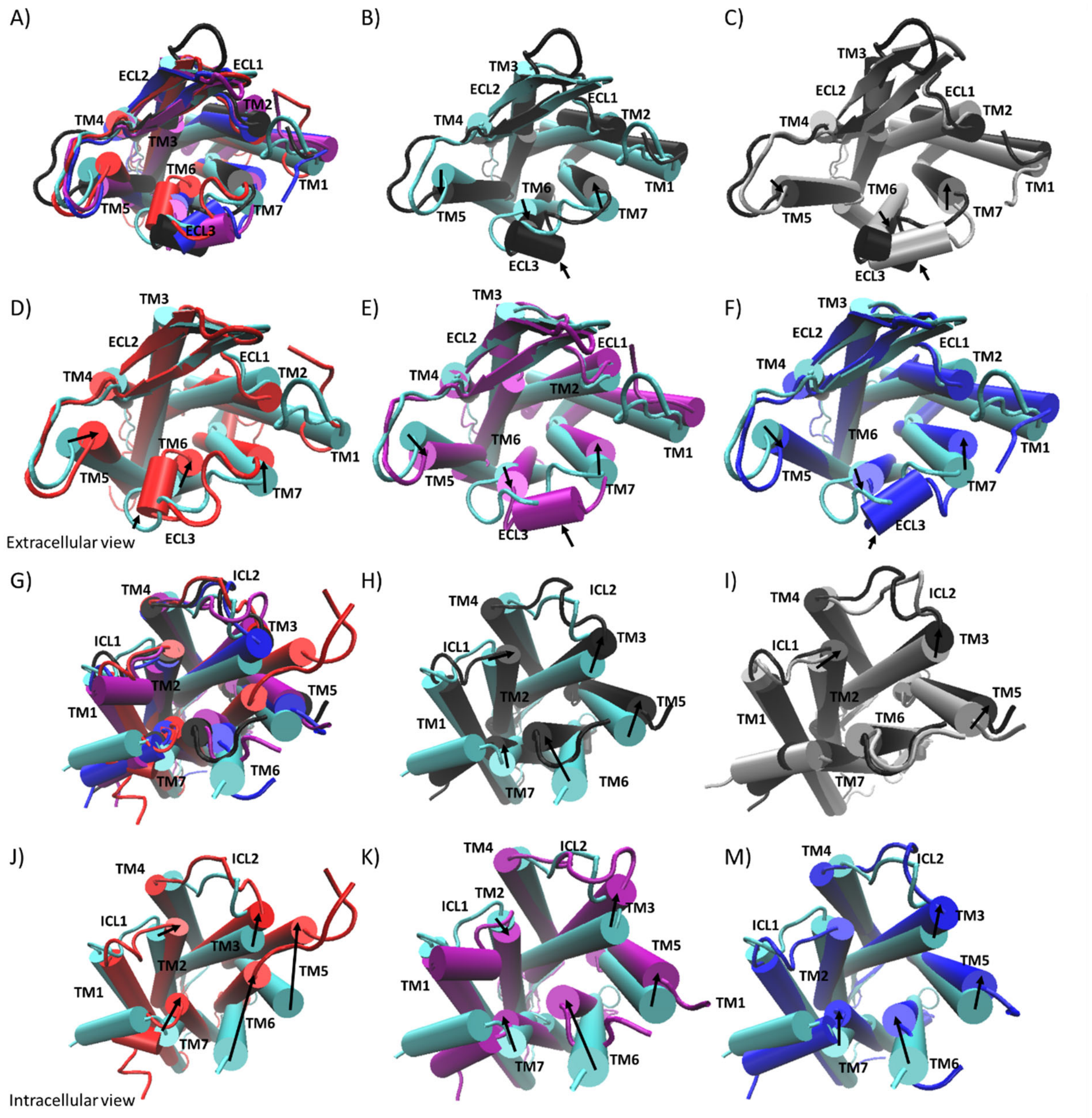

Figure S5. Structural comparison between structures. Extracellular view of A) all structures, B) NKR1-Substance P (turquoise) and MD NKR1-aprepitant (black), C) MD NKR1-aprepitant and experimental NKR1-aprepitant (silver), and NKR1-Substance P with D) NKR1-alpha-tocotrienol (red), E) NKR1-isorhamnetin 3-O-rutinoside (purple), and F) NKR1-neoxanthin (blue). Intracellular view of G) all structures, H) NKR1-Substance P and MD NKR1-aprepitant, I) MD NKR1-aprepitant and experimental NKR1-aprepitant, and NKR1-Substance P with J) NKR1-alpha-tocotrienol, K) NKR1-isorhamnetin 3-O-rutinoside, and M) NKR1-neoxanthin.

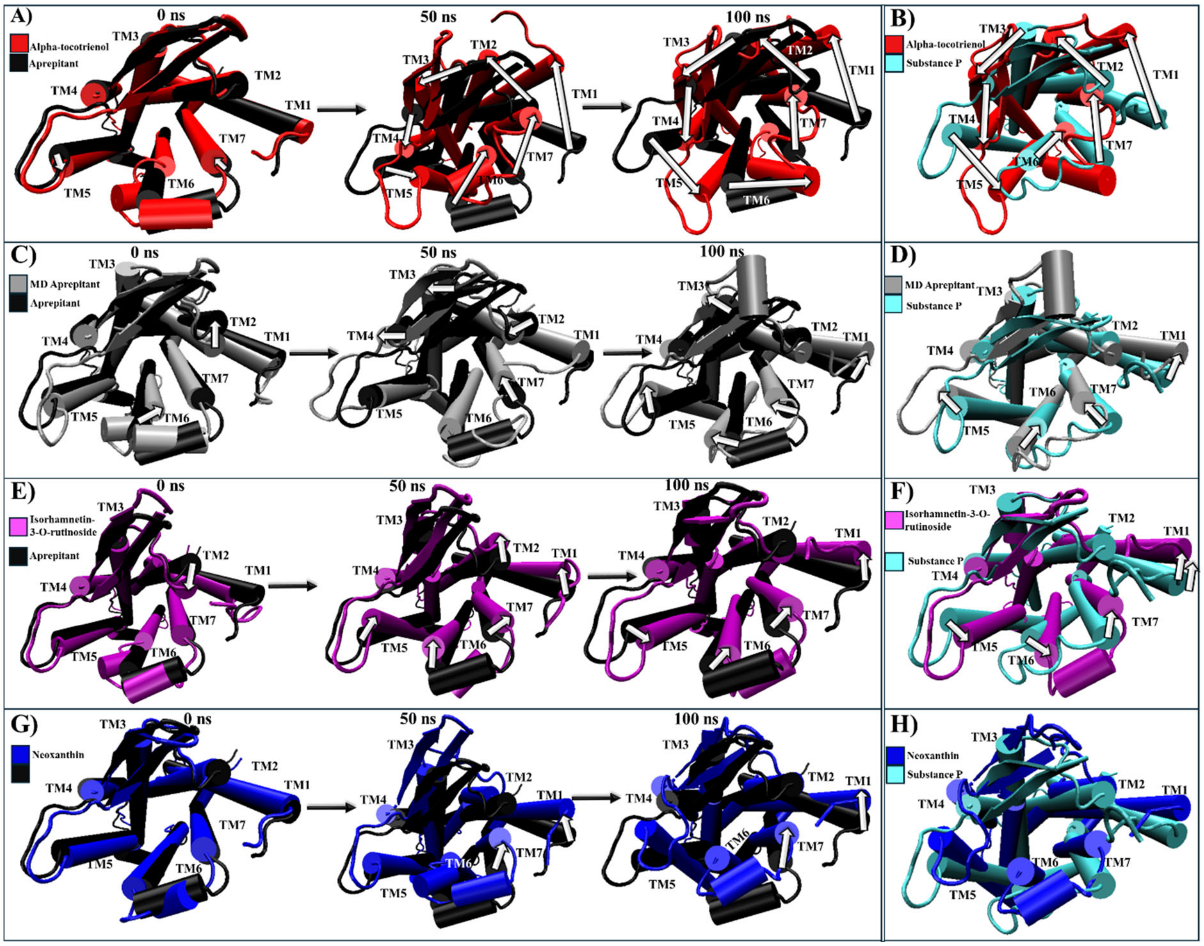

Figure S6. NKR1 structural comparison at the extracellular view of A) alpha-tocotrienol complex (red) within 0, 50 and 100 ns and experimental structure of NKR1/aprepitant (black), B) alpha-tocotrienol complex and the experimental structure of NKR1/ Substance P (Cyan), C) MD aprepitant complex (red) within 0, 50 and 100 ns and experimental structure of NKR1/aprepitant (black), D) MD aprepitant complex and the experimental structure of NKR1/ Substance P (Cyan), E) Isorhamnetin-3-O-rutinoside complex (red) within 0, 50 and 100 ns and experimental structure of NKR1/aprepitant (black), F) Isorhamnetin-3-O-rutinoside complex and the experimental structure of NKR1/ Substance P (Cyan), G) Neoxanthin complex (red) within 0, 50 and 100 ns and experimental structure of NKR1/aprepitant (black), H) Neoxanthin complex and the experimental structure of NKR1/ Substance P (Cyan).

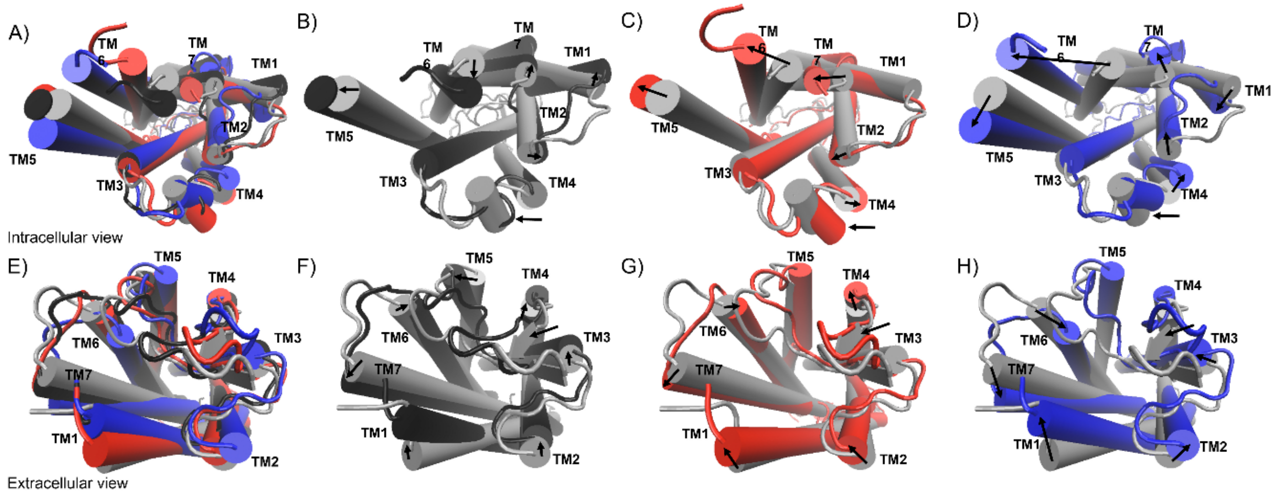

Figure S7. Structural comparison between structures. Intracellular view of experimental CLR1-zafirlukast and A) all structures, B) MD CLR1-zafirlukast, C) CLR1-alpha-tocotrienol (red), D) CLR1-neoxanthin (blue). Extracellular view of CLR1-zafirlukast and E) all structures, F) MD CLR1-zafirlukast, G) CLR1-alpha-tocotrienol (red), H) CLR1-neoxanthin (blue).

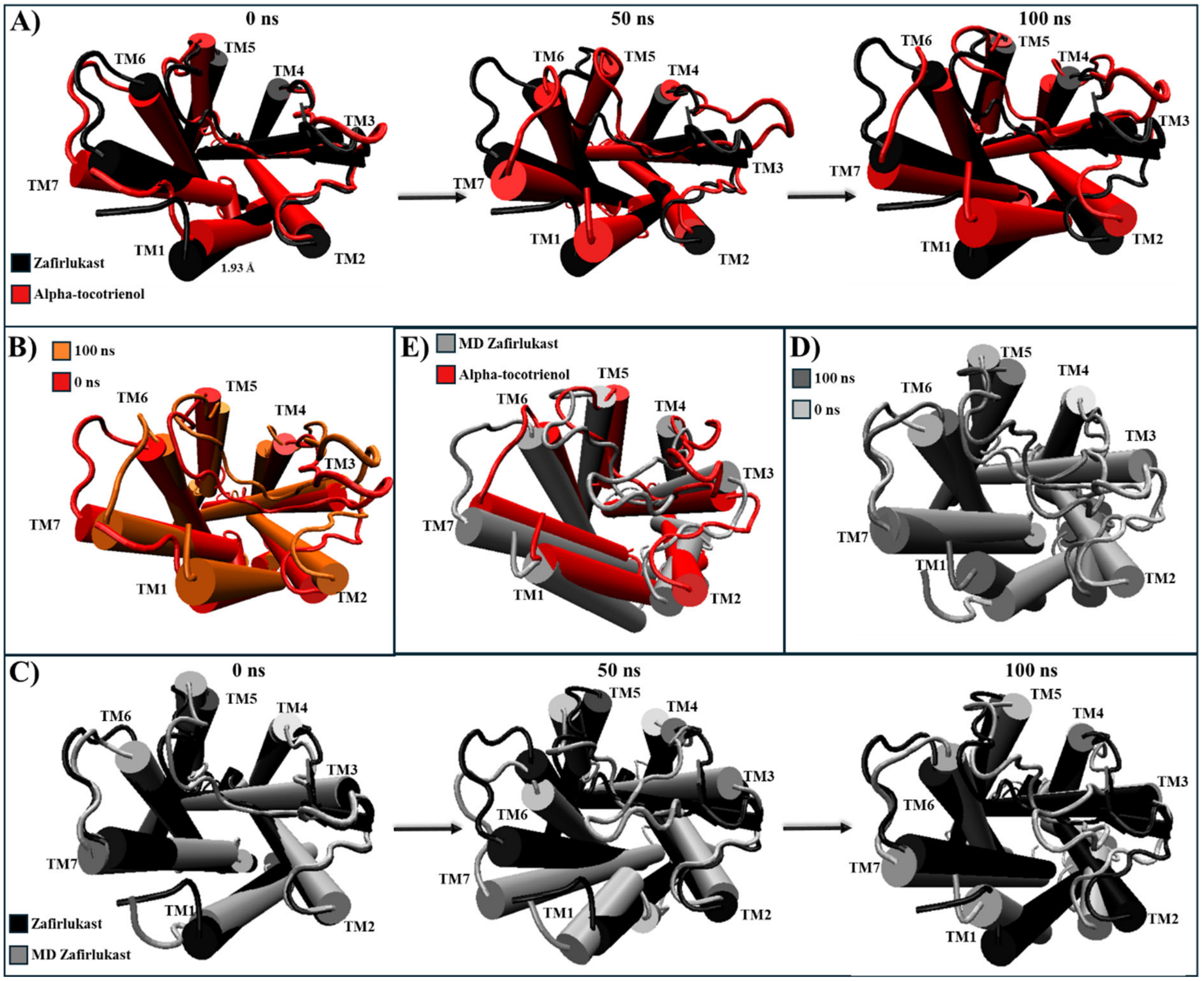

Figure S8. CLR1 structural comparison at the extracellular view of A) alpha-tocotrienol complex (red) within 0, 50 and 100 ns and experimental structure of CLR1/zafirlukast (black), B) alpha-tocotrienol complex at 0 ns (red) and 100 ns (orange), C) MD zafirlukast complex (silver) within 0, 50 and 100 ns and experimental structure of CLR1/zafirlukast (black), D) zafirlukast complex at 0 ns (silver) and 100 ns (grey), E) alpha-tocotrienol complex (red) and MD zafirlukast at 100 ns (silver).

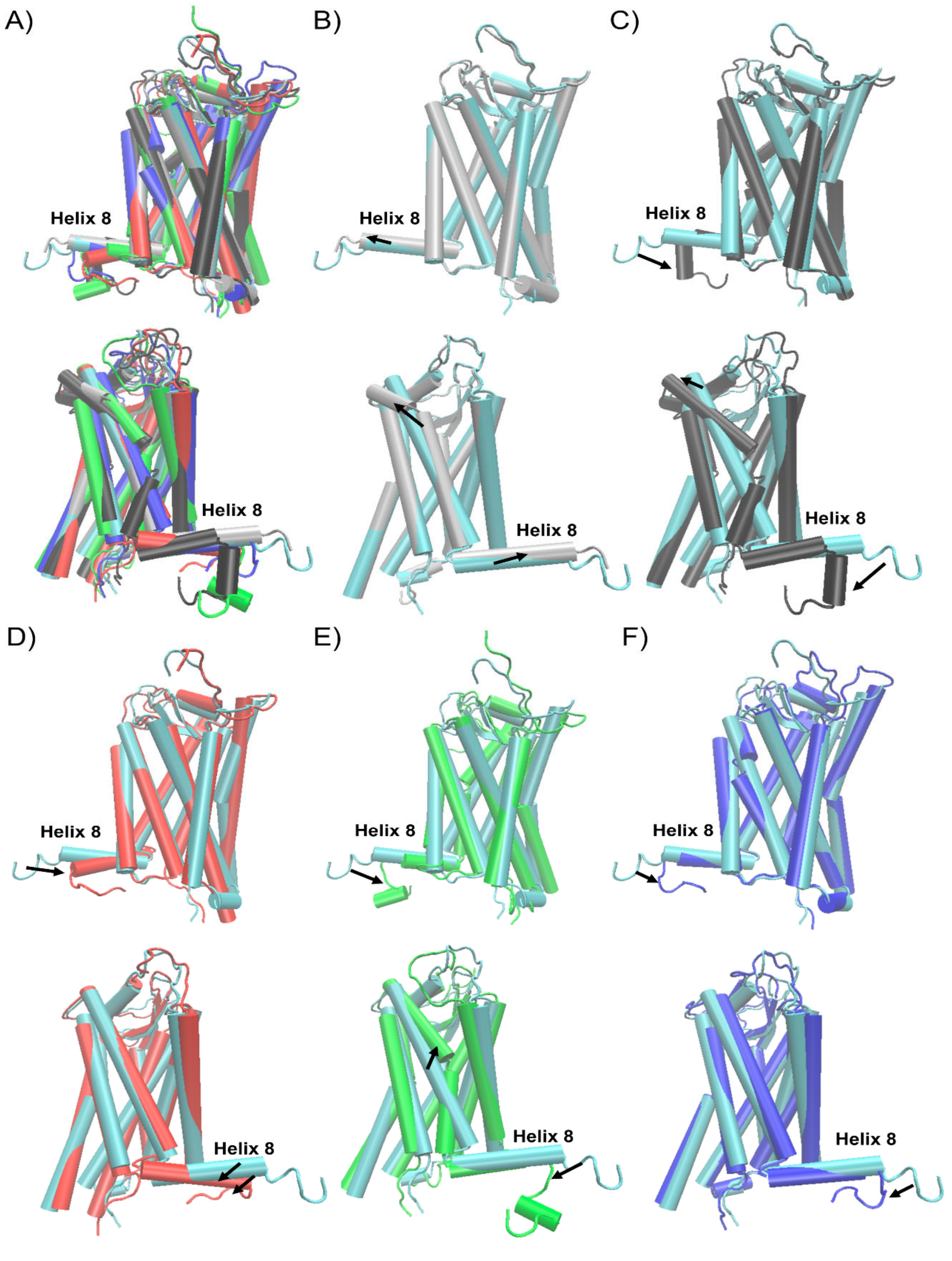

Figure S9. Structural comparison between structures. Lateral view of experimental CRTH2- 15R-methyl-PGD2 and A) all structures, B) experimental structure of CRTH2-fevipiprant (silver), C) MD CRTH2-fevipiprant (black), D) CRTH2-alpha-tocotrienol (red), E) CRTH2-amentoflavone (green), and F) CRTH2-neoxanthin (blue).

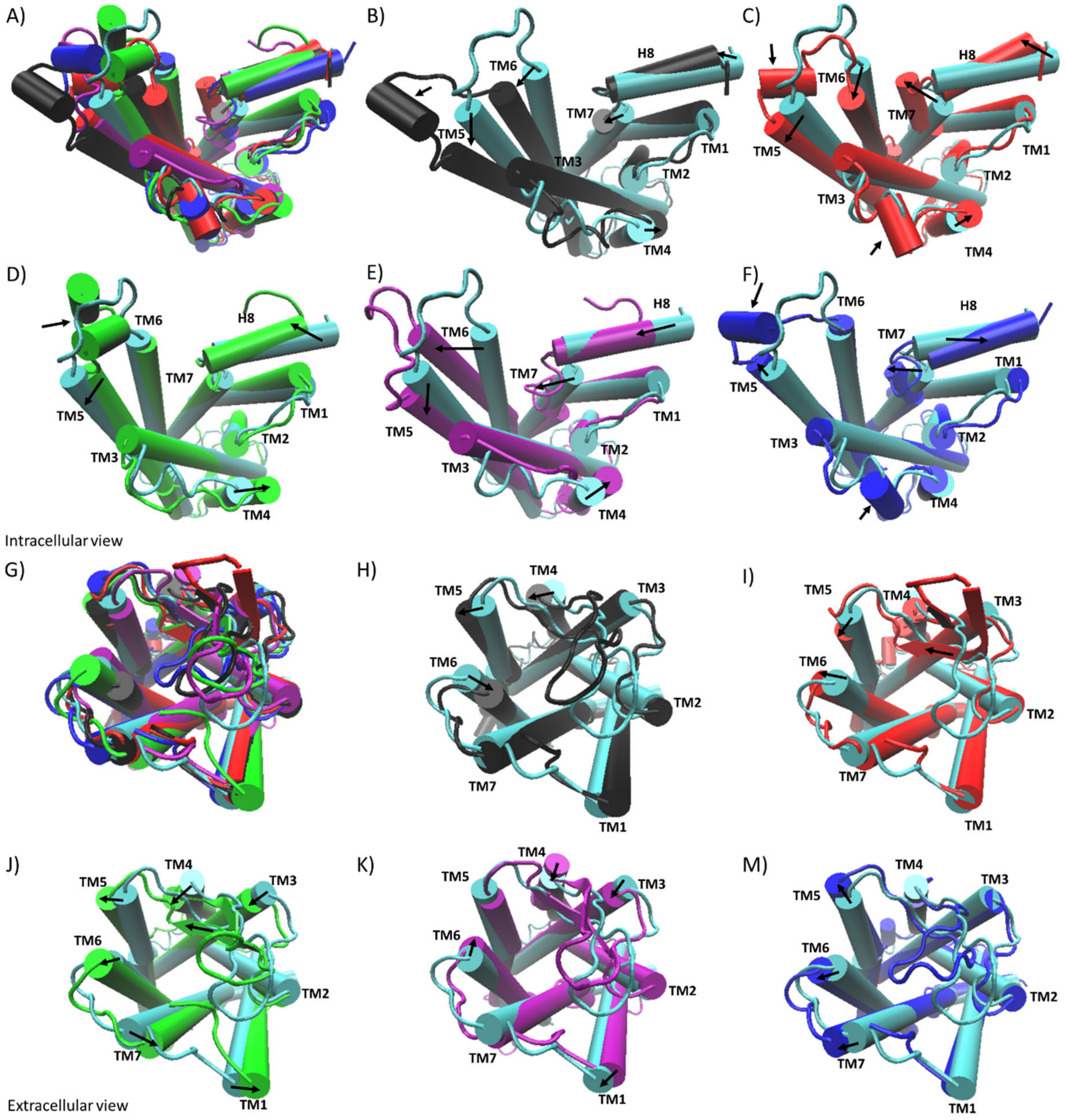

Figure S10. Intracellular view of experimental BK2R-Gq-bradykinin and A) all structures, B) BK2R-JSM-10292 (black), C) BK2R-alpha-tocotrienol (red), D) BK2R-amentoflavone (green), E) BK2R-isorhamnetin-3-O-rutinoside (purple), and F) BK2R-neoxanthin (blue). Extracellular view of BK2R-Gq-bradykinin and H) BK2R-JSM-10292, I) BK2R-alpha-tocotrienol, J) BK2R-amentoflavone, K) BK2R-isorhamnetin-3-O-rutinoside, and M) BK2R-neoxanthin.

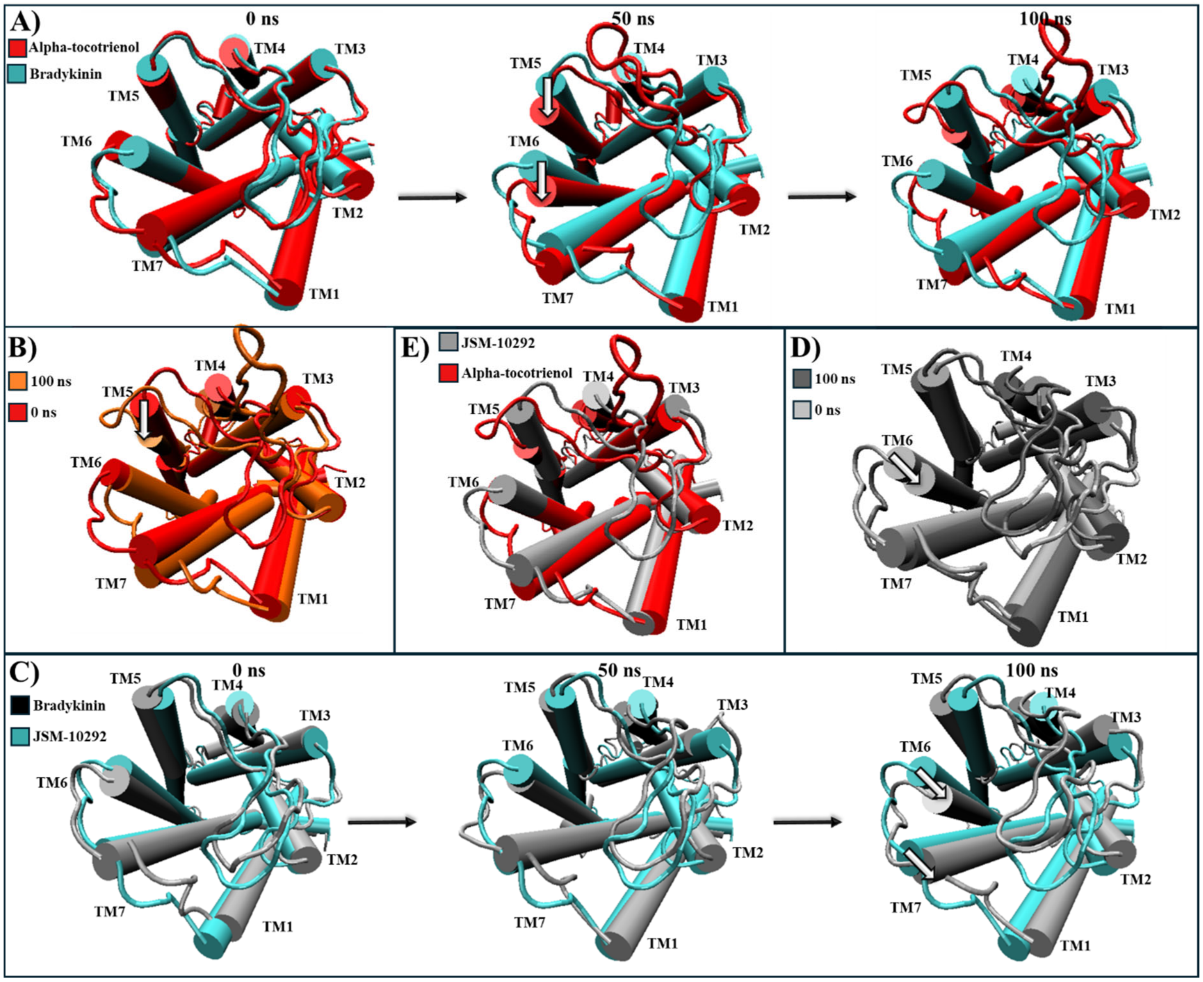

Figure S11. BK2R structural comparison at the extracellular view of A) alpha-tocotrienol complex (red) within 0, 50 and 100 ns and experimental structure of BK2R/bradykinin (cyan), B) alpha-tocotrienol complex at 0 ns (red) and 100 ns (orange), C) JSM-10292 complex (silver) within 0, 50 and 100 ns and experimental structure of BK2R/bradykinin (cyan), D) JSM-10292 complex at 0 ns (silver) and 100 ns (grey), E) alpha-tocotrienol complex (red) and JSM-10292 at 100 ns (silver).

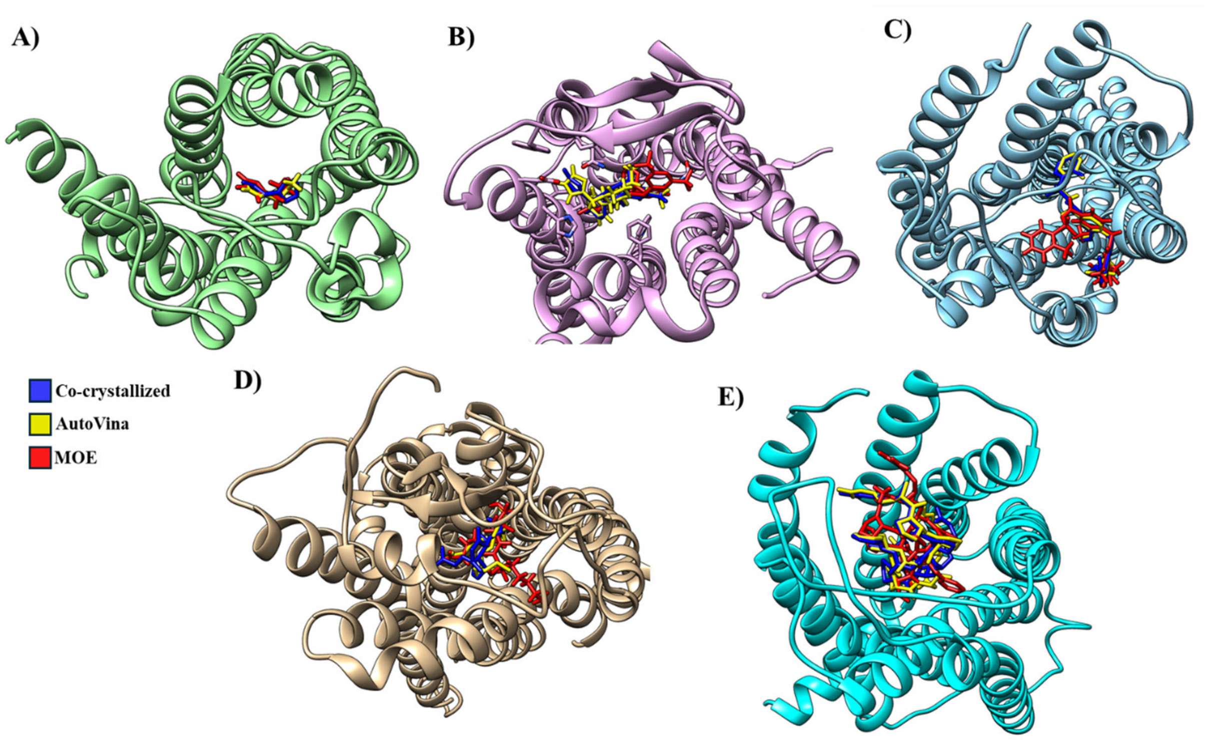

Figure S12. Illustration of the 3D re-docking process with the co-crystallized ligands for each receptor, utilizing both software platforms. A) HR1, B) NK1R, C) CLR1, D) CRTH2, and E) BK2R.

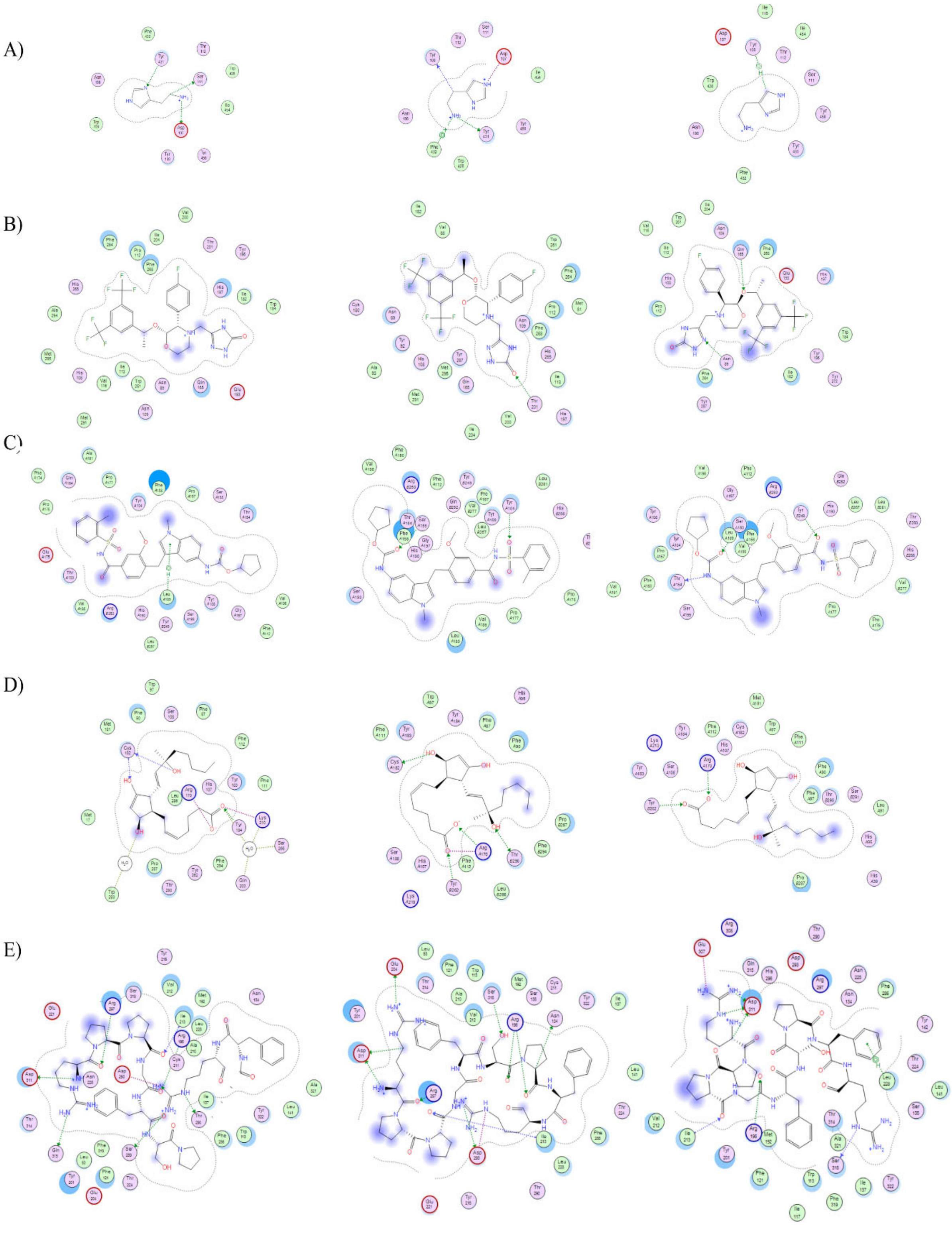

Figure S13. Illustration of the 2D re-docking process with the co-crystallized ligands (first image) utilizing Vina (second image, and MOE (last image). A) HR1, B) NK1R, C) CLR1, D) CRTH2, and E) BK2R.

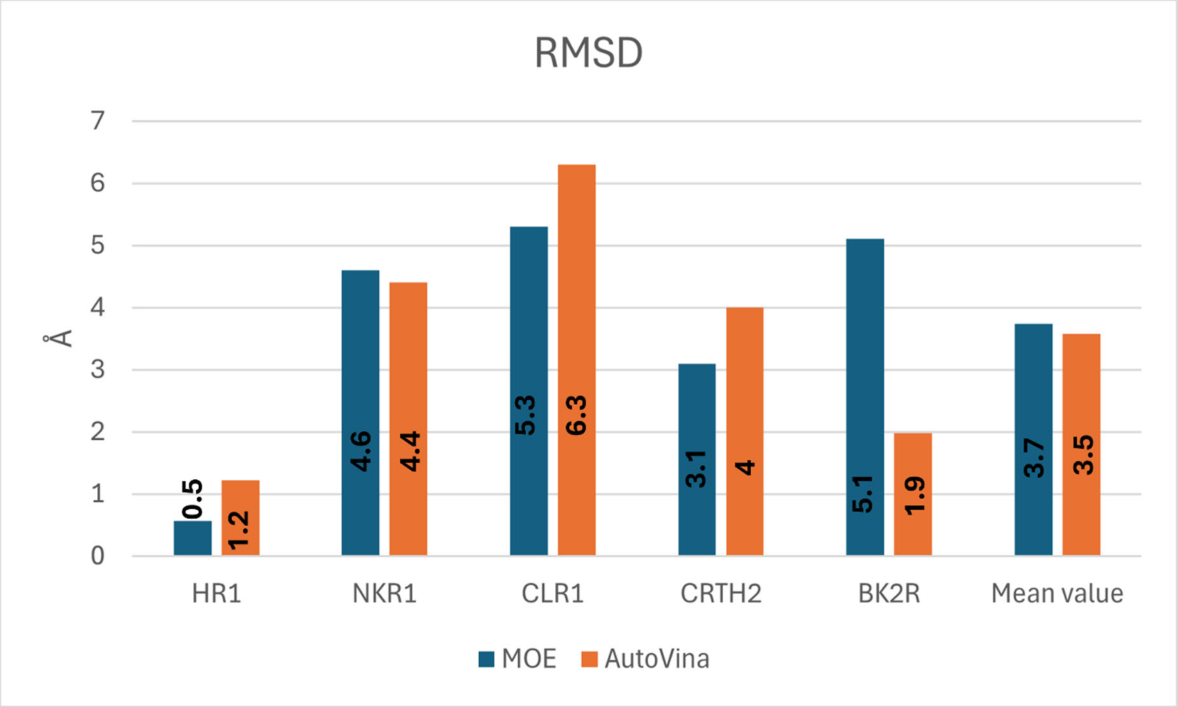

Figure S14. RMSD values for the redock.
